# Supplementary material for: A Workshop and Toolkit to Support Late-Career Transitions for Faculty
Source: MedEdPORTAL. 2024 Nov 12;20:11463. doi: 10.15766/mep_2374-8265.11463 (PMC11554777; doi:10.15766/mep_2374-8265.11463)
Supplement: Supplementary file 1 — Packet-Toolkit.docxToolkit Slides.pptxFacilitators Guide.docxWorkshop Evaluation.docx [file mep_2374-8265.11463-s001.zip › A. Packet-Toolkit.docx.docx]

**APPENDIX A:** **Packet/Toolkit**

**THIS IS A REMOVABLE COVER PAGE**

This page is for the Facilitators. Remove this page before creating handouts for participants.

What follows is the Packet/Toolkit for participants.

The packet/toolkit is an essential part of this workshop, facilitating activity participation and learning during the workshop and guiding continued work afterwards. We recommend making it accessible to the participants during the workshop with paper copies being best for ‘hands-on’ work and through a QR code for access after the workshop.

The packet/toolkit includes the following:

- - Activity Descriptions and Worksheets, in the order they are presented in the workshop
  - References and Resource Guide –extensive annotated bibliography organized by topic, e.g., attitudes and expectations influencing retirement, career paths, workforce issues, issues of aging and competency, institutional programs, and other resources.

**What to Do Next:**

**A Workshop and Toolkit for Late Career Transition Planning**

**Objectives: Upon completion, participants will be able to:**

- Self-evaluate personal challenges for making a late-career transition.
- Select elements of transition models that are meaningful for their own circumstances.
- Identify personal priorities and action strategies for late-career transitions.
- Identify resources that might be helpful to facilitate these transitions.

| **Insert here the names and contact information of presenters,  date and location of workshop** |
| --- |

**Tool in the Toolkit: Metaphors for Late Career Transition**

**Worksheet**

*originally developed by Judith Livingston PhD, Univ. Texas Health San Antonio, 2019*

Metaphor: First known use 15^th^ Century; from Greek *metapherein* “to transfer”; figurative language suggesting a likeness or analogy.

Examples from pediatricians in retirement transitions:

- - - “Ripping off a BandAid® slowly”
    - “Opening a window and letting in fresh air”

Instructions:

1. Take a few moments to think of a metaphor for your career transition at this point in time.
2. Write down your metaphor and/or draw an image of your metaphor in the box below.
3. Share what this metaphor says about where you are in transition and your feelings about transition.

**Tool in the Toolkit: The Evidence**

**Published Literature on Late-Career Transitions:**

(Bibliography is on pp 14-19)

| Note-taking |
| --- |

**Tool in the Toolkit: The Evidence**

**Frameworks and Resources for Career Transitions**

| Note-taking |
| --- |

**Tool in the Toolkit: The Whys and the Why Nots**

**Worksheet**

| **Instructions:** Take a few minutes on your own to think about each of the questions below and to jot down your responses. After a few minutes, you will have the opportunity to discuss your thoughts with others at your table. |
| --- |

| Why do I want to make a transition? |
| --- |
|  |
| Why am I reluctant to make a transition? |
|  |

**Tool in the Toolkit:**

**Card Sorting Task: Giving Up, Handing Over, Holding On**

*Originally developed by Virginia Niebuhr, PhD, Univ of Texas Medical Branch, 2019*

**Instructions:**

| You will need a stack of index cards (or small squares or strips of paper).  On each card (or piece of paper), write a task you currently do for your work. Include *all* the major and minor roles. Include all your clinical teaching roles, all your committee work, even those small tasks that only you do (e.g., putting together the slide show for the annual resident graduation banquet).  With this complete set, you can begin developing a transition plan for all your tasks.  You might start by sorting these into:  “What I am ready to give up” *vs.* “I need to keep this task.” |
| --- |

**Tool in the Toolkit: Case Discussions**

**Instructions:**

| We present you 2 case studies. Your group may choose to discuss one or both. Take about 15 minutes to discuss the case(s) with the persons in your group. The questions at the end of each case are designed to facilitate the discussion. We have also provided a grid of “transition issues.” You may wish to mark on the grid which issues are raised in each case.  *Cases developed by D Jaffe, P Wood, V Niebuhr, F Henretig, J Livingston* |
| --- |

**CASE 1. Dr. James**

Dr. Carol James (66) has been on the medical school faculty for 30 years of her 36-year career. She sometimes thinks about retiring, but usually is just too busy to think about it until someone asks her “when will you be retiring?” She is never quite sure if this is asked as a nudge (“you really should retire, you are getting too old to do this work”) or as a plea to stay on (“you’re not thinking of leaving, are you?”). She has had a successful research career with continuous extramural funding for 20 years, and she especially enjoys mentoring the post-doctoral fellows in her lab. However, she has also been thinking about the stress of having to maintain her funding and support her research personnel. She has begun to wonder how she might eventually transition these responsibilities to other colleagues. Dr. James sometimes worries if there will be enough money in retirement for her to do what she wants. The thought of relying entirely on a market-based IRA sometimes keeps her up at night. She has a history of breast cancer- now cured – and she knows she will need to transition her health insurance when she retires.

She has always said “one should retire TO something, not FROM something,” and she has not yet figured out what that will be for her. Her spouse retired two years ago, has jumped into all sorts of community activities and wants her to retire so that they can spend quality time together. She wants to do that, but also wants to find her own passions in retirement. She realizes how all-consuming her academic career has been.

Her parents are still living (ages 91 and 93) and she knows there will soon be a time when she will need to take care of them. She wonders if she should retire before that time comes so she will have all the time she needs for their care, or should she continue to work and use FMLA when needed?

**Questions:**

1. What challenges of transition is Dr. James facing?
2. As a friend and colleague, what advice do you have for her (if asked)?
3. What are the considerations regarding her spouse? parents? self?
4. Which of Dr. James’ issues resonate with your own thoughts about transition?

| **Transition Issues** (not all cases will touch on all issues) | **comments** |
| --- | --- |
| Aging parents |  |
| Clinical competence |  |
| Clinical re-entry |  |
| Continued relationship with institution (emeritus? Some kind of appointment?) |  |
| Establishing a legacy |  |
| Family commitments |  |
| Health insurance concerns |  |
| Identity-who will I be? |  |
| Institutional guidance/support |  |
| Keeping up with content and technology |  |
| Loss of rewards/recognition |  |
| Retired spouse |  |
| Retirement income concerns |  |
| Satisfaction w/ making a difference |  |
| Stamina |  |
| Other |  |

**CASE 2. Dr. Anderson**

Dr. Kim Anderson is a 68-year-old academic physician. He embarked on his medical career at the age of 39, after working in public health. He made a commitment to practice medicine at least 25 years because he knew that he was taking a spot that another applicant, probably younger, would have had. Dr. Anderson holds leadership positions in his institution and in state and national organizations, helping to advance policies with far-reaching benefits to children and their families. He has earned many accolades and continues to derive great satisfaction from his work, especially teaching and mentoring. Recently he took a Sabbatical year away from clinical work to serve as president of a major national organization. In contemplating his return to clinical practice, he worries about the combined effects of age and time away from active practice (pediatric intensive care medicine). In addition, Dr. Anderson is increasingly disenchanted with institutional burdens and the relentless chore of EMR. His spouse, who retired a few years ago, continues to question him about retiring, but also understands and supports his desire to set his own pace for transition. Dr. Anderson has mixed emotions about giving up a career he loves but sees the value of spending more time with family.

**Questions**

1. What are Dr. Anderson’s transition issues?
2. As a friend and colleague, what advice would you give Dr. Anderson (if asked)?
3. Is this an either/or choice for Dr. Anderson? Might he be able to explore a transition plan that preserves activities he likes while divesting the activities he finds burdensome? What if he is asked to do these without pay?
4. What threats to personal and professional identity is Dr. Anderson facing? How might he manage these successfully?
5. Which of Dr. Anderson’s issues resonate with your own thoughts about transition?

| **Transition Issues** (not all cases will touch on all issues) | **comments** |
| --- | --- |
| Aging parents |  |
| Clinical competence |  |
| Clinical re-entry |  |
| Continued relationship with institution (emeritus? Some kind of appointment?) |  |
| Establishing a legacy |  |
| Family commitments |  |
| Health insurance concerns |  |
| Identity-who will I be? |  |
| Institutional guidance/support |  |
| Keeping up with content and technology |  |
| Loss of rewards/recognition |  |
| Retired spouse |  |
| Retirement income concerns |  |
| Satisfaction w/ making a difference |  |
| Stamina |  |
| Other |  |

**Tool in the Toolkit: Personal Framing of Challenges & Priorities**

**Worksheet**

**Instructions:**

| Here are some transition issues to consider. Please take a few minutes to reflect on them. You may wish to write a word or phrase in the reflections box provided. Also please feel free to add other issues that are important to you. There will be a few minutes to share your reflections in discussion with others at your table. We welcome you to use this worksheet in a deeper personal reflection at home. | |  |  |
| --- | --- | --- | --- |
| **Transition Issues** | | **Reflections** | |
| 1. Continued relationship with institution (e.g., change of appointment? teaching? advising/mentoring? emeritus?) | |  | |
| 1. Establishing a legacy; handing off precious projects | |  | |
| 1. Family commitments: life partner, children, aging parents, grandchildren, other | |  | |
| 1. How will I handle potential loss of rewards/recognition? | |  | |
| 1. Identity – Thinking about who I am now; who will I be? | |  | |
| 1. Loss of professional friendships/relationships | |  | |
| 1. Is there good institutional guidance/peer support for making the transition? And do I know where to go for help? | |  | |
| 1. Am I keeping up-to-date with content, technology, policies? How might this change after transition/retirement? | |  | |
| 1. Are there concerns about health benefits? Insurance issues? | |  | |
| 1. Are there income concerns? | |  | |
| 1. Satisfaction with making a difference. Will there be new ways to make a difference? | |  | |
| 1. Stamina | |  | |
| 1. Personal health, health of life-partner | |  | |
| 1. Other(s) | |  | |

Which of the issues are the top challenges/priorities for me?

Which of these had I not considered?

**Tool in the Toolkit: Institution-Specific Considerations**

Note-taking

| **Ask Questions**   - What is possible? - What decisions need to be made? - When? - Where is the information? - Who is the ‘truth-keeper’? (the one at the institution with the correct answers)   **What type of transition do you want to make?**  **What type of transition is possible?**   - Retirement vs. Part Time? - FTE change? - Focus change?   **‘Who can help?’**   - Supervisor - Dept. Resources - Office Academic Affairs & Career Development - HR - benefits - Financial advisor - Pension plan/401K - Peer Guide? Someone recently transitioned/retired - Professional Coach (see Toolkit)   **Issues to consider**   - Rules of your retirement plan? - Salary? Pay-by-Letter (hourly)? Volunteer? - Rank/Title change   Tenure to nontenure? Adjunct?   Emeritus? - Retirement privileges?   email, library privileges, parking - Credentialing - Funder requirements: grants, endowments, etc. - EHR access - Email access - Pension plan/401K- financial advisor - Health insurance - Accrued leave-time, unused vacation |  |
| --- | --- |

**Tool in the Toolkit: Personal Action Plan**

**Worksheet**

What are my SPECIFIC next steps?

- Do I need to plan more personal reflection time?
- Do I need to gather more information? If so, what specific information and from what source(s)?
- Do I need to refine my priorities?
- Do I need to get more advice? If so, what specific advice and from whom?
- Do I need to “talk it out” with someone? If so, with whom?
- Do I need to move forward with a specific plan? If so, what is the NEXT STEP in that process?

| **Within 5 weeks (Date: ), I will**: | **Within 3 months (Date: ), I will:** |
| --- | --- |
|  |  |
| **What resources are needed?** | **What resources are needed?** |

**Tool in the Toolkit: Coaching**

**What is coaching and how can it be helpful when planning late career transitions?**

**[Note: this is a supplemental resource for participants and not a workshop activity]**

*Developed by Andrew Sirotnak, MD, Univ. Colorado SOM, 2023*

Information above adapted from: <https://experiencecoaching.com/learn>

**Coaching matters in our workplaces now: some evidence-based core principles.**

- Coaching promotes creativity, breakthrough performance and increased resilience.
- Successful coaching adds value to employees; builds and enhances teamwork and group performance; motivates productivity and higher levels of job satisfaction; improves management and leadership skills; and promotes diversity & inclusion awareness.
- There are many coaching models and choosing the one that fits the person or team and goals starts with a conversation about what program is available and then interviews with potential coaches to determine a best fit.
- Coach is acting as a change agent, creating awareness through evocative questions, helping the coachee develop roadmap & strategies to remove potential roadblocks, and move forward towards the desired goal.
- A properly trained and experienced coach will be skilled at active listening, asking thought provoking and powerful questions, will use deliberate, mindful speech clarifying and reframe as needed, and ask permission to give feedback or any advice.

**What questions might you expect to hear from a coach when engaged in a discussion on late career transitions? You might hear powerful effective questions that are designed to:**

- Reflect active listening that invites you to share your points of view; and discuss personal values, motivations or habits that could inform your transition decision(s).
- Allow for increased clarity and create new possibilities for your future.
- Generate new insights, new perspectives, and new commitments.
- Enable you to advance to your objective / decision making, instead of looking back or making justifications about the past as you think about the future.
- Prompt you to think about what still motivates / excites you; how you define legacy or career impact; and connecting these to your values as you plan a career transition.

*Coaching, continued*

**What topics might you and your peers as potential clients bring to a coach?**

| **WELL BEING** | **CAREER**  **DEVELOPMENT** | **LEADERSHIP DEVELOPMENT** | **CAREER TRANSITIONS** |
| --- | --- | --- | --- |
| Life balance and fulfillment | Career path decisions | Communication skills | **Career path decisions** |
| Resilience tools | Healthy relationships with colleagues | Teamwork and delegation | **Creating legacy** |
| Reduced burnout | Engagement in work | Emotional intelligence | **Reaffirming values and goals** |
| Values clarification | Time management | Influencing and leading change | **Succession planning** |
| Health and physical wellness | Overcoming obstacles | Organizational skills | **Retirement** |

Use this space for thoughts on using coaching as a support for late career transition planning. Think about how you could also use powerful questions in your dialogues with peers in discussing their late career transitions or retirement. What would you ask them?

**Tool in the Toolkit: Bibliography**

Highlighted entries are key references

**TRANSITION (Books and other resources)**

Anderson ML, Goodman J, & Schlossberg NK. *Counseling adults in transition: Linking Schlossberg’s theory with practice in a diverse world.* 4th ed. New York, NY: Springer; 2012.

Baldwin RG ed. Reinventing Academic Retirement. *New Directions for Higher Education* (Wiley on-line journal) Summer 2018: no. 182. <https://www.hopkinsmedicine.org/-/media/the-academy/reinventing-academic-retirement.pdf>

Bridges W. *Transitions: Making sense of life’s changes.* 2^nd^ ed. Cambridge, MA: Da Capo Press; 2004.

Connolly SM. Late Career Solutions. Transitional Zone–Retire or Retread. In: Stonnington CM, Files JA ed. *Burnout in Women Physicians: Prevention, Treatment, and Management.* Springer; 2020: 535-552. <https://doi.org/10.1007/978-3-030-44459-4_17>

Freedman M. *The big shift: Navigating the new stage beyond midlife*. New York, NY: Public Affairs; 2011.

Ibarra H. *Identity transitions: Possible selves, liminality and the dynamics of career change.* Working Paper Series. Fontainebleau, France: INSEAD, 2007.

Laslett P. *A fresh map of life: The emergence of the third age.* Cambridge, MA: Harvard University Press, 1991.

Livingston J. *Career transitions in the third age: A study of women pediatricians.* Dissertation*.* Texas State University; 2017. Retrieved from ProQuest Dissertations & Theses Global. (Order No. 10737136).

Powell DH, Whitla DK. *Profiles in Cognitive Aging*. Cambridge, MA: Harvard University Press; 1994.

- Primary source of figures in Dellinger et al, 2017

Schlossberg NK. *Revitalizing retirement: Reshaping your identity, relationships, and purpose*. Washington, DC: American Psychological Association; 2009.

Sugarman L. *Life-span development: Frameworks, accounts and strategies*. New York, NY: Taylor & Francis, Inc.; 2001.

Turner VW. *The Forest of Symbols*. Ithaca, NY: Cornell University Press; 1967.

Van Gennep A. *The Rites of Passage*. London, United Kingdom: Routledge & Kegan Paul; 1960.

**ADVICE ABOUT RETIREMENT**

Heyl AR. The transition from career to retirement: focus on well-being and financial considerations*. J* *Am Medical Women’s Assoc* 2004; 59(4): 235-7. [General advice and commentary; focused on women]

Pizzo PA. Navigating transitions and charting new paths. JAMA 2017; 317:1625-6.

- Thoughtful essay on challenges of the “transition” and the dangers of denial of loss of skills

Ramamurthy R. The exit: retiring from academic medicine, Part 1. *San Antonio Medicine*, Sept 2015: 18-20. Accessed at: <https://issuu.com/louisdoucette/docs/0915_sam_final> [practical, humorous advice]

**ATTITUDES, EXPECTATIONS, and FACTORS INFLUENCING RETIREMENT**

Altman Y, Baruch Y, Zoghbi Manrique-de-Lara P, Viera Armas MM. Baby boomers at the cusp of their academic career: Storming ahead, Hanging on, or Calling it a day*. Studies in Higher Education*. 2020;45(7):1335-50.

Bickel J. Not too late to reinvigorate: How midcareer faculty can continue growing. *Academic Medicine*. 2016;91(12):1601-5. [Opinion piece: strategies to facilitate reflection & critical self-inventory]

Boveda I, Metz AJ. Predicting end‐of‐career transitions for baby boomers nearing retirement age. *The Career Development Quarterly*. 2016;64(2):153-68.

Cahill M, Galvin R, Pettigrew J. The retirement experiences of women academics: a qualitative, descriptive study. *Educational Gerontology*. 2021;19:1-5.

Cahill, M., Pettigrew, J., Robinson, K., & Galvin, R. The transition to retirement experiences of academics in “higher education”: A meta-ethnography. *The Gerontologist*, 2018;59(3), e177-e195. <http://doi.org/10.1093/geront/gnx206>

Cleland J, Porteous T, Ejebu OZ, Skåtun D. ‘Should I stay or should I go now?’: A qualitative study of why UK doctors retire. *Medical Education*. 2020 Sep;54(9):821-31.

Collins RT, Sanford R. The Importance of Formalized, Lifelong Physician Career Development: Making the Case for a Paradigm Shift. *Academic Medicine*. 2021 Sep 28;96(10):1383-8.

Ellis C, Allen M, Bochner AP, et al. Living the post-university life: Academics talk about retirement. *Qualitative Inquiry*, 2017;23(8):575-588. <https://doi.org/10.1177/1077800417716392>

Hedden L, Lavergne MR, McGrail KM, et al. Patterns of physician retirement and pre-retirement activity: a population-based cohort study. *CMAJ*. 2017;189(49):E1517-23.

Kadefors R, Schaller J, Thång PO, Pestat E. Attitudes among male and female university professors, and other categories of university employees, to working up to and beyond normal retirement age. *Nordic Journal of Working Life Studies*. 2016;6(1):133-46.

Kojola E, Moen P. No more lock-step retirement: boomers' shifting meanings of work and retirement. *Journal of Aging Studies* 2016, *36*: 59-70. [in-depth interviews with working and retired white-collar “Boomers”]

Klag M, Jansen KJ, & Lee MD (2015). Contemplating workplace change: Evolving individual thought processes and emergent story lines. *The Journal of Applied Behavioral Science*, *51*(1), 36-70. [In-depth interviews: 26 Canadian public health physicians]

Marcdante K, Knox K, Amidon M. Preparing for the Transition to Your Next Career Role. *Journal of Graduate Medical Education*. 2020;12(1):109-10.

Maurer TJ, Chapman EF. Relationship of proactive personality with life satisfaction during late career and early retirement. *Journal of Career Development*. 2018 Aug;45(4):345-60.

Onyura B, Bohnen J, Wasylenki D, et al. Reimagining the self at late-career transitions: how identity threat influences academic physicians’ retirement considerations. *Academic Medicine* 2015; 90:794-781.

- Focus groups with senior Canadian physicians (N=21); identified major themes

Rimsza ME, Ruch-Ross H, Simon HK, Pendergass TW, Mulvey HJ. Factors influencing pediatrician retirement: a survey of AAP chapter members*. J Pediatr* 2017; 188:275-9. [Survey of AAP members; reasons to retire]

Silver MP, Pang NC, Williams SA. “Why give up something that works so well?”: retirement expectations among academic physicians. *Educational Gerontology* 2015. 41(5):333-347. [focus groups n=16 Canadian acad physicians]

Silver MP, Williams SA. Reluctance to retire: a qualitative study on work identity, intergenerational conflict, and retirement in academic medicine. *The Gerontologist*. 2018;58(2):320-30.

Skarupski KA, Roth DL, Durso SC. Prevalence of caregiving and high caregiving strain among late-career medical school faculty members: workforce, policy, and faculty development implications. *Human Resources for Health*. 2021;19(1):1-9.

Skarupski KA, Welch C, Dandar V, Mylona E, Chatterjee A, Singh M. Late-career expectations: a survey of full-time faculty members who are 55 or older at 14 US medical schools. *Academic Medicine*. 2020;95(2):226-33.

Smith F, Lachish S, Goldacre MJ, Lambert TW. Factors influencing decisions of senior UK doctors to retire or remain in medicine. *BMJ Open* 2017; 7:e017650. [Survey of British physicians]

Steele RW. Why do pediatricians retire? *Clin Pediatrics* 2015; 54: 1309-10. (Commentary)

Sweeney JF. Physician retirement. Med Economics 2019; 96(4): 12-17. [1^st^ person narratives; data on concerns]

Swinnen A, Honné H, Malafei I, Stieber M. “Care is what you miss from the organization”: Experiences of pre-and post-retirement academic staff at Maastricht University. 2020. [www.researchgate.net/publication/357242319](http://www.researchgate.net/publication/357242319)

Zacher H, Rudolph CW, Todorovic T, Ammann D. Academic career development: A review and research agenda. *Journal of Vocational Behavior*. 2019;110:357-73.

**CAREER PATHS**

Connolly SM. Late Career Solutions. Transitional Zone – Retire or Retread. In: Stonnington C., Files J. (eds) *Burnout in Women Physicians: Prevention, Treatment, and Management*. Springer;2020: 535-552. <https://doi.org/10.1007/978-3-030-44459-4_17>

Hall JG. The challenge of developing career pathways for senior academic pediatricians. *Pediatr Res* 2005. 57(6):914-19. [survey of APS members and pediatric chairs; desires and opinions (35-40% response rate]

Greenberg L. Can the recruitment of senior transitioning clinician educators enhance the number and quality of resident observations: Thinking outside the box. *Teaching and Learning in Medicine*. 2020; 32(5):569-74

Hesham HT, Grundfast K, Sarber K. Making a Major Change: Changing Your Practice Setting, Retirement, and Locums. *Otolaryngologic Clinics of North America*. 2022;55(1):33-41.

Moss AJ, Greenberg H, Dwyer EM, et al. Senior academic physicians and retirement considerations. *Progress in Cardiovascular Diseases* 2013; 55(6):611-615. [roles for academic medical faculty moving into their senior years]

Orwoll E. Passing the baton- harnessing the full value of older scientists. *N Engl J Med* 2016. 274:2514-5.

Silver MP, Easty LK. Planning for retirement from medicine: a mixed-methods study. *CMAJ open*. 2017;5(1):E123.

Silver MP, Hamilton AD, Biswas A, Warrick NI. A systematic review of physician retirement planning. *Human Resources for Health*. 2016; 14:1-16. [Systematic review: 65 studies of timing & process of physician retirement]

Stearns J, Everard KM, Gjerde CL, et al. Understanding the needs and concerns of senior faculty in academic medicine: building strategies to maintain this critical resource. *Academic Medicine* 2013; 88(12), 1927-1933. [online survey of U.S. family physicians in academic medicine ≥ 50 years old (n=768)]

Strange Khursandi D, Eley V. ‘Quit while you are ahead–and smell the roses!’ A survey of retired Fellows of the Australian and New Zealand College of Anaesthetists. *Anaesthesia and Intensive Care*. 2021;49(5):379-88.

Templeton K, Nilsen KM, Walling A. Issues faced by senior women physicians: a national survey. *Journal of Women's Health*. 2020;29(7):980-8

**WORKFORCE ISSUES**

Association of American Medical Colleges. U.S. Medical School Faculty Trends: Average Age of Chairs and Full-time Faculty by Gender and Rank, 1966 through 2022. AAMC Faculty Roster, 2022 snapshot. Updated December 31, 2022. Accessed November 21, 2023. <https://www.aamc.org/media/54641/download>.

Association of American Medical Colleges. Supplemental Table F: Faculty by Gender, Rank, and Age Group. AAMC Faculty Roster, 2022 Report. Updated December 31, 2022. Accessed November 21, 2023. <https://www.aamc.org/data-reports/faculty-institutions/data/2022-us-medical-school-faculty>

Beeler WH, Mangurian C, Jagsi R.  Unplugging the Pipeline - A Call for Term Limits in Academic Medicine. [*N Engl J Med*.](https://www.ncbi.nlm.nih.gov/pubmed/31618538) 2019 Oct 17; 381(16):1508-1511. <https://www.nejm.org/doi/full/10.1056/NEJMp1906832> [issues of diversity of leadership]

Hedden L, Lavergne MR, McGrail KM, Law MR, Cheng L, Ahuja MA, Barer ML. Patterns of physician retirement and pre-retirement activity: a population-based cohort study. *CMAJ* 2017; 189:E1517-23. [Canadian physicians ≥50 years]

Merline AC, Cull WL, Mulvey HJ, & Katcher AL. Patterns of work and retirement among pediatricians > 50 years. *Pediatrics* 2010: *125*, 158-64. [cross-sectional mail survey of AAP members (n=1,114)]

Mok C, Boneham W, Lennon MJ. A ‘healthy’ health care workforce: Insights into satisfaction and retention of doctors. *Medical Education*. 2020;54(9):781-3.

Mulvey HJ, Jewett EA, Merline A, Towey KJ. Pediatricians over 50 reentering clinical practice: implications for physicians and the regulatory community. *J Medical Regulation*.2010; 96(2):7-12. [Survey AAP members >55]

Petterson SM, Rayburn WF, Liaw WR. When do primary care physicians retire? Implications for workforce projections. *Ann Fam Med* 2016; 14:344-9. [Data from AMA Masterfile (2010-2014; longitudinal)]

Spector ND, Cull W, Daniels SR, et al. Gender and generational influences on the pediatric workforce and practice. *Pediatrics* 2014; 133(6):1112-1121. [Discussion of generational differences and changes in professional life (from: Federation of Pediatric Organizations’ Gender and Generations Working Group]

**EFFECT ON PATIENTS**Lam K, Arnold CG, Savage RD, et al. Does physician retirement affect patients? A systematic review. *Journal of the American Geriatrics Society*. 2020;68(3):641-9.

**PHYSICIAN COMPETENCY/ ISSUES OF AGING**

Armstrong KA, Reynolds EE. Opportunities and challenges in valuing and evaluating aging physicians. JAMA 2020; 323(2): 125-126. [Commentary on Cooney L and Balcezak T. JAMA 2020]

Bazzo DE, Smith P, Wenghofer EF. A Pilot Study of a Screening Process for Evaluating the Physical, Mental and Cognitive Health of Senior Physicians. *Journal of Medical Regulation*. 2021;107(2):33-40.

Choudhry NK, Fletcher RH, Soumerai SB. Systematic review: the relationship between clinical experience and quality of health care. *Ann Intern Med* 2005; 1452: 260-273. [Systematic review; little data supports improved performance with increasing age (rather the opposite)]

Cooney L, Balcezak T. Cognitive testing of older clinicians prior to recredentialing. JAMA 2020; 323(2): 179-180. [Description of program to evaluated applicants for re-appointment to medical staff who were aged 70 years or older (Yale-New Haven)]

Dellinger EP, Pellegrini CA, Gallagher TH. The aging physician and the medical profession: a review. *JAMA Surgery* 2017. 152:967-971. [Review of studies linking age, cognitive functioning and clinical outcomes]

Drag LL, Bieliauskas LA, Langenecker SA, Greenfield LJ. Cognitive functioning, retirement status, and age: results from the Cognitive Changes and Retirement among Senior Surgeons study. *J Am Coll Surg.* Sep 2010; 211(3):303-307. [Cognitive testing results (by age groups): surgeons attending national conferences]

Durning SJ, Artino AR, Holmboe E, et al. Aging and cognitive performance: challenges and implications for physicians practicing in the 21^st^ century. *JCEHP* 2010; 30: 153-160. [conceptual model for physician aging and clinical competencies]

Fitzgerald FT, LaCombe MA. New Orleans al Fresco. *Ann Intern Med* 2013; 158: 293-294. [essay about aging and failing skills; asking trusted younger colleague to keep an eye on you]

Hawkins RE, Welcher CM, Elliot VS, et al. Ensuring competent care by senior physicians. *JCEHP* 2016, 36: 226-231. [Summary of 2015 AMA report (comprehensive review of aging and physician workforce): <https://www.cppph.org/wp-content/uploads/2016/02/AMA-Council-on-Medical-Education-Aging-Physician-Report-2015.pdf> ]

Henretig FM, Wood JN, Shea JA, Ruddy RM. PEM physicians’ perceptions of colleagues’ clinical competencies over career span. *Ped Emerg Care* 2022; 39(5):304-310. DOI : [10.1097/pec.0000000000002785](https://doi.org/10.1097/pec.0000000000002785)  PMID: 35766881  [Survey of 478 PEM physicians, finding PEM physicians > 65 perceived as less capable with resuscitative skills, but just as capable, or more so, with humanistic aspects]

Jauhar S. How would you feel about a 100 year old doctor? *New York Times.* Nov 28, 2022; p.22.

Kaups KL. Competence not age determines ability to practice: ethical considerations. *AMA J Ethics* 2016. 18:1017-24. [Summary of data: physician performance and age; institutional screening policies]

Kupfer JM. The graying of US physicians: implications for quality and the future supply of physicians. *JAMA* 2016. 315(4): 341-2. [Viewpoint] [Data on aging/compete; opinion: ABMS should develop a single, integrated national standard for practice eval, recert and MOC.]

Mouitier CY, Bazzo DEJ, Norcross WA. Approaching the issue of the aging physician population. *J Medical Regulation* 2013; 99: 10-18. [Data from 2011 national conference of experts in medicine, law and public health]

Saver JL. Best practices in assessing aging physicians for professional competency. JAMA 2000;323(2): 127-129.

- Commentary on Cooney L and Balcezak T. JAMA 2020.

Sherwood R, Bismark M. The ageing surgeon: a qualitative study of expert opinions on assuring performance and supporting safe career transitions among older surgeons. BMJ Qual Saf 2020; 29: 113-121.

- Focus groups & survey of experts: concern for declining performance with age. Suggest a tiered, collaborative approach to ensuring competence.

Span P. When is the surgeon too old to operate? *NY Times*. Feb 1, 2019. Accessed at: <https://www.nytimes.com/2019/02/01/health/surgeons-retirement-competence.html>

Tarkan L. As doctors age, worries about their abilities grow. *NY Times*. Jan 24, 2011.  Accessed at: <https://www.nytimes.com/2011/01/25/health/25doctors.html>

Tsugawa Y, Newhouse JP, Zaslavsky AM et al. Physician age and outcomes in elderly patients in the US; observational study. *BMJ* 2017; 357:j1797.doi:10.1136/bmj.j1797. [patient mortality by age & practice volume of treating physician]

Waljee JF, Greenfield LJ, Dimick JB et al. Surgeon age and operative mortality in the United States. *Ann Surg* 2006; 244: 353-362. [Differences in operative mortality differences by age of surgeon]

**COACHING
BOOKS***:*

*Co-Active Coaching: The proven framework for transformative conversations at work and in life*. 2018; Fourth edition; by authors Karen Kimsey-House, Henry Kimsey-House Phillip Sandhal and Laura Whitworth

Riddle DD, Hoole ER, Gullette ECD, eds. The Center for Creative Leadership Handbook of Coaching in Organizations. Jossey-Bass; 2015.

Whitmore J. Coaching for Performance:  The Principles and Practice of Coaching and Leadership. 5th Edition (Updated 25th Anniversary Edition) ed. Nicholas Brealey; 2017.

**WEB-BASED RESOURCES:**

[International Coaching Federation- Professional Coaching Association](https://coachingfederation.org/). Download free guide and explore frequently asked questions such as: What is professional coaching? How is coaching different from mentoring or therapy? Who is coaching for? What are the signs that I might benefit from coaching? How do I find a qualified coach? What should I expect from a coaching session?

Qadir XC, Kassauei K, Norz b, Sarraf KM. The Healthcare Industry:  Ready For Coaching? 2020. 11/17/2020. <https://instituteofcoaching.org/blogs/healthcare-industry-ready-coaching>

**ARTICLES:**

Boet S, Etherington C, Dion PM, Desjardins C, Kaur M, Ly V, Denis-LeBlanc M, Andreas C, Sriharan A. Impact of coaching on physician wellness: A systematic review. *PLoS One.* 2023 Feb 7;18(2):e0281406. Access at: [10.1371/journal.pone.0281406](https://doi.org/10.1371/journal.pone.0281406)

Coutu D & Kauffman C. (2009). What can coaches do for you? Harvard Business Review 87(1), 91-97.

Dyrbye LN, Gill PR, Satele DV, West CP. Professional Coaching and Surgeon Well-being: A Randomized Controlled Trial. *Ann Surg.* 2023 Apr 1;277(4):565-571.

Dyrbye LN, Shanafelt TD, Gill PR , Satele DV , West CP. Effect of a Professional Coaching Intervention on the Well-being and Distress of Physicians: A Pilot Randomized Clinical Trial. *JAMA Intern Med* 2019; 179(10):1406-1414.

Gazelle G, Liebschutz JM, Riess H. Physician burnout: coaching a way out. *Review J Gen Intern Med* 2015 Apr;30(4):508-13.

Stephany AM, Archuleta P, Sharma P, Hull SK. Professional Coaching in Medicine and Health Care. *Clin Sports Med. 2023* Apr;42(2):195-208.

Tawfik DS, Profit J, Morgenthaler TI, et al. Physician Burnout, Well-being, and Work Unit Safety Grades in Relationship to Reported Medical Errors. *Mayo Clinic Proceedings*. 2018;93(11):1571-1580. doi:10.1016/j.mayocp.2018.05.014

Winters R. Coaching Physicians to become leaders. *Harvard Business Review*. 2013

**INSTITUTIONAL RESPONSES, RESOURCES AND PATHWAYS FOR LATE-CAREER TRANSITIONS**

Baumgartner W, Ziminski C, VanBeek J, Rand C. Everyone wins when retiring faculty of health professions schools are supported. *Acad Med* 2021; 96(10): 1375.

Cain JM, Felice ME, Ockene JK, et al. Meeting the late-career needs of faculty transitioning through retirement: one institution’s approach. *Acad Med* 2018; 93:435-439. [U Mass: policies, programs, resources for late-career faculty]

Plotnick LH, Sternszus R, Macdonald ME, Steinert Y. Engaging retired physicians as educators: motivations and experiences of participants in a novel educational program. *Acad Med* 2022;97(12):1842-1846. [Innovative program: Quebec]

Richards R, McLeod R, Latter D, et al. Toward late career transitioning: a proposal for academic surgeons. *Can J Surg* 2017; 60:355-358. [Guidelines and process for collaborative development of individual plans for late career transition (U Toronto); includes information about science of aging in surgeons]

Skarupski KA, Dander V, Mylona E, et al. Late-career faculty: a survey of faculty affairs and faculty development leaders of U.S. Medical schools. *Academic Medicine* 2020; 95: 234-240.

Zeig MJ, Baldwin RG. Keeping the Fire Burning: Strategies to Support Senior Faculty. *To Improve the Academy: A Journal of Educational Development* 2013; 32. Access at: <https://quod.lib.umich.edu/t/tia/17063888.0032.009?view=text;rgn=main>

**RETIREMENT/TRANSITION PROGRAMS AT SPECIFIC INSTITUTIONS**

**U Mass Chan Medical School Office of Faculty Affairs:** <https://www.umassmed.edu/ofa/development/flexibility/transition/>

**Stanford University School of Medicine**

Iris Litt, MD, Associate Dean for Senior and Retired Faculty

For senior and retired School of Medicine Faculty:

<https://med.stanford.edu/academicaffairs/faculty/resources-for-senior-and-retired-faculty.html>

**Johns Hopkins School of Medicine**

Cynthia Rand, PhD, Senior Associate Dean for Faculty

<https://www.hopkinsmedicine.org/fac_development/sr-faculty-retirement-resources/index.html#transition>

**WORKSHOPS Presented.**

Late Career Transitions Workshops

*Peer-reviewed, accepted and presented*

Wood, Pamela; Niebuhr, Virginia; Jaffe, David; Livingston, Judith; Henretig, Fred.

- Pediatric Academic Societies Annual Meeting, Baltimore, MD. April 28, 2019.
- Pediatric Academic Societies Annual Meeting. Online. June 1, 2021.
- Pediatric Academic Societies Annual Meeting. Denver, CO, April 49, 2022

Niebuhr, Virginia; Wood, Pamela; Livingston, Judith; Williams, Janet; Szauter, Karen.

- UT System Shine Academy Annual Meeting, online, Feb 25, 2022.

Wood, Pamela; Niebuhr, Virginia; Jaffe, David; Henretig, Fred

- Pediatric Academic Societies Annual Meeting. Washington DC, April 2023

Wood, Pamela; Niebuhr, Virginia; Henretig, Fred; Jaffe, David; Sirotnak, Andrew.

- Pediatric Academic Societies Annual Meeting. Washington DC, April 29, 2023

*Invited*

Wood, Pamela; Niebuhr, Virginia; Jaffe, David; Livingston, Judith; Henretig, Fred.

- Univ. Texas Health Sciences Center – San Antonio / UT Health-San Antonio. San Antonio, TX. November 19, 2019.

Wood, Pamela; Niebuhr, Virginia

- Cincinnati Children’s Hospital/ Univ of Cincinnati School of Medicine Office of Faculty Affairs, Cincinnati, OH Dec. 7, 2023
