# Supplementary material for: A Workshop and Toolkit to Support Late-Career Transitions for Faculty
Source: MedEdPORTAL. 2024 Nov 12;20:11463. doi: 10.15766/mep_2374-8265.11463 (PMC11554777; doi:10.15766/mep_2374-8265.11463)
Supplement: Supplementary file 1 — Packet-Toolkit.docxToolkit Slides.pptxFacilitators Guide.docxWorkshop Evaluation.docx [file mep_2374-8265.11463-s001.zip › B. Toolkit Slides.pptx]

## Slide 1
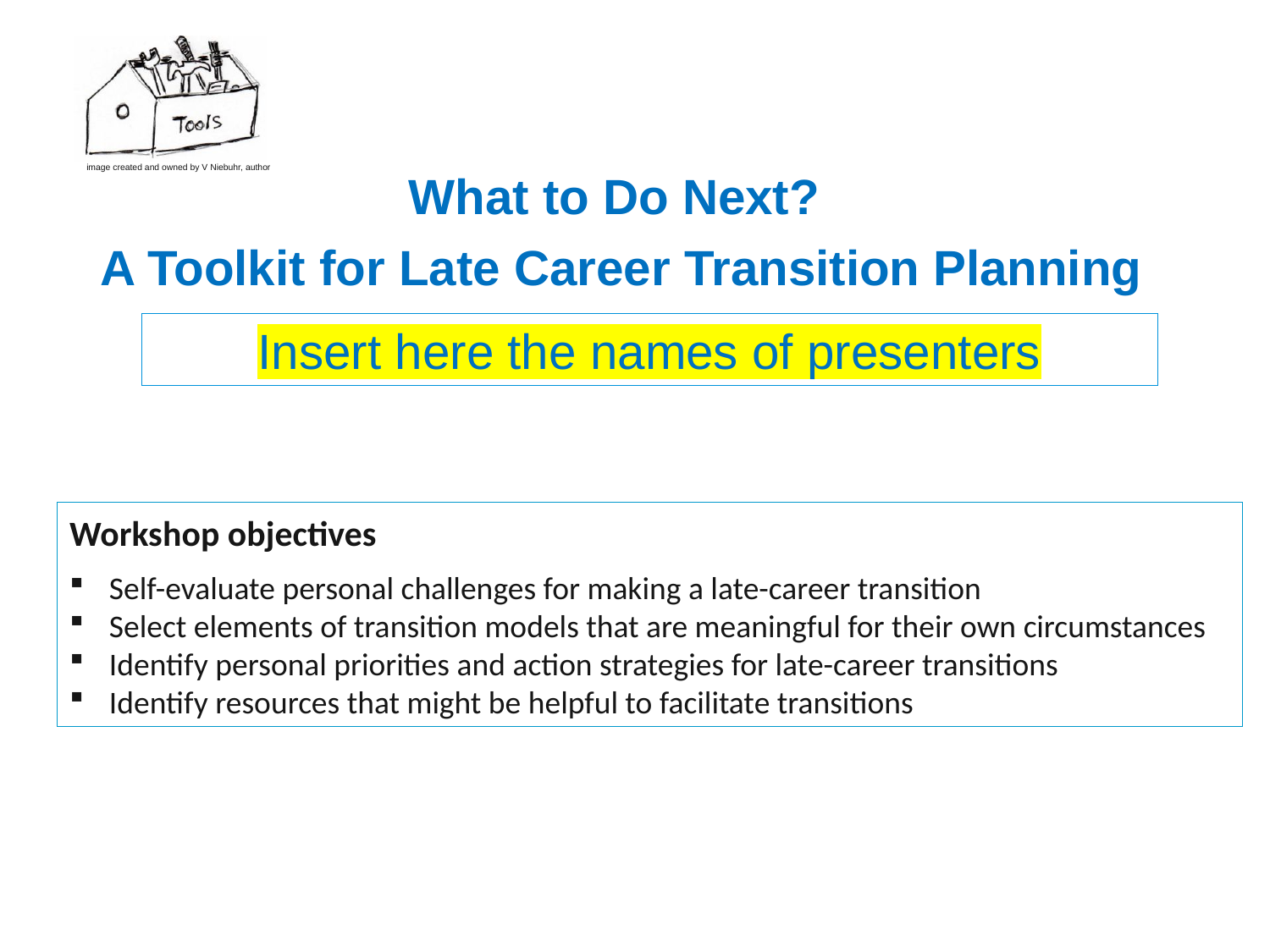

What to Do Next?
A Toolkit for Late Career Transition Planning
image created and owned by V Niebuhr, author
#
Insert here the names of presenters
Workshop objectives
Self-evaluate personal challenges for making a late-career transition
Select elements of transition models that are meaningful for their own circumstances
Identify personal priorities and action strategies for late-career transitions
Identify resources that might be helpful to facilitate transitions

## Slide 2
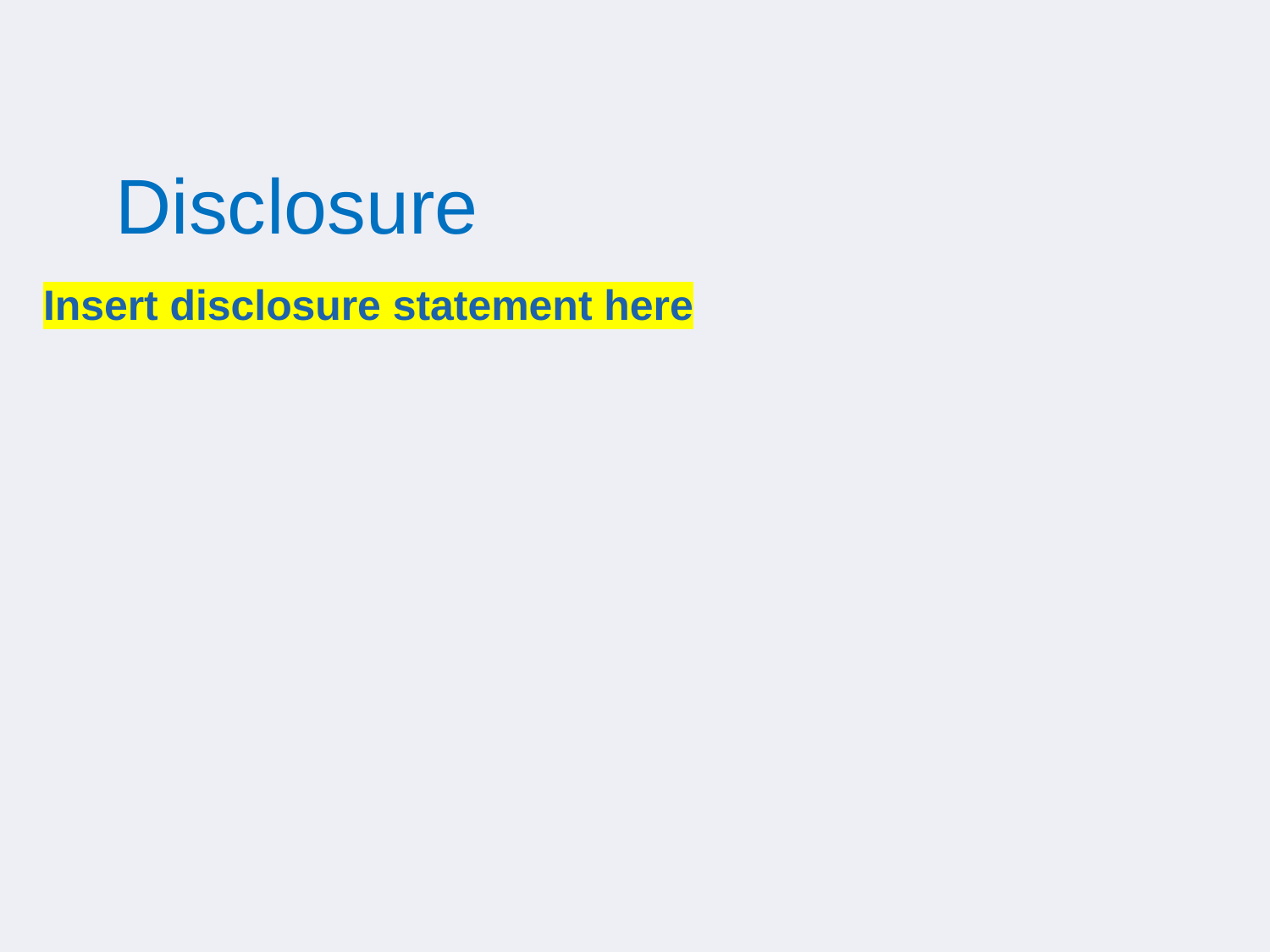

# Disclosure
Insert disclosure statement here

## Slide 3
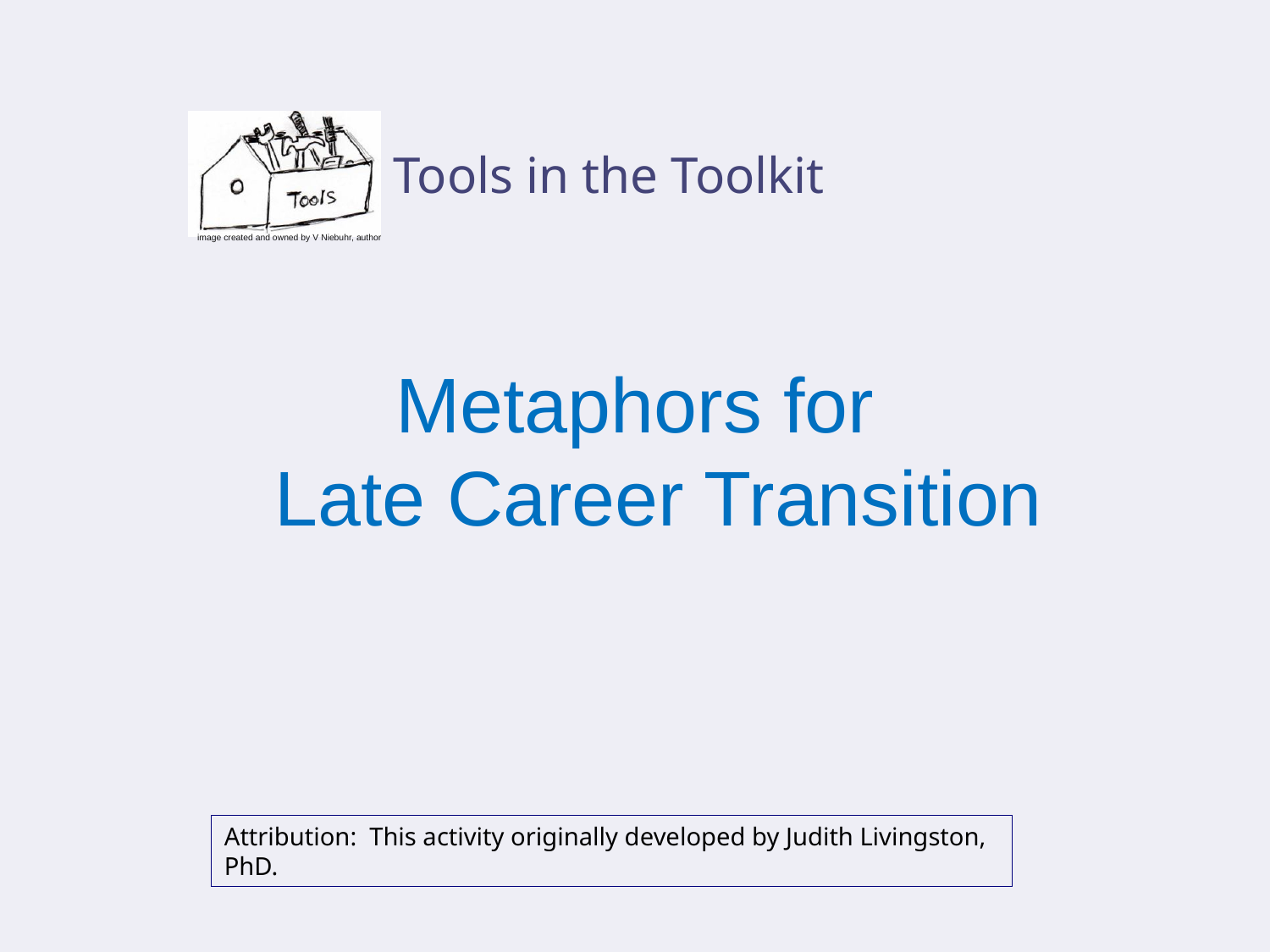

Tools in the Toolkit
image created and owned by V Niebuhr, author
# Metaphors for Late Career Transition
Attribution: This activity originally developed by Judith Livingston, PhD.

## Slide 4
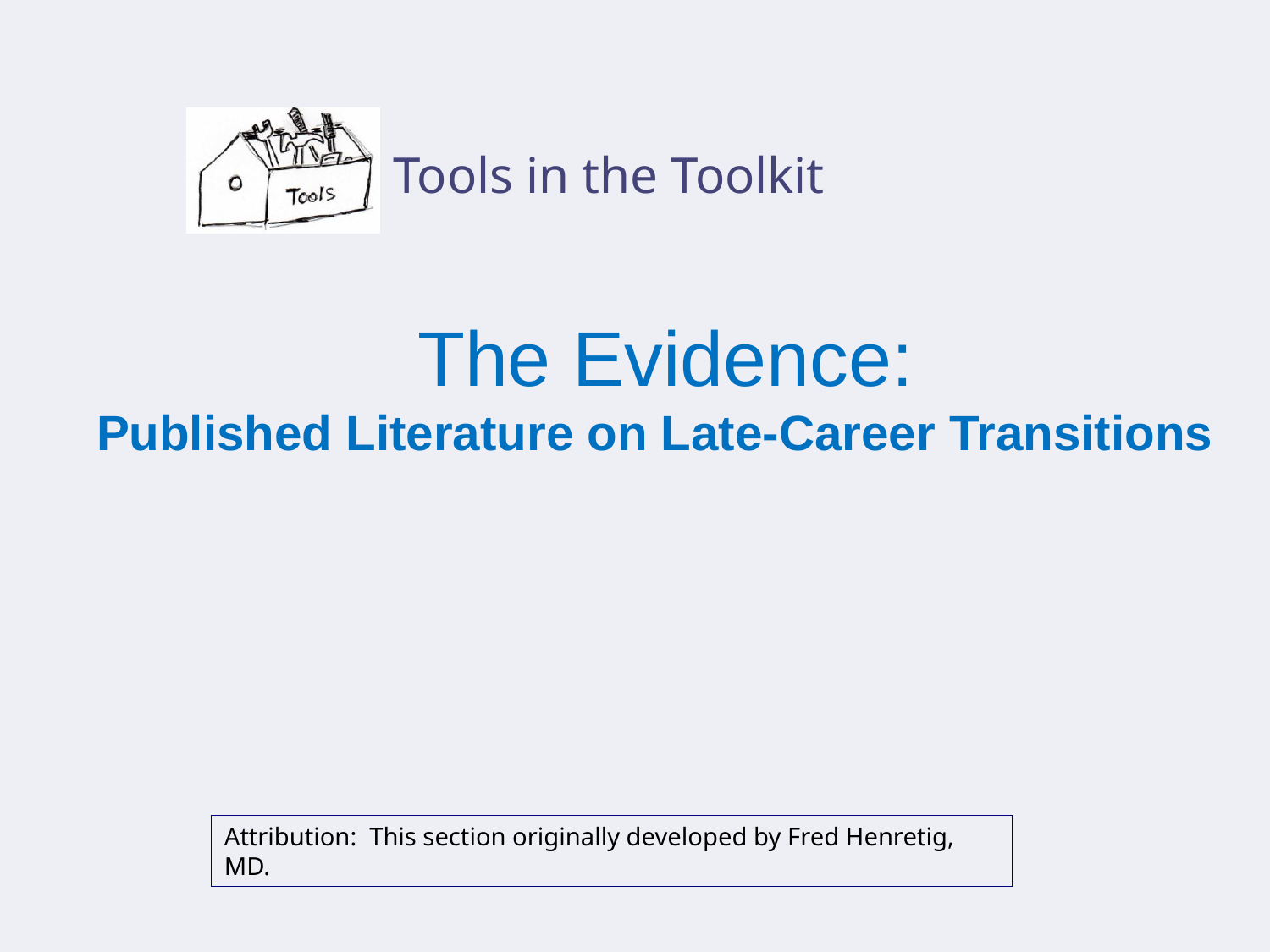

Tools in the Toolkit
# The Evidence: Published Literature on Late-Career Transitions
Attribution: This section originally developed by Fred Henretig, MD.

## Slide 5
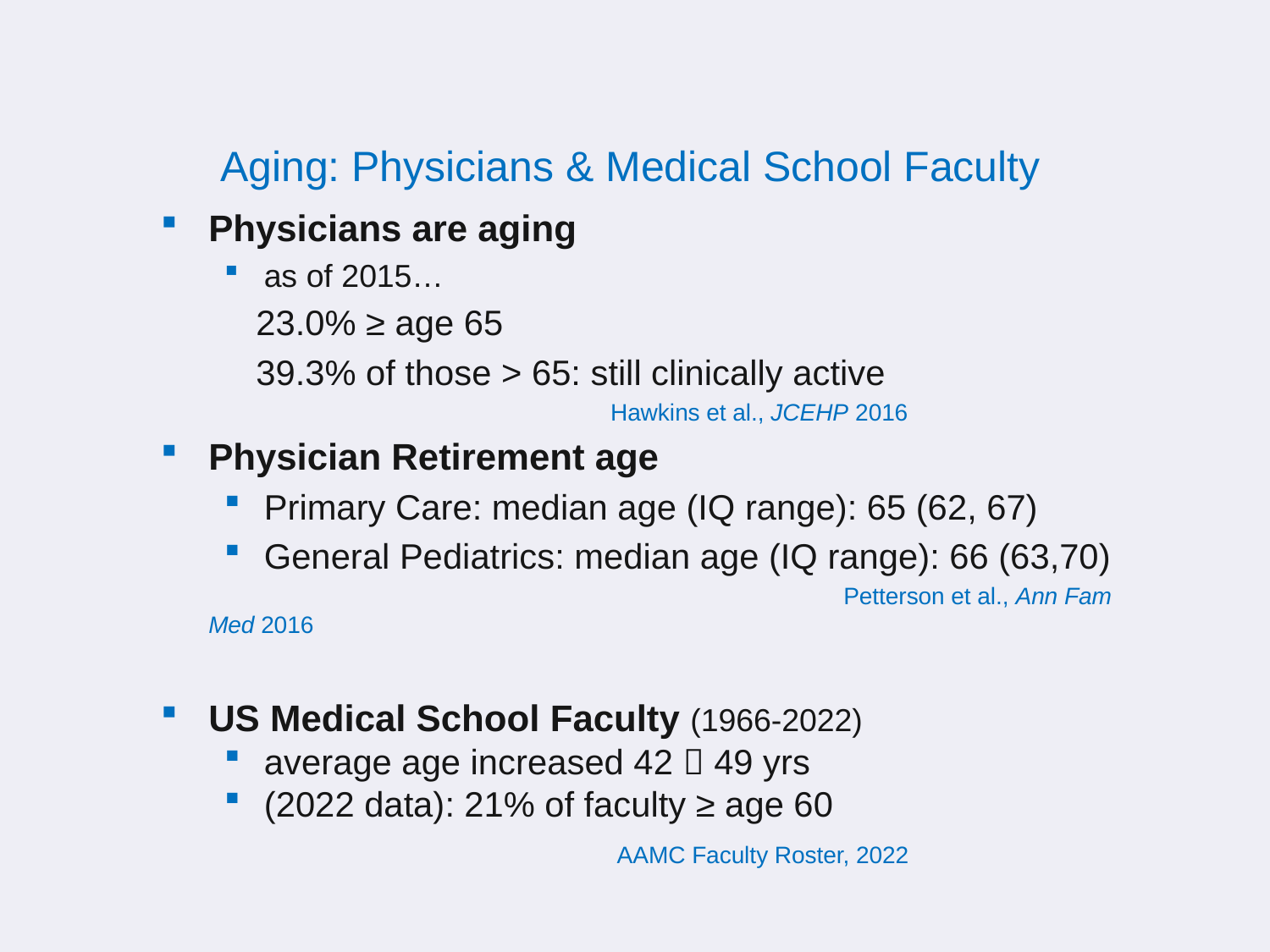

# Aging: Physicians & Medical School Faculty
Physicians are aging
as of 2015…
23.0% ≥ age 65
39.3% of those > 65: still clinically active
 Hawkins et al., JCEHP 2016
Physician Retirement age
Primary Care: median age (IQ range): 65 (62, 67)
General Pediatrics: median age (IQ range): 66 (63,70)
 		Petterson et al., Ann Fam Med 2016
US Medical School Faculty (1966-2022)
average age increased 42  49 yrs
(2022 data): 21% of faculty ≥ age 60
 AAMC Faculty Roster, 2022

## Slide 6
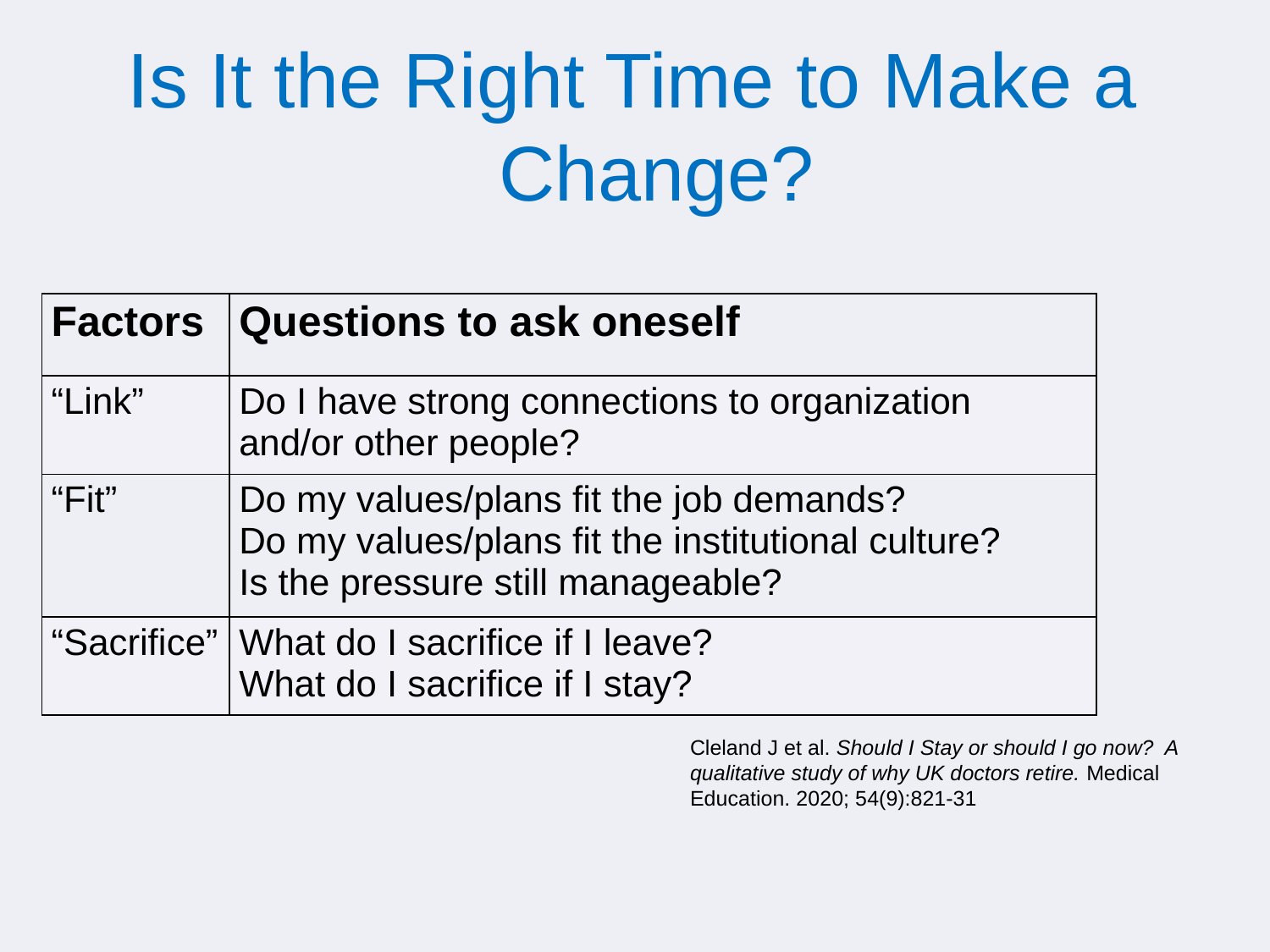

# Is It the Right Time to Make a Change?
| Factors | Questions to ask oneself |
| --- | --- |
| “Link” | Do I have strong connections to organization and/or other people? |
| “Fit” | Do my values/plans fit the job demands? Do my values/plans fit the institutional culture? Is the pressure still manageable? |
| “Sacrifice” | What do I sacrifice if I leave? What do I sacrifice if I stay? |
Cleland J et al. Should I Stay or should I go now? A qualitative study of why UK doctors retire. Medical Education. 2020; 54(9):821-31

## Slide 7
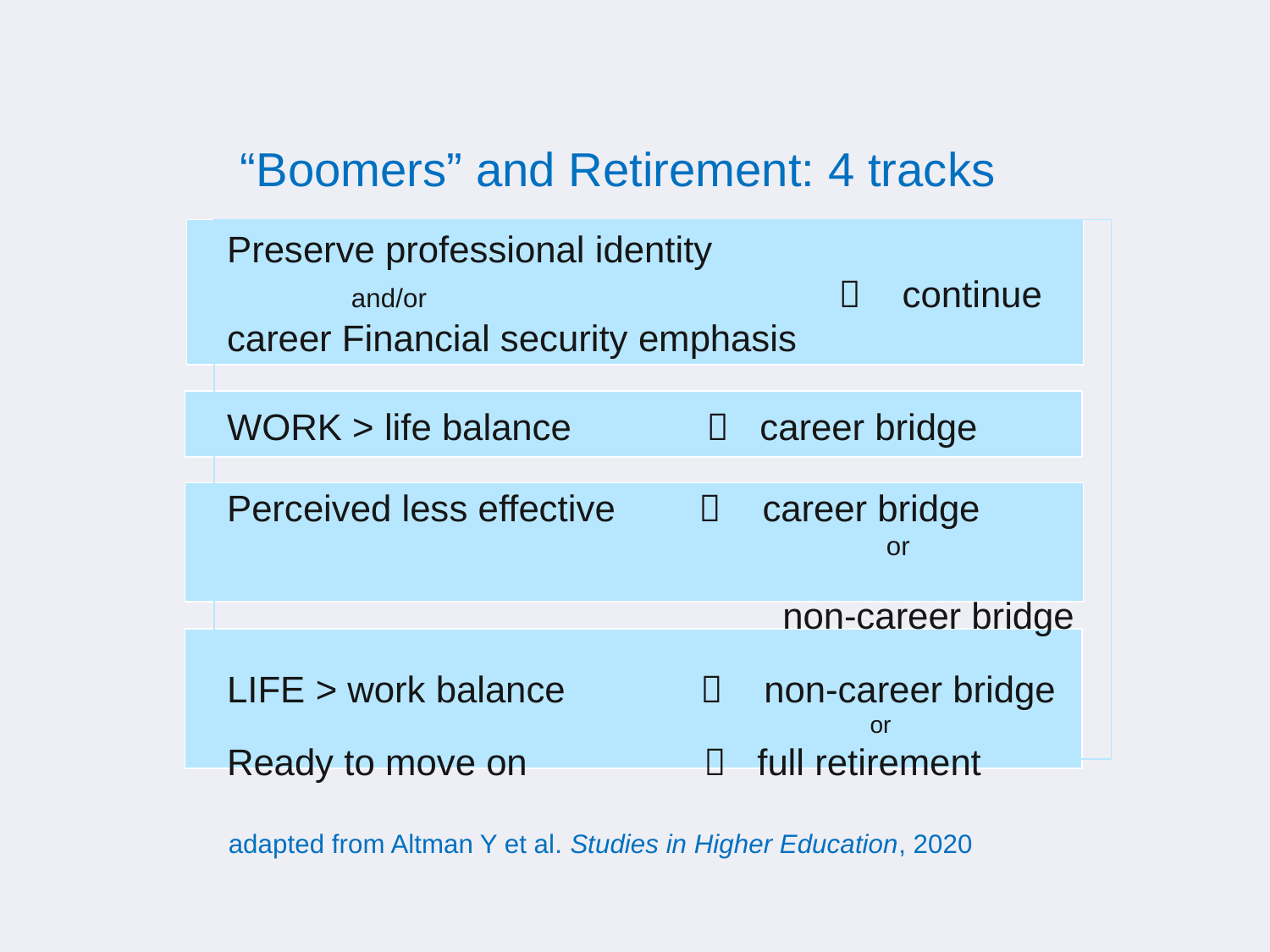

# “Boomers” and Retirement: 4 tracks
Preserve professional identity
 and/or 		  continue career Financial security emphasis
WORK > life balance  career bridge
Perceived less effective  career bridge
 or
 non-career bridge
LIFE > work balance  non-career bridge
 or
Ready to move on  full retirement
adapted from Altman Y et al. Studies in Higher Education, 2020

## Slide 8
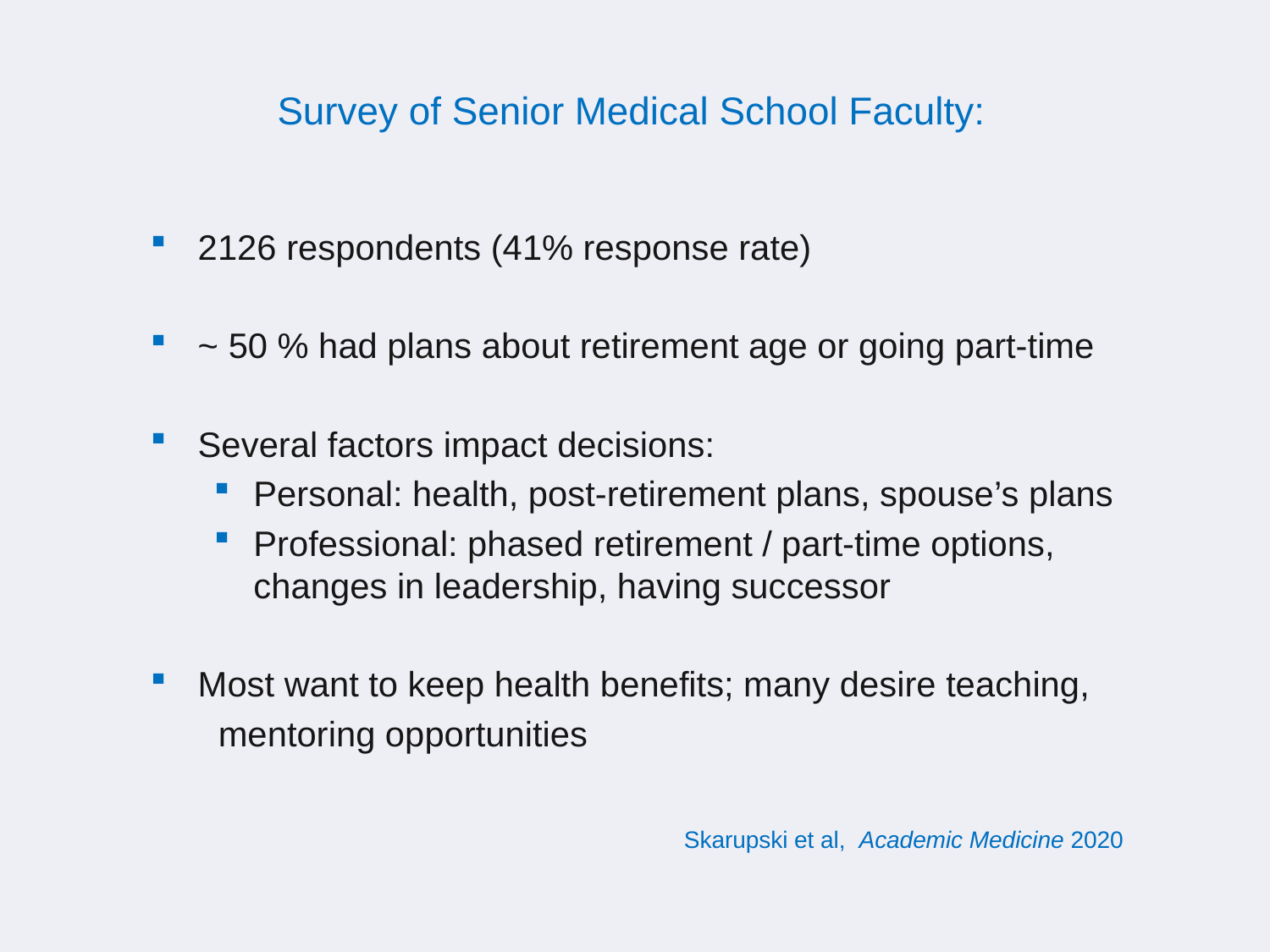

# Survey of Senior Medical School Faculty:
2126 respondents (41% response rate)
~ 50 % had plans about retirement age or going part-time
Several factors impact decisions:
Personal: health, post-retirement plans, spouse’s plans
Professional: phased retirement / part-time options, changes in leadership, having successor
Most want to keep health benefits; many desire teaching,
 mentoring opportunities
	 Skarupski et al, Academic Medicine 2020

## Slide 9
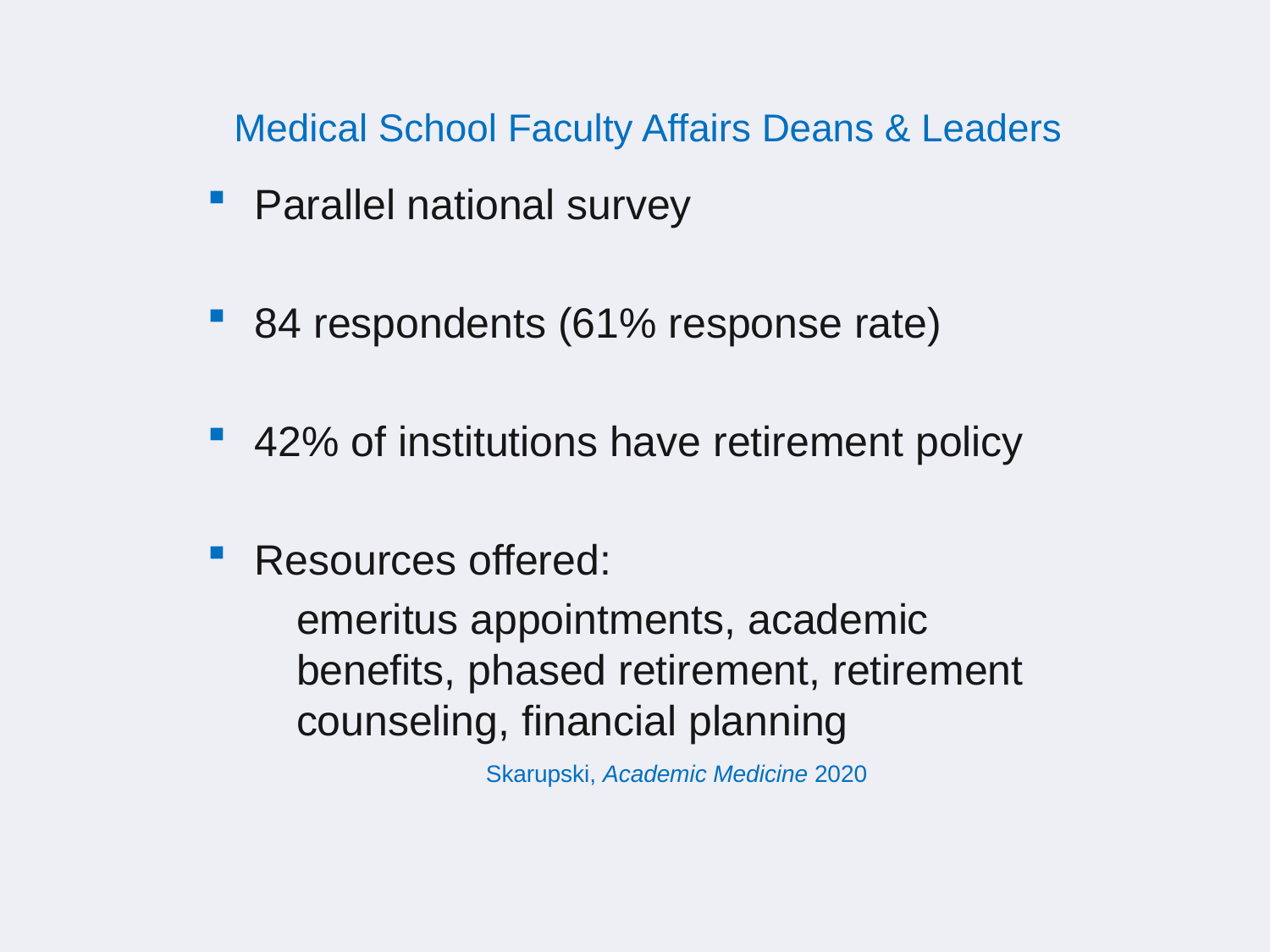

# Medical School Faculty Affairs Deans & Leaders
Parallel national survey
84 respondents (61% response rate)
42% of institutions have retirement policy
Resources offered:
emeritus appointments, academic benefits, phased retirement, retirement counseling, financial planning
 Skarupski, Academic Medicine 2020

## Slide 10
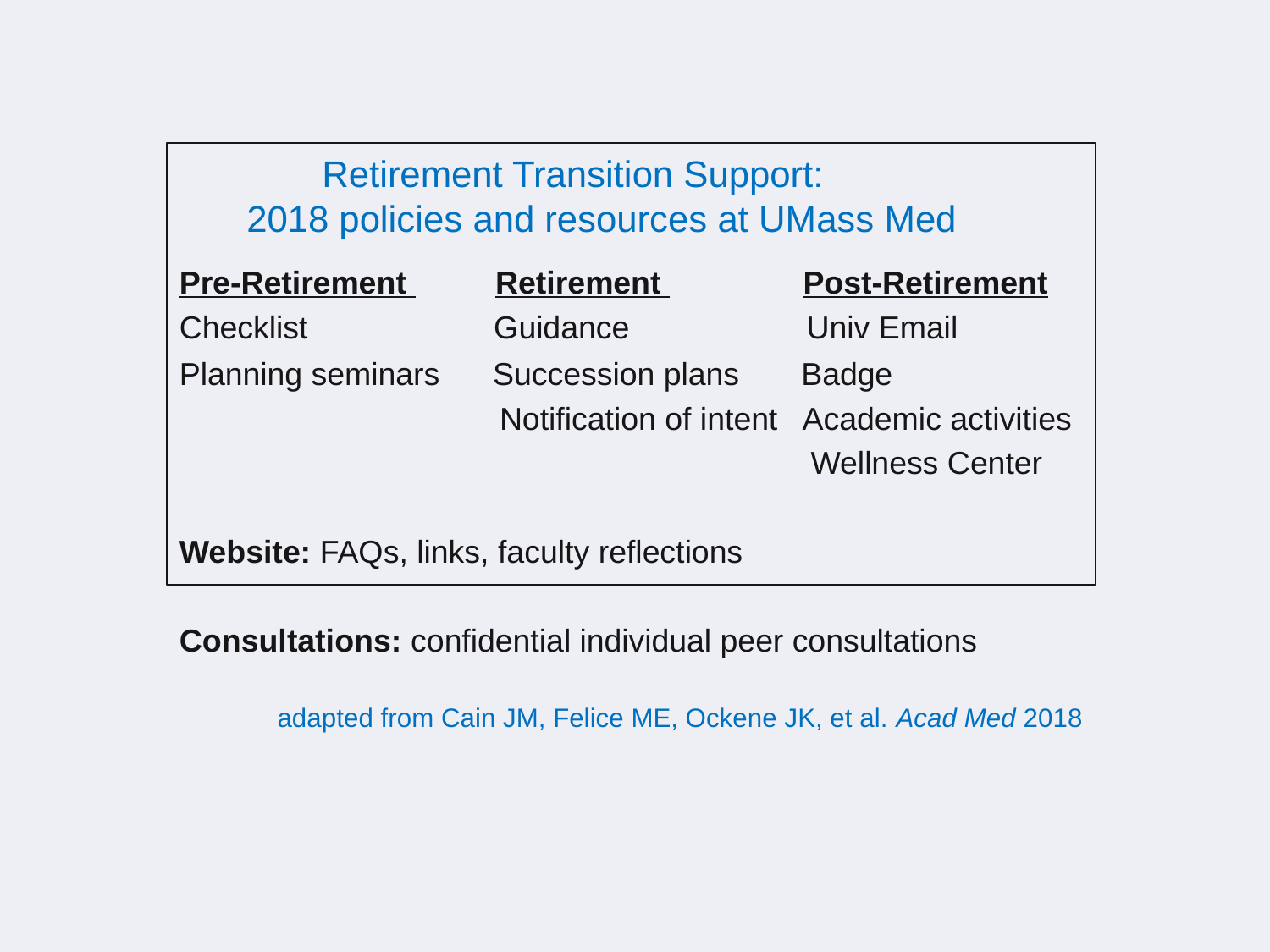

# Retirement Transition Support: 2018 policies and resources at UMass Med
Pre-Retirement Retirement Post-Retirement
Checklist Guidance Univ Email
Planning seminars Succession plans Badge
 Notification of intent Academic activities
 Wellness Center
Website: FAQs, links, faculty reflections
Consultations: confidential individual peer consultations
adapted from Cain JM, Felice ME, Ockene JK, et al. Acad Med 2018

## Slide 11
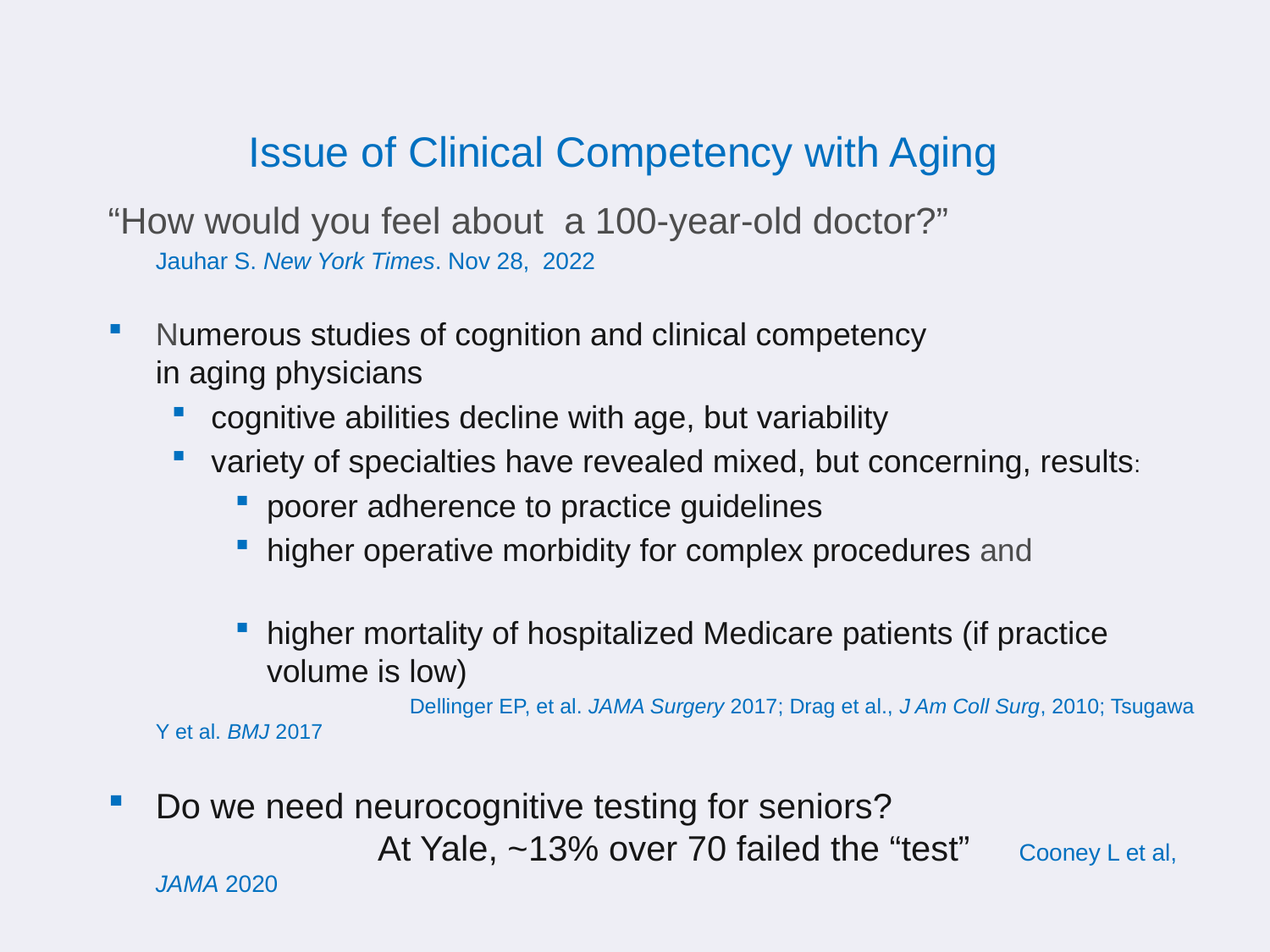

# Issue of Clinical Competency with Aging
“How would you feel about a 100-year-old doctor?”
Jauhar S. New York Times. Nov 28, 2022
Numerous studies of cognition and clinical competency
in aging physicians
cognitive abilities decline with age, but variability
variety of specialties have revealed mixed, but concerning, results:
poorer adherence to practice guidelines
higher operative morbidity for complex procedures and
higher mortality of hospitalized Medicare patients (if practice volume is low)
		Dellinger EP, et al. JAMA Surgery 2017; Drag et al., J Am Coll Surg, 2010; Tsugawa Y et al. BMJ 2017
Do we need neurocognitive testing for seniors? 	 At Yale, ~13% over 70 failed the “test” Cooney L et al, JAMA 2020

## Slide 12
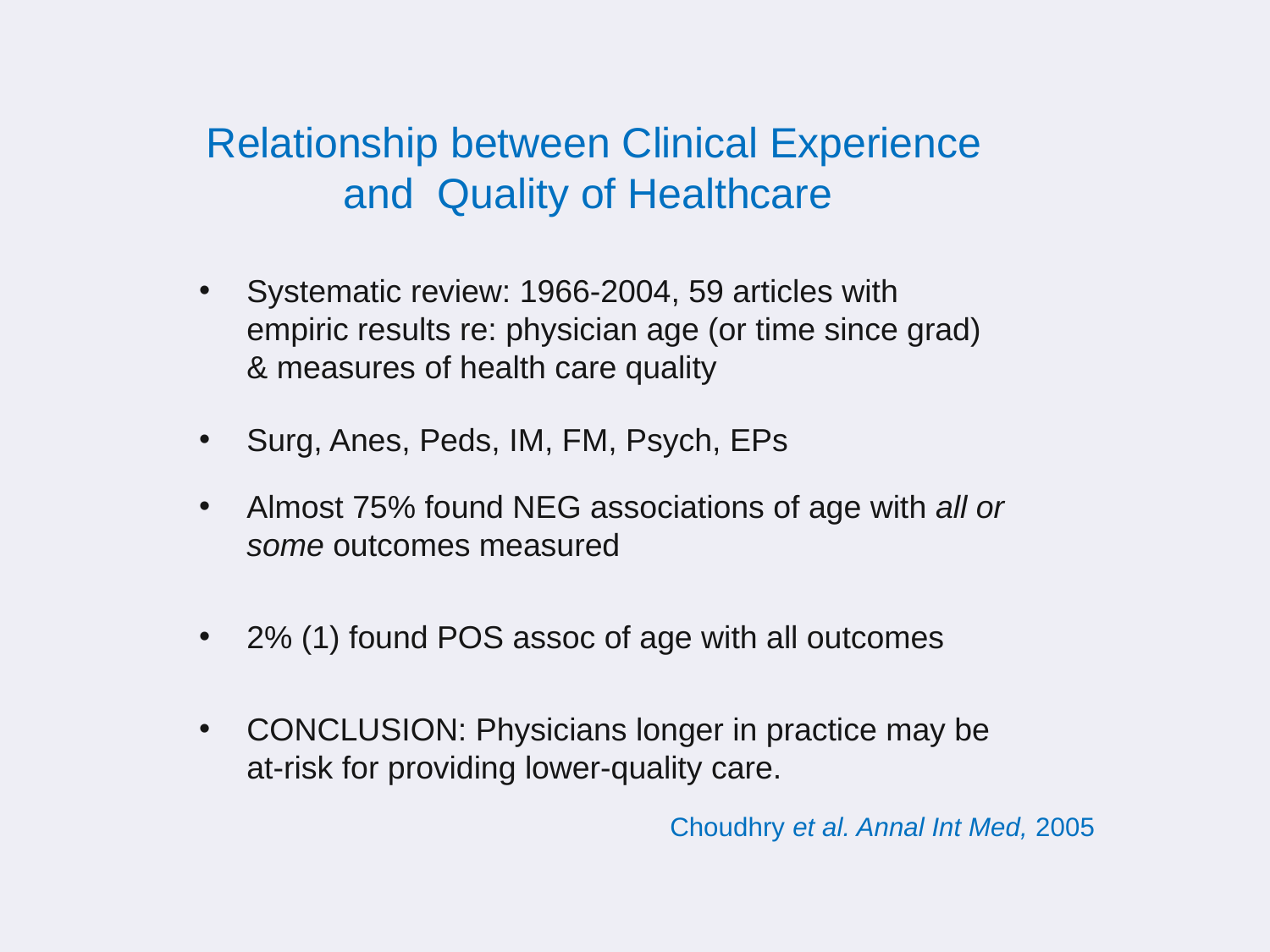

# Relationship between Clinical Experience and Quality of Healthcare
Systematic review: 1966-2004, 59 articles with empiric results re: physician age (or time since grad) & measures of health care quality
Surg, Anes, Peds, IM, FM, Psych, EPs
Almost 75% found NEG associations of age with all or some outcomes measured
2% (1) found POS assoc of age with all outcomes
CONCLUSION: Physicians longer in practice may be at-risk for providing lower-quality care.
Choudhry et al. Annal Int Med, 2005

## Slide 13
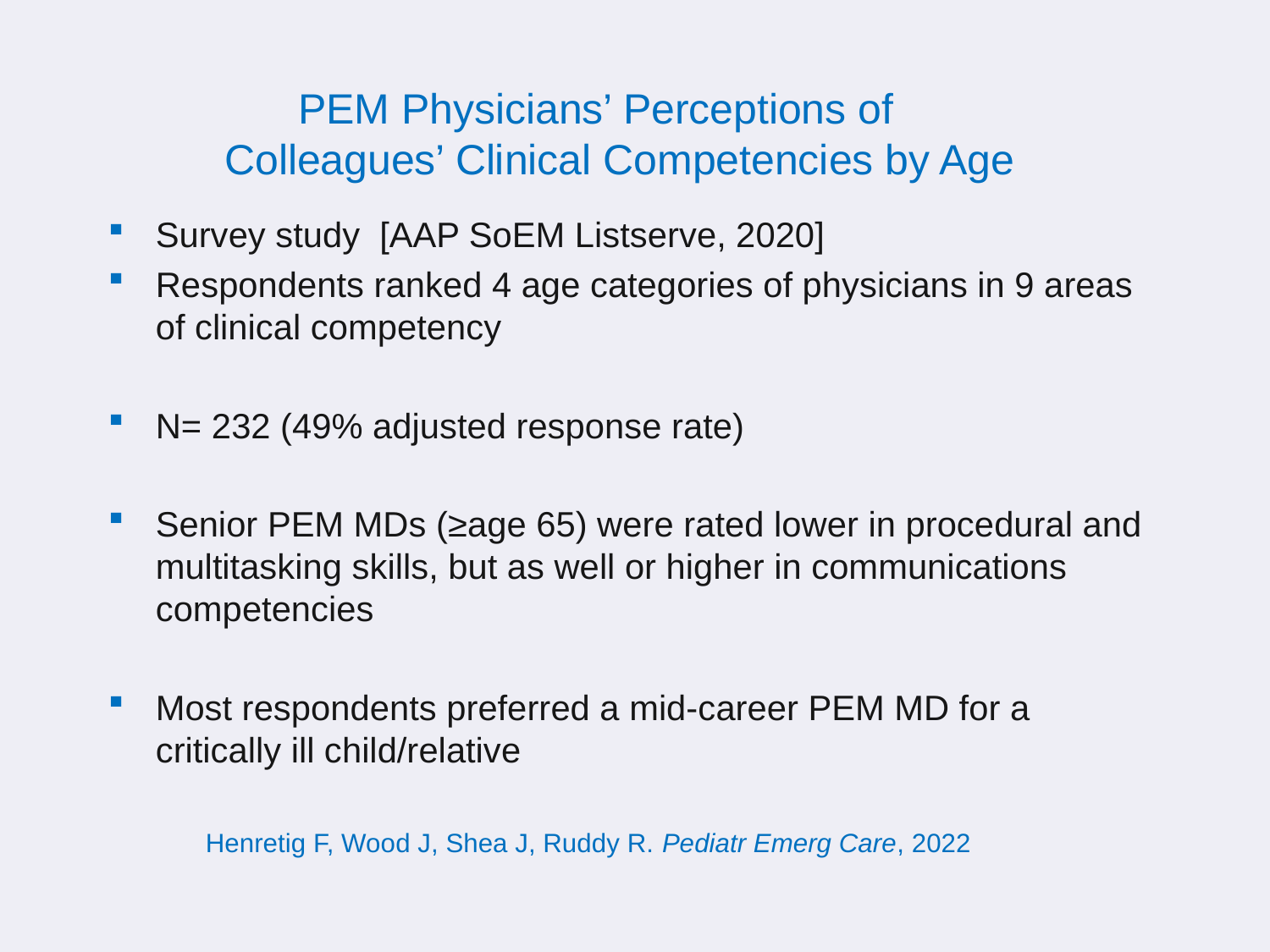

# PEM Physicians’ Perceptions of Colleagues’ Clinical Competencies by Age
Survey study [AAP SoEM Listserve, 2020]
Respondents ranked 4 age categories of physicians in 9 areas of clinical competency
N= 232 (49% adjusted response rate)
Senior PEM MDs (≥age 65) were rated lower in procedural and multitasking skills, but as well or higher in communications competencies
Most respondents preferred a mid-career PEM MD for a critically ill child/relative
 Henretig F, Wood J, Shea J, Ruddy R. Pediatr Emerg Care, 2022

## Slide 14
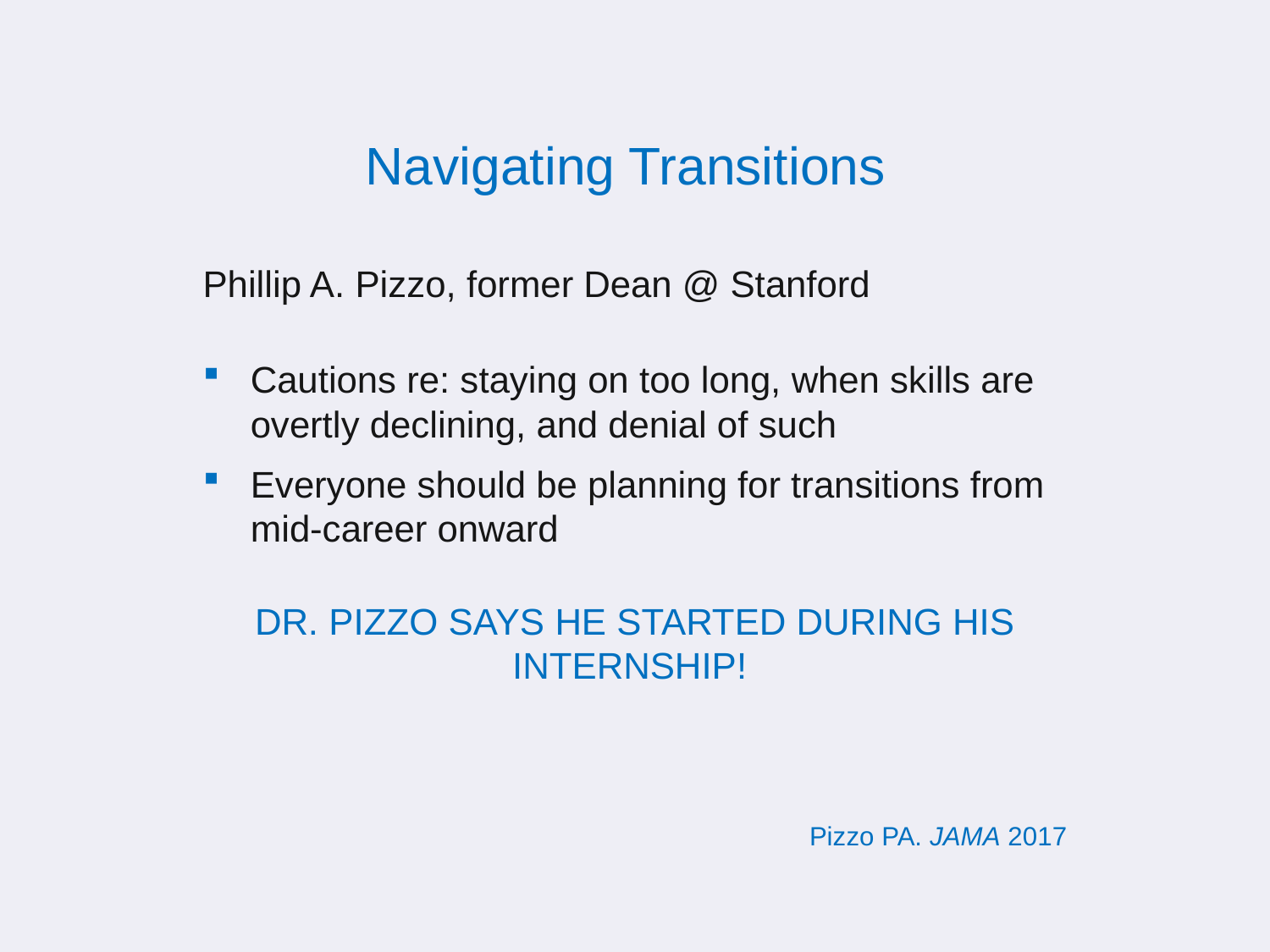

# Navigating Transitions
Phillip A. Pizzo, former Dean @ Stanford
Cautions re: staying on too long, when skills are overtly declining, and denial of such
Everyone should be planning for transitions from mid-career onward
Dr. Pizzo says he started during his internship!
Pizzo PA. JAMA 2017

## Slide 15
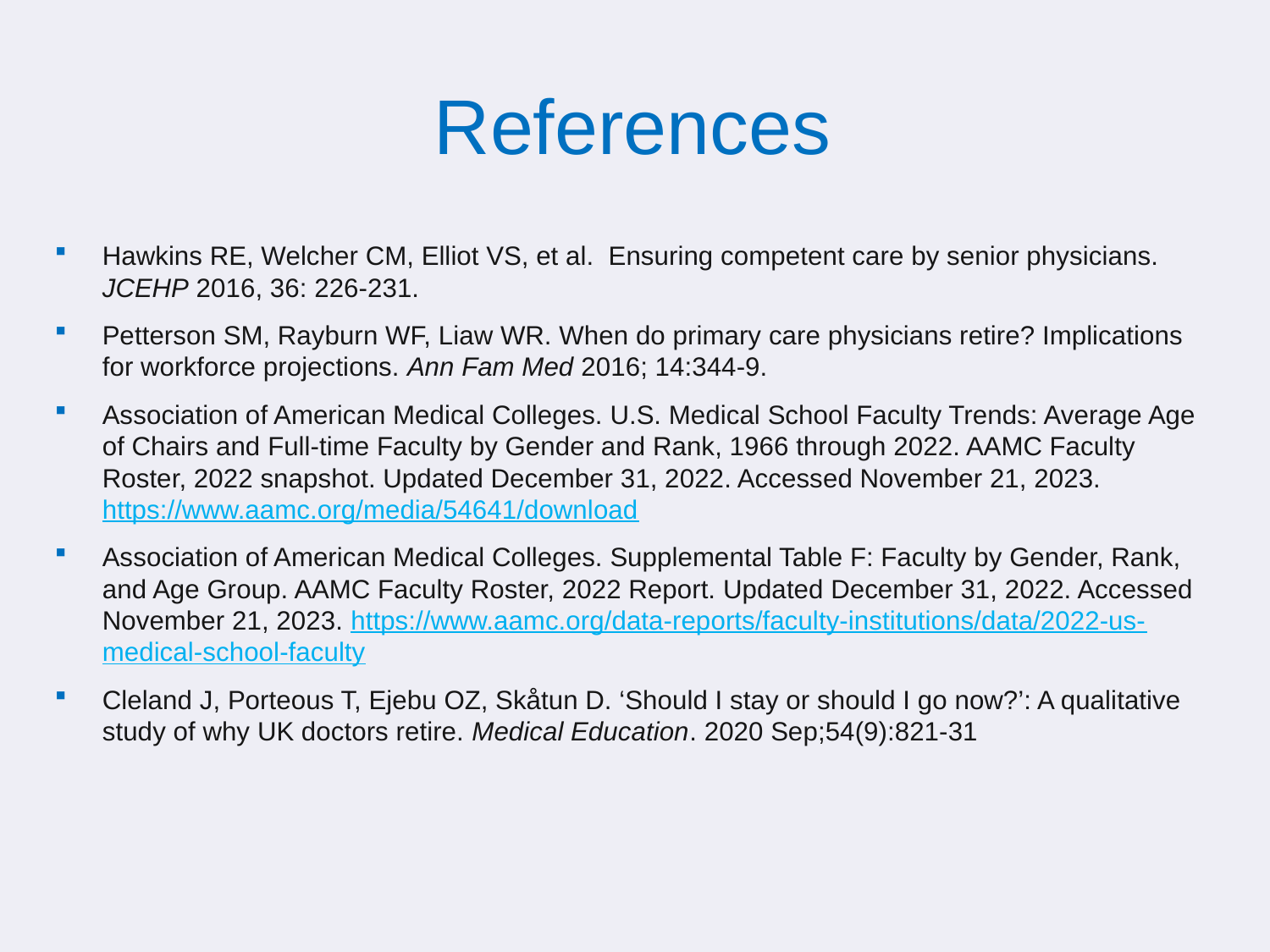

# References
Hawkins RE, Welcher CM, Elliot VS, et al. Ensuring competent care by senior physicians. JCEHP 2016, 36: 226-231.
Petterson SM, Rayburn WF, Liaw WR. When do primary care physicians retire? Implications for workforce projections. Ann Fam Med 2016; 14:344-9.
Association of American Medical Colleges. U.S. Medical School Faculty Trends: Average Age of Chairs and Full-time Faculty by Gender and Rank, 1966 through 2022. AAMC Faculty Roster, 2022 snapshot. Updated December 31, 2022. Accessed November 21, 2023. https://www.aamc.org/media/54641/download
Association of American Medical Colleges. Supplemental Table F: Faculty by Gender, Rank, and Age Group. AAMC Faculty Roster, 2022 Report. Updated December 31, 2022. Accessed November 21, 2023. https://www.aamc.org/data-reports/faculty-institutions/data/2022-us-medical-school-faculty
Cleland J, Porteous T, Ejebu OZ, Skåtun D. ‘Should I stay or should I go now?’: A qualitative study of why UK doctors retire. Medical Education. 2020 Sep;54(9):821-31

## Slide 16
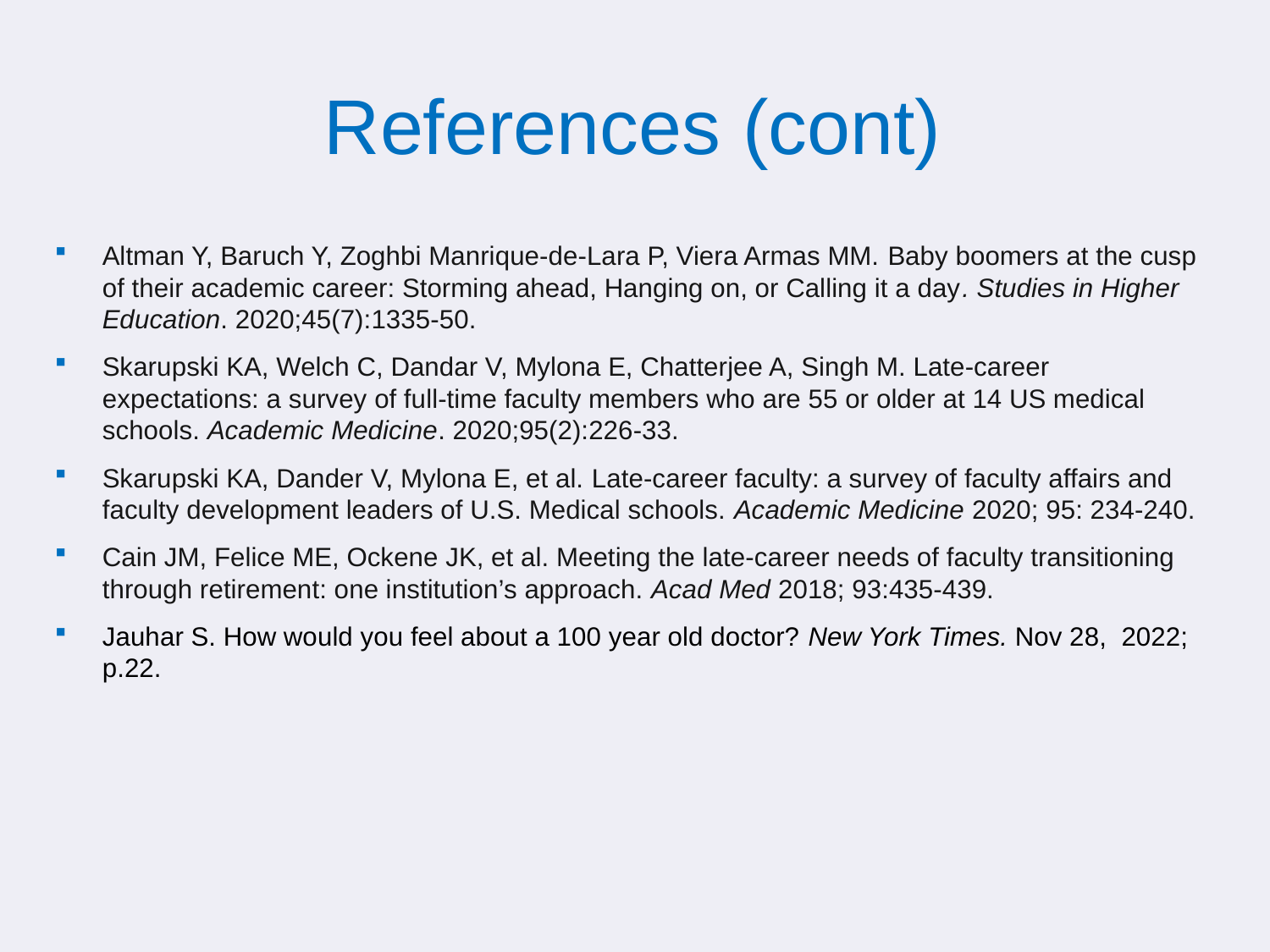

# References (cont)
Altman Y, Baruch Y, Zoghbi Manrique-de-Lara P, Viera Armas MM. Baby boomers at the cusp of their academic career: Storming ahead, Hanging on, or Calling it a day. Studies in Higher Education. 2020;45(7):1335-50.
Skarupski KA, Welch C, Dandar V, Mylona E, Chatterjee A, Singh M. Late-career expectations: a survey of full-time faculty members who are 55 or older at 14 US medical schools. Academic Medicine. 2020;95(2):226-33.
Skarupski KA, Dander V, Mylona E, et al. Late-career faculty: a survey of faculty affairs and faculty development leaders of U.S. Medical schools. Academic Medicine 2020; 95: 234-240.
Cain JM, Felice ME, Ockene JK, et al. Meeting the late-career needs of faculty transitioning through retirement: one institution’s approach. Acad Med 2018; 93:435-439.
Jauhar S. How would you feel about a 100 year old doctor? New York Times. Nov 28, 2022; p.22.

## Slide 17
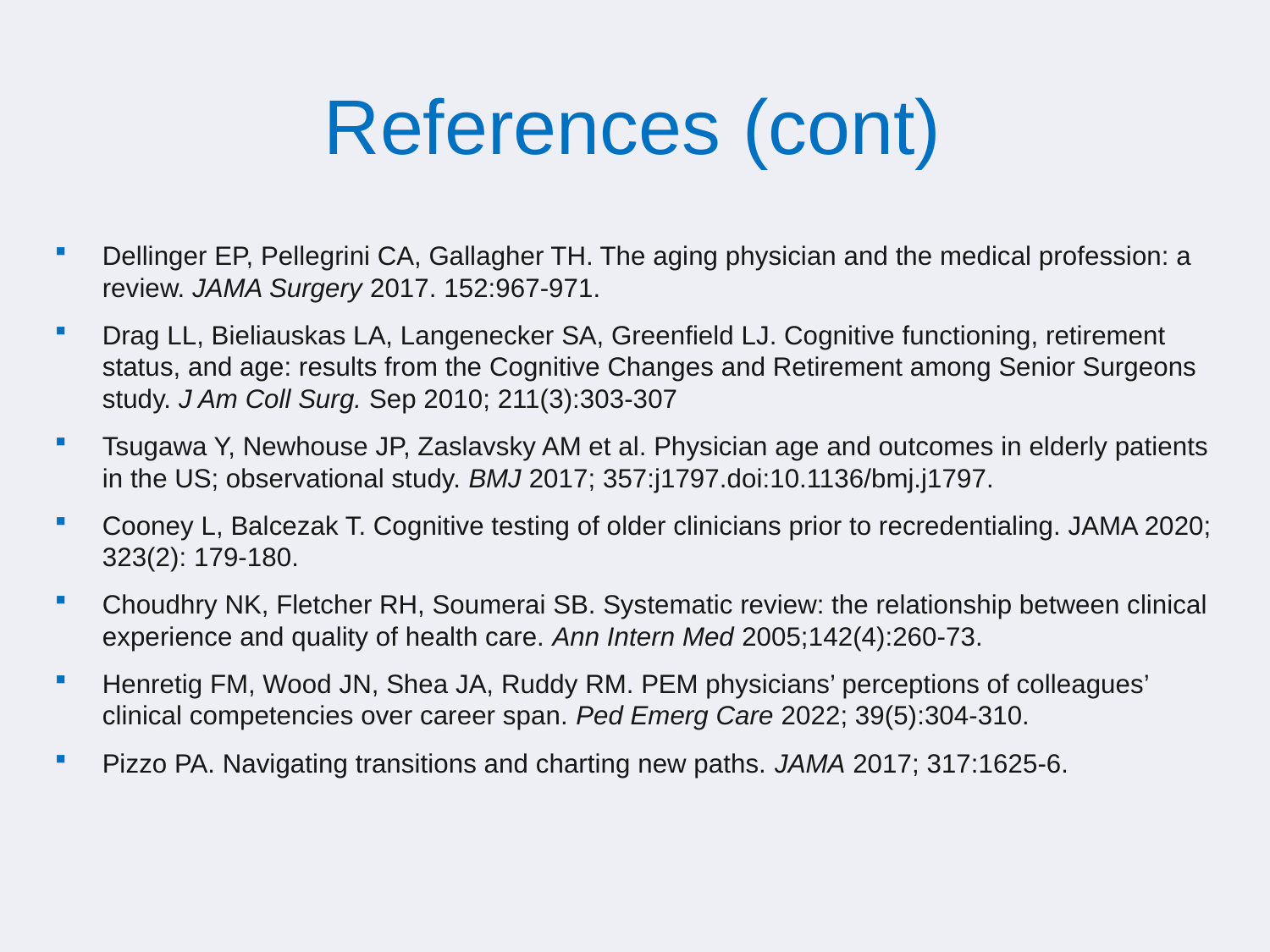

# References (cont)
Dellinger EP, Pellegrini CA, Gallagher TH. The aging physician and the medical profession: a review. JAMA Surgery 2017. 152:967-971.
Drag LL, Bieliauskas LA, Langenecker SA, Greenfield LJ. Cognitive functioning, retirement status, and age: results from the Cognitive Changes and Retirement among Senior Surgeons study. J Am Coll Surg. Sep 2010; 211(3):303-307
Tsugawa Y, Newhouse JP, Zaslavsky AM et al. Physician age and outcomes in elderly patients in the US; observational study. BMJ 2017; 357:j1797.doi:10.1136/bmj.j1797.
Cooney L, Balcezak T. Cognitive testing of older clinicians prior to recredentialing. JAMA 2020; 323(2): 179-180.
Choudhry NK, Fletcher RH, Soumerai SB. Systematic review: the relationship between clinical experience and quality of health care. Ann Intern Med 2005;142(4):260-73.
Henretig FM, Wood JN, Shea JA, Ruddy RM. PEM physicians’ perceptions of colleagues’ clinical competencies over career span. Ped Emerg Care 2022; 39(5):304-310.
Pizzo PA. Navigating transitions and charting new paths. JAMA 2017; 317:1625-6.

## Slide 18
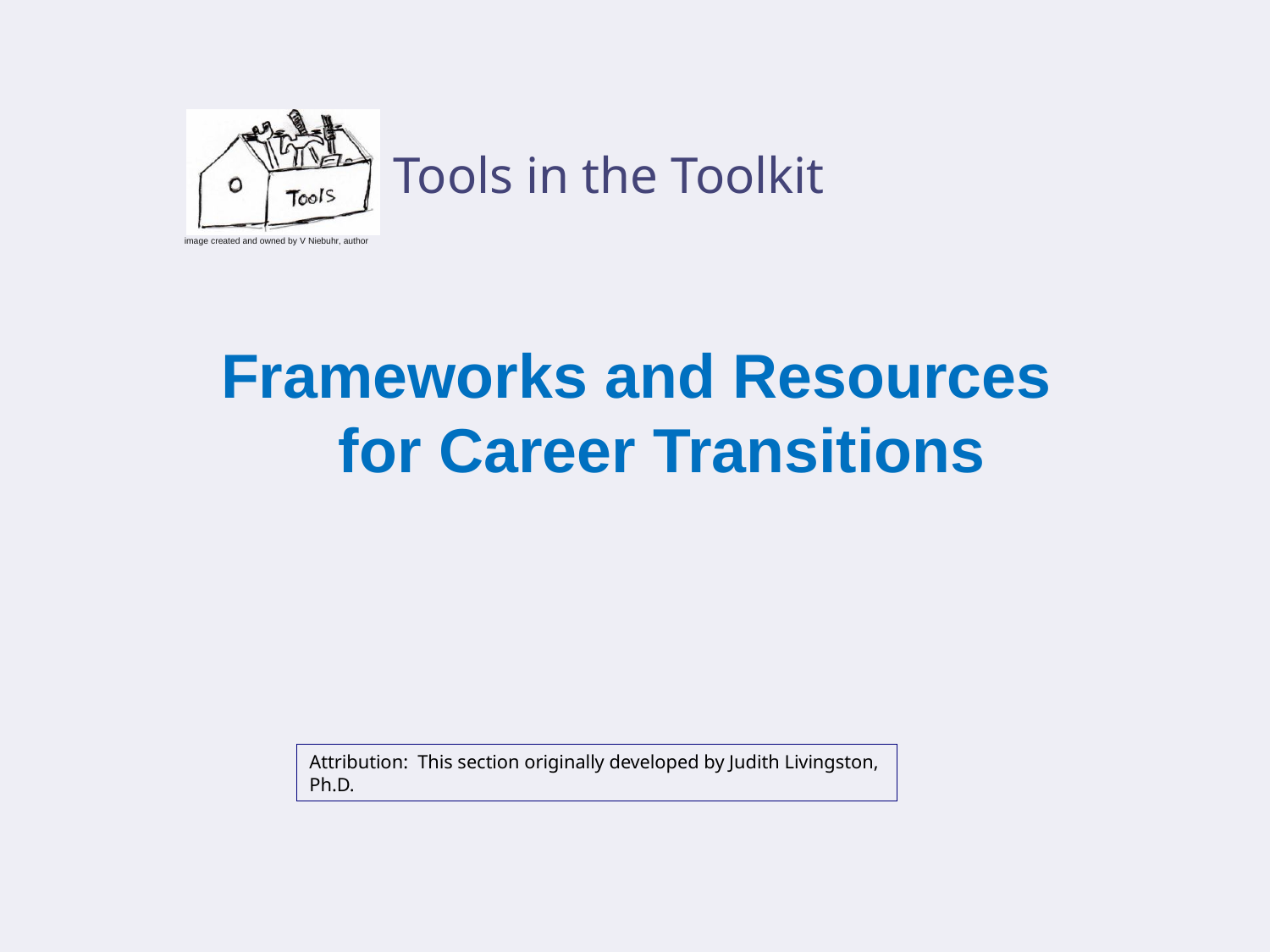

Tools in the Toolkit
image created and owned by V Niebuhr, author
# Frameworks and Resources for Career Transitions
Attribution: This section originally developed by Judith Livingston, Ph.D.

## Slide 19
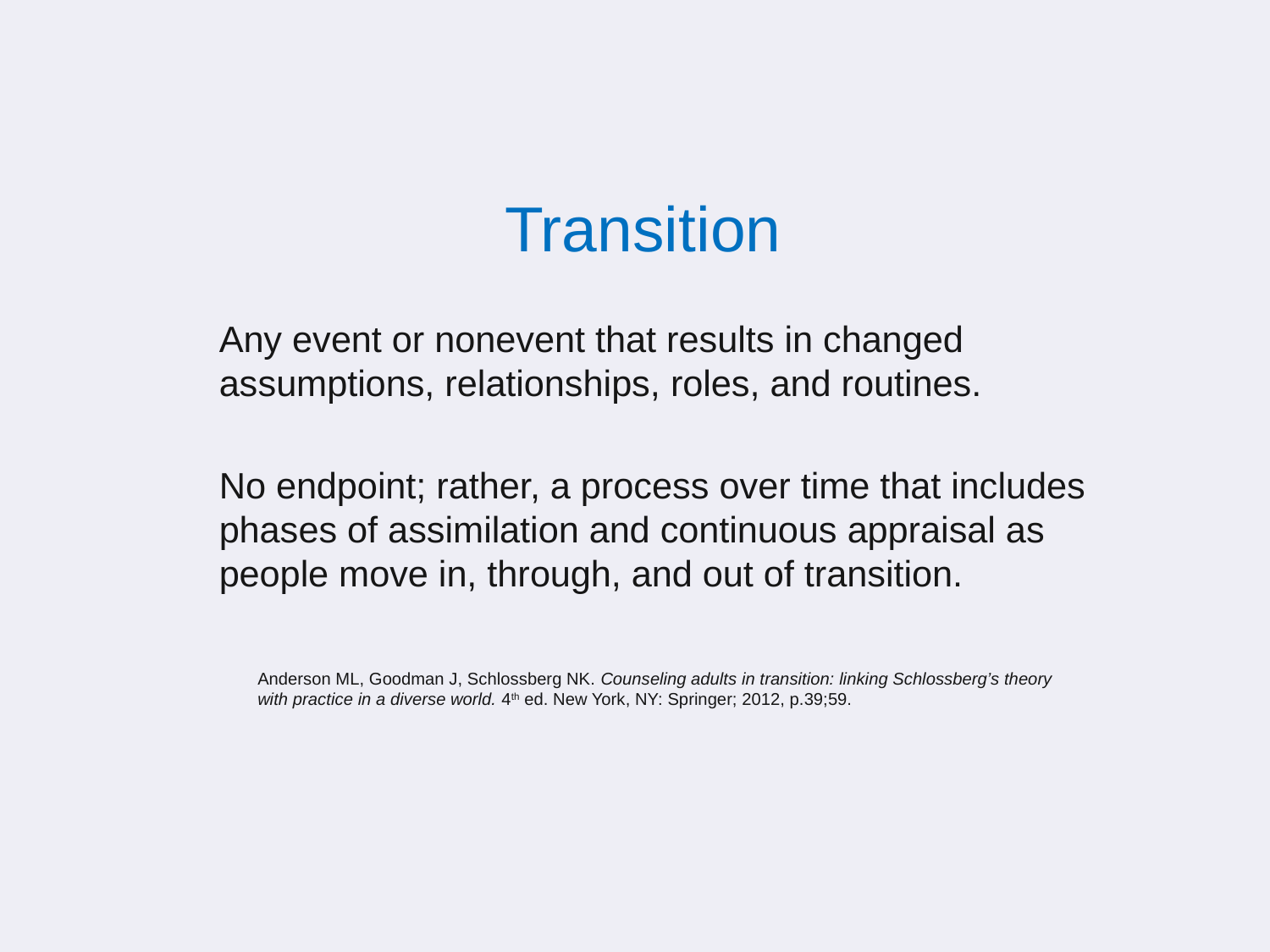

# Transition
Any event or nonevent that results in changed assumptions, relationships, roles, and routines.
No endpoint; rather, a process over time that includes phases of assimilation and continuous appraisal as people move in, through, and out of transition.
Anderson ML, Goodman J, Schlossberg NK. Counseling adults in transition: linking Schlossberg’s theory with practice in a diverse world. 4th ed. New York, NY: Springer; 2012, p.39;59.

## Slide 20
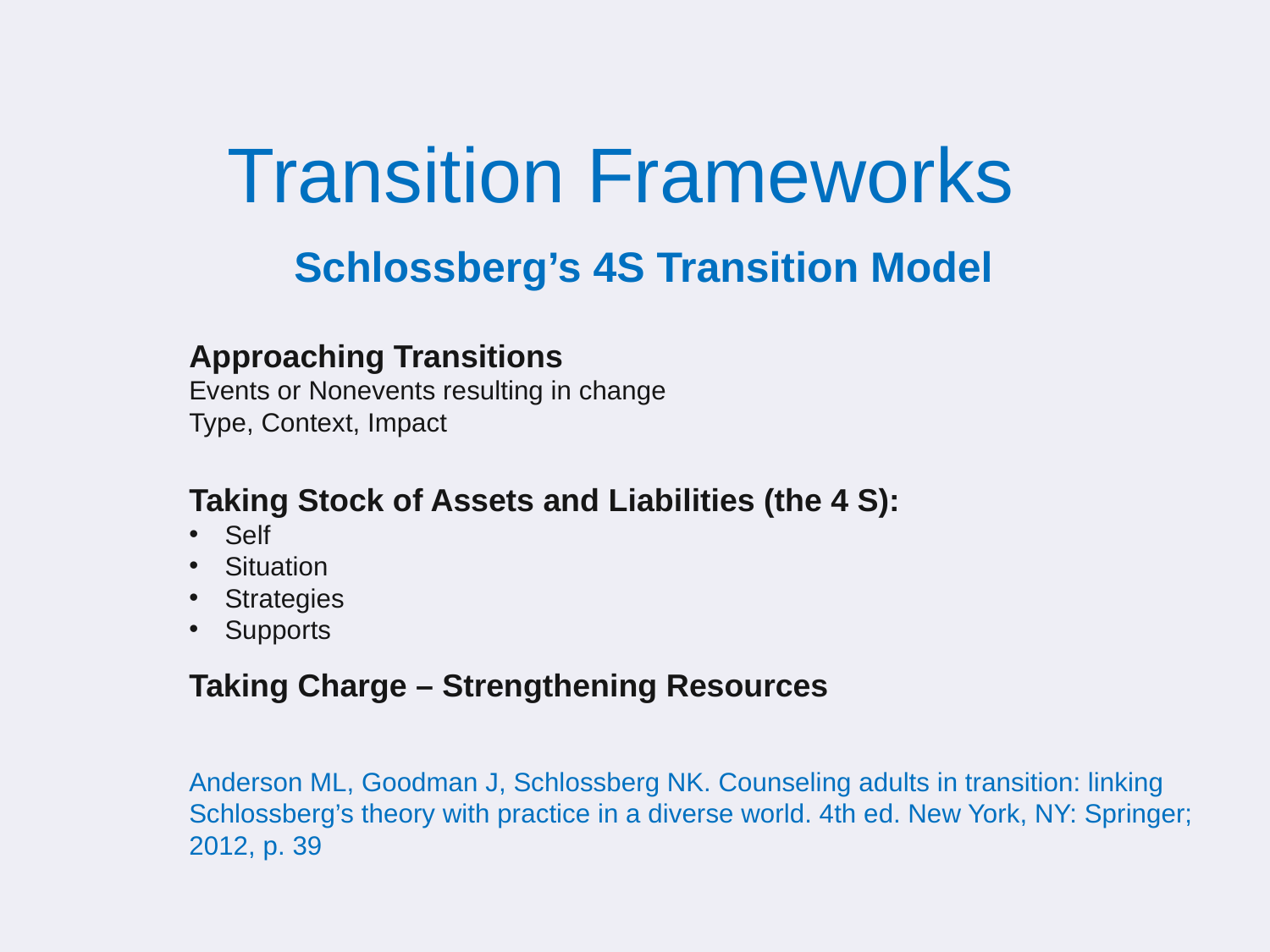

# Transition Frameworks
Schlossberg’s 4S Transition Model
Approaching Transitions
Events or Nonevents resulting in change
Type, Context, Impact
Taking Stock of Assets and Liabilities (the 4 S):
Self
Situation
Strategies
Supports
Taking Charge – Strengthening Resources
Anderson ML, Goodman J, Schlossberg NK. Counseling adults in transition: linking Schlossberg’s theory with practice in a diverse world. 4th ed. New York, NY: Springer; 2012, p. 39

## Slide 21
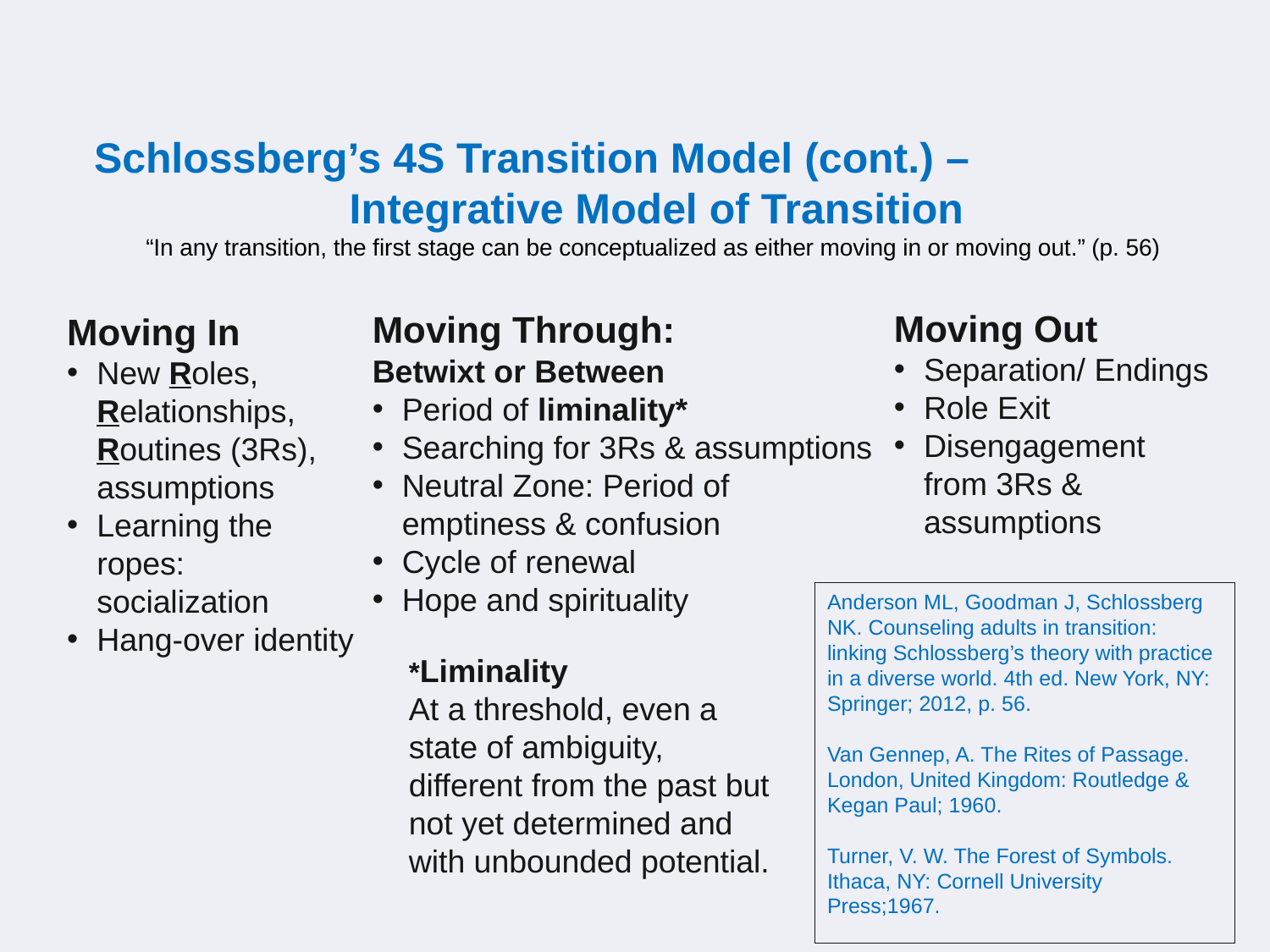

# Schlossberg’s 4S Transition Model (cont.) – Integrative Model of Transition “In any transition, the first stage can be conceptualized as either moving in or moving out.” (p. 56)
Moving Out
Separation/ Endings
Role Exit
Disengagement from 3Rs & assumptions
Moving Through:
Betwixt or Between
Period of liminality*
Searching for 3Rs & assumptions
Neutral Zone: Period of emptiness & confusion
Cycle of renewal
Hope and spirituality
Moving In
New Roles, Relationships, Routines (3Rs), assumptions
Learning the ropes: socialization
Hang-over identity
Anderson ML, Goodman J, Schlossberg NK. Counseling adults in transition: linking Schlossberg’s theory with practice in a diverse world. 4th ed. New York, NY: Springer; 2012, p. 56.
Van Gennep, A. The Rites of Passage. London, United Kingdom: Routledge & Kegan Paul; 1960.
Turner, V. W. The Forest of Symbols. Ithaca, NY: Cornell University Press;1967.
*Liminality
At a threshold, even a state of ambiguity, different from the past but not yet determined and with unbounded potential.

## Slide 22
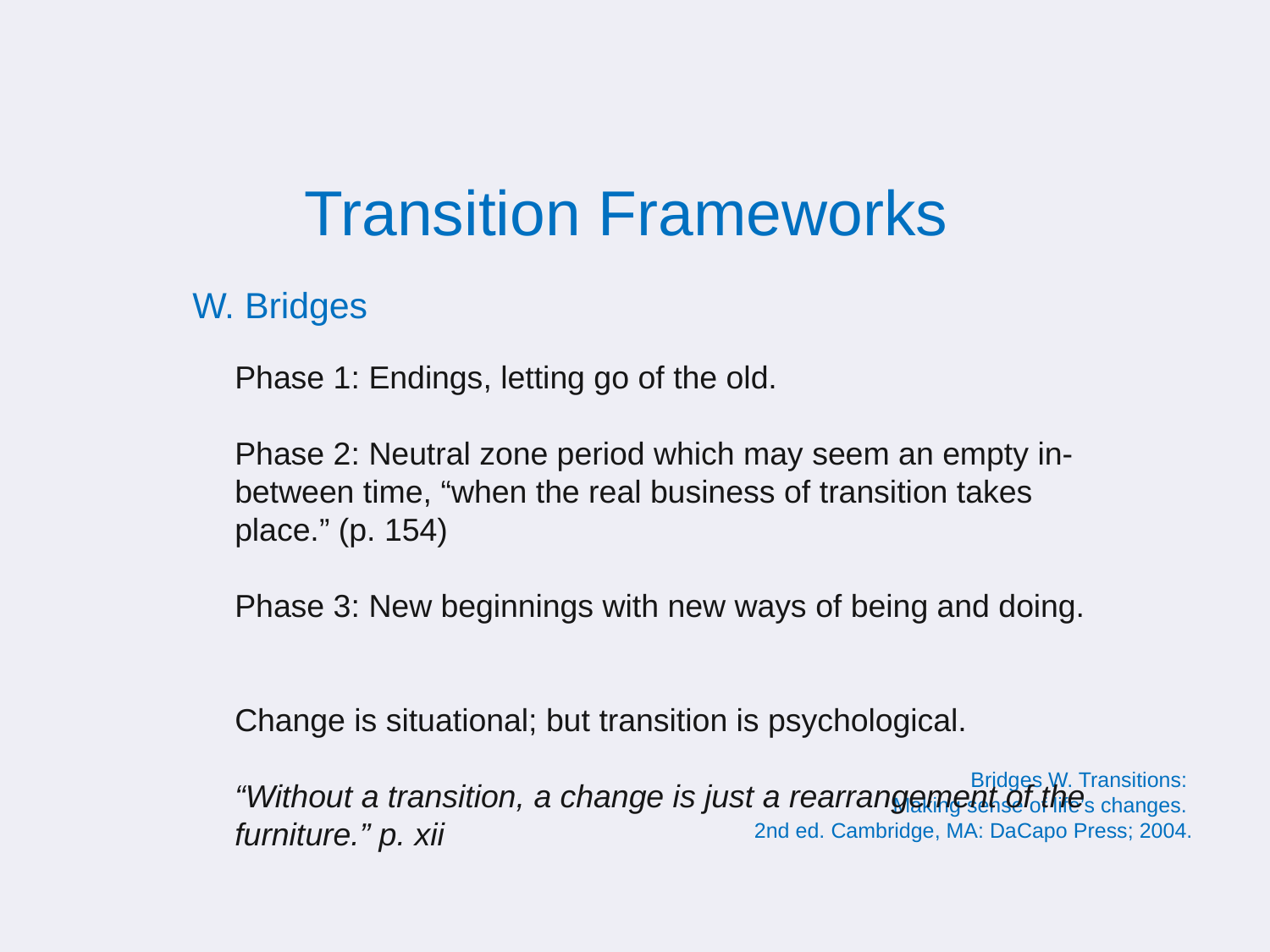

# Transition Frameworks
W. Bridges
Phase 1: Endings, letting go of the old.
Phase 2: Neutral zone period which may seem an empty in-between time, “when the real business of transition takes place.” (p. 154)
Phase 3: New beginnings with new ways of being and doing.
Change is situational; but transition is psychological.
“Without a transition, a change is just a rearrangement of the furniture.” p. xii
Bridges W. Transitions:
Making sense of life’s changes.
2nd ed. Cambridge, MA: DaCapo Press; 2004.

## Slide 23
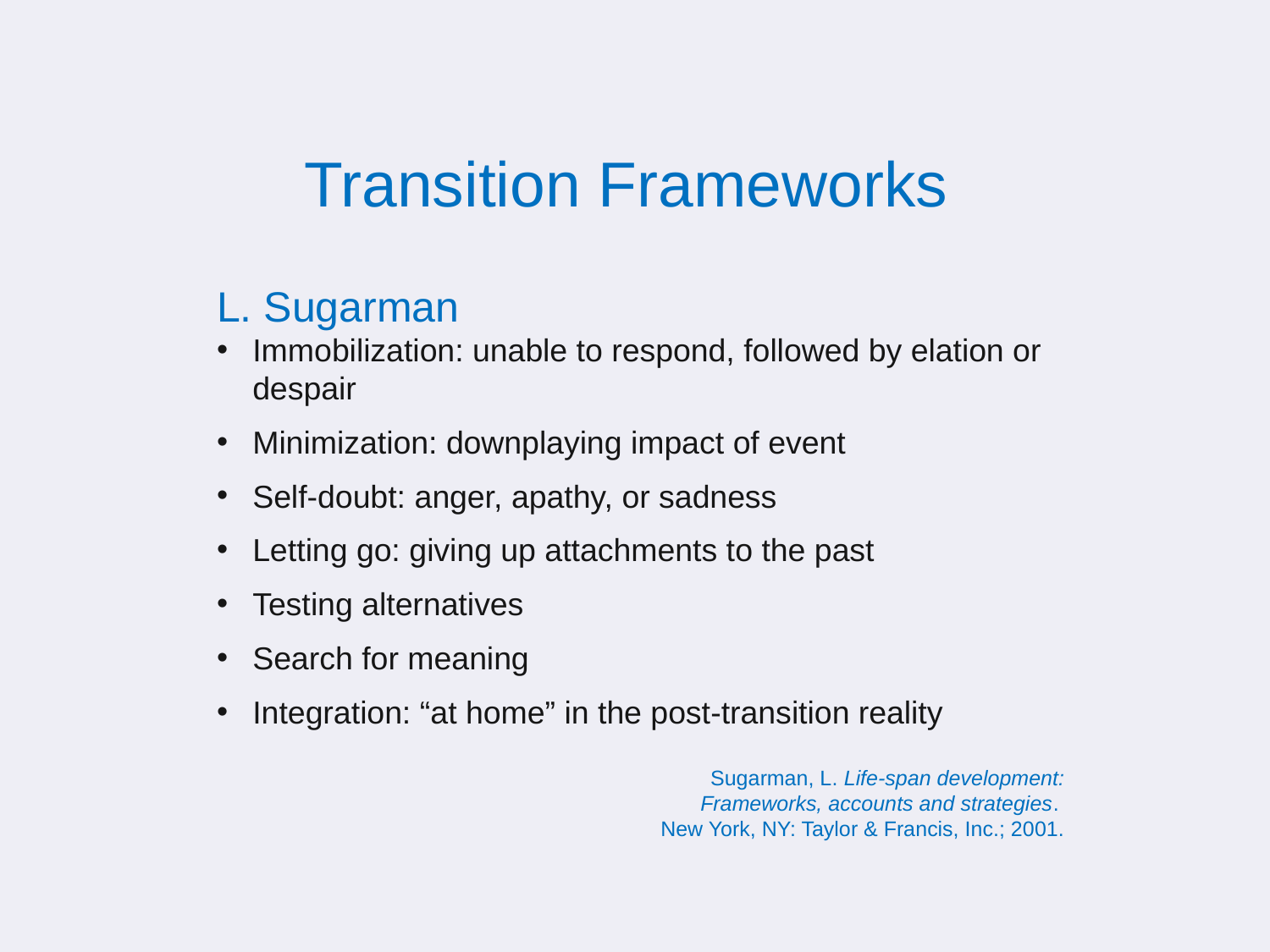

# Transition Frameworks
L. Sugarman
Immobilization: unable to respond, followed by elation or despair
Minimization: downplaying impact of event
Self-doubt: anger, apathy, or sadness
Letting go: giving up attachments to the past
Testing alternatives
Search for meaning
Integration: “at home” in the post-transition reality
			Sugarman, L. Life-span development:
Frameworks, accounts and strategies.
New York, NY: Taylor & Francis, Inc.; 2001.

## Slide 24
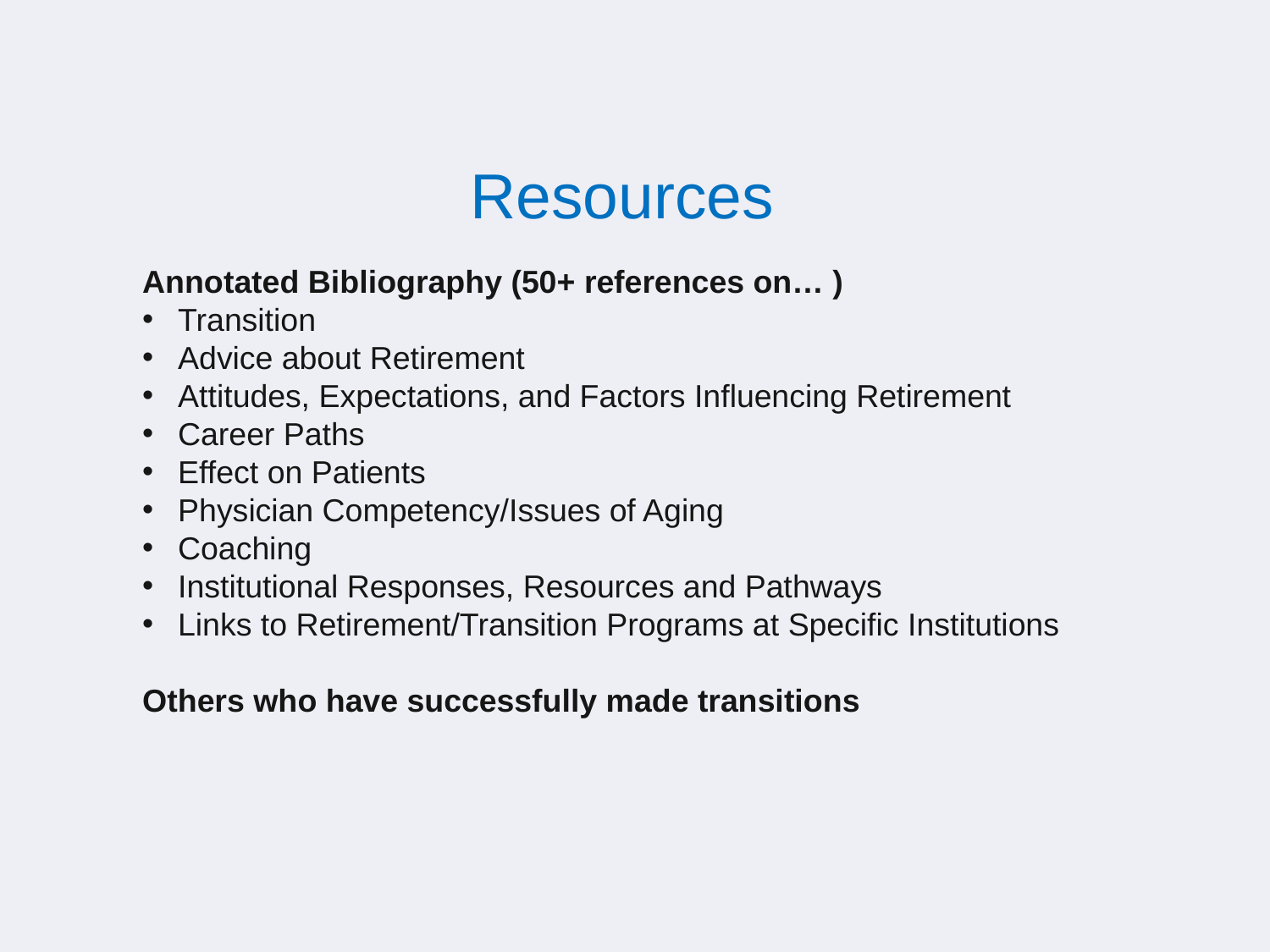

# Resources
Annotated Bibliography (50+ references on… )
Transition
Advice about Retirement
Attitudes, Expectations, and Factors Influencing Retirement
Career Paths
Effect on Patients
Physician Competency/Issues of Aging
Coaching
Institutional Responses, Resources and Pathways
Links to Retirement/Transition Programs at Specific Institutions
Others who have successfully made transitions

## Slide 25
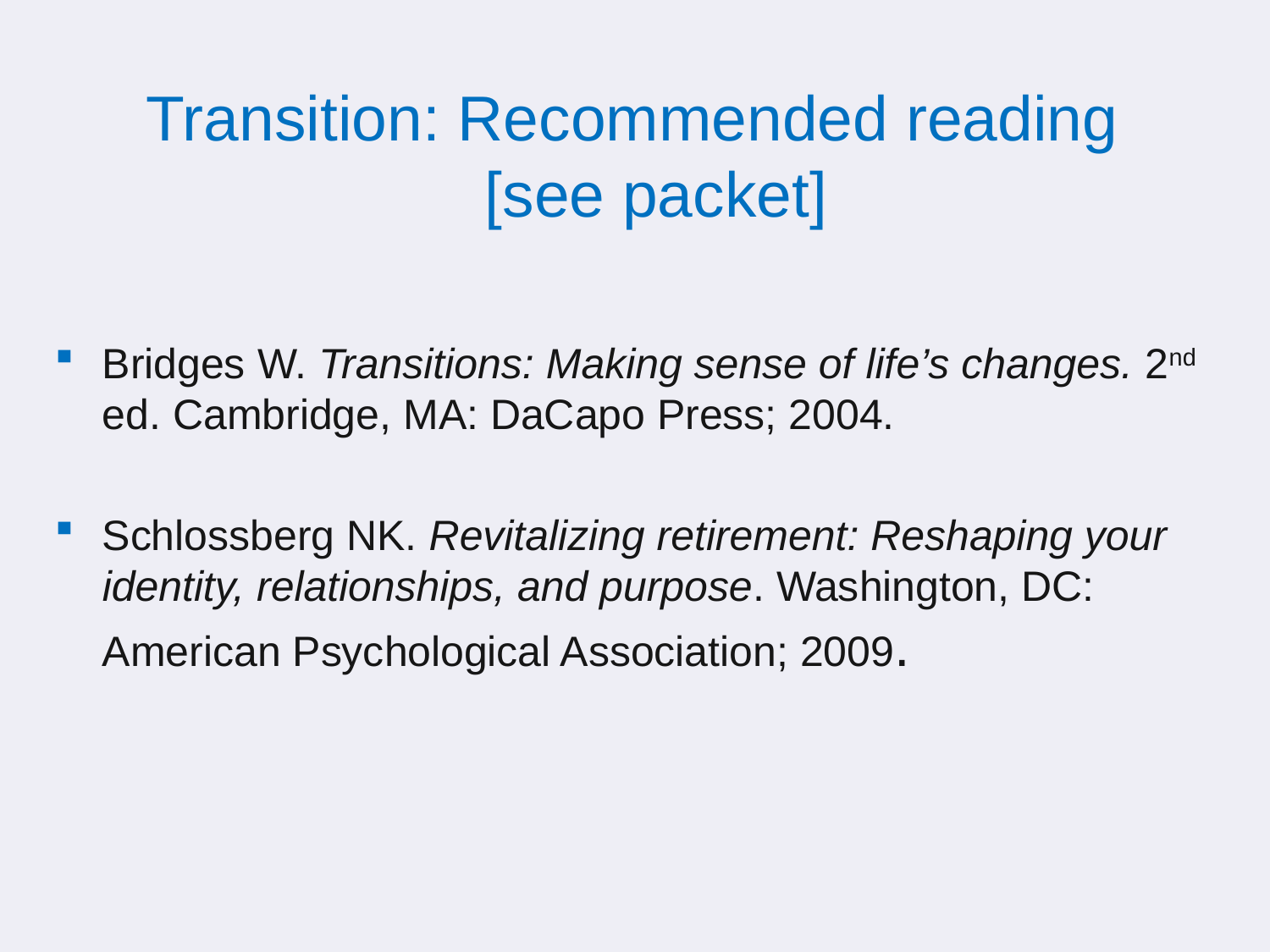

# Transition: Recommended reading [see packet]
Bridges W. Transitions: Making sense of life’s changes. 2nd ed. Cambridge, MA: DaCapo Press; 2004.
Schlossberg NK. Revitalizing retirement: Reshaping your identity, relationships, and purpose. Washington, DC: American Psychological Association; 2009.

## Slide 26
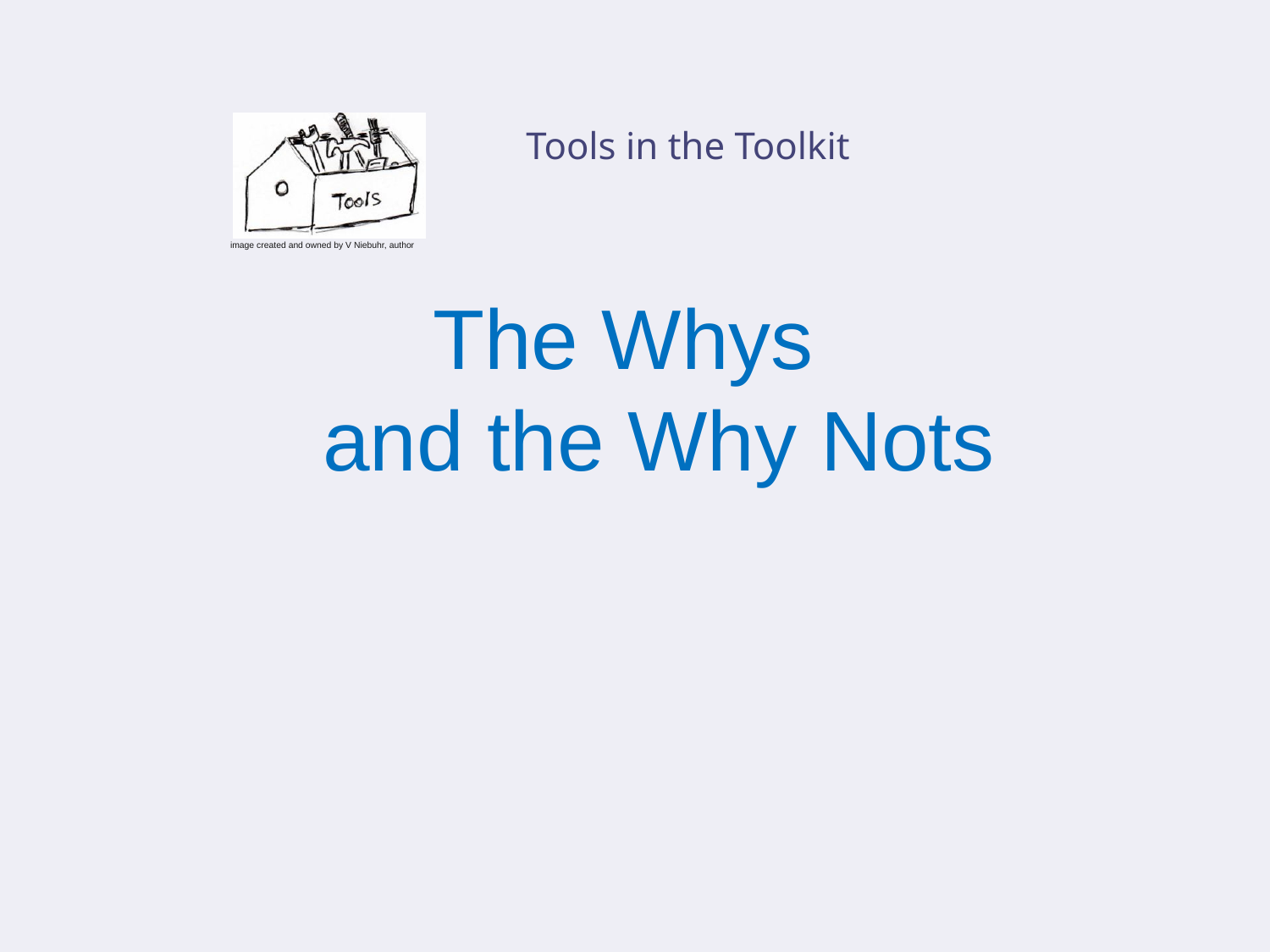

Tools in the Toolkit
image created and owned by V Niebuhr, author
# The Whys and the Why Nots

## Slide 27
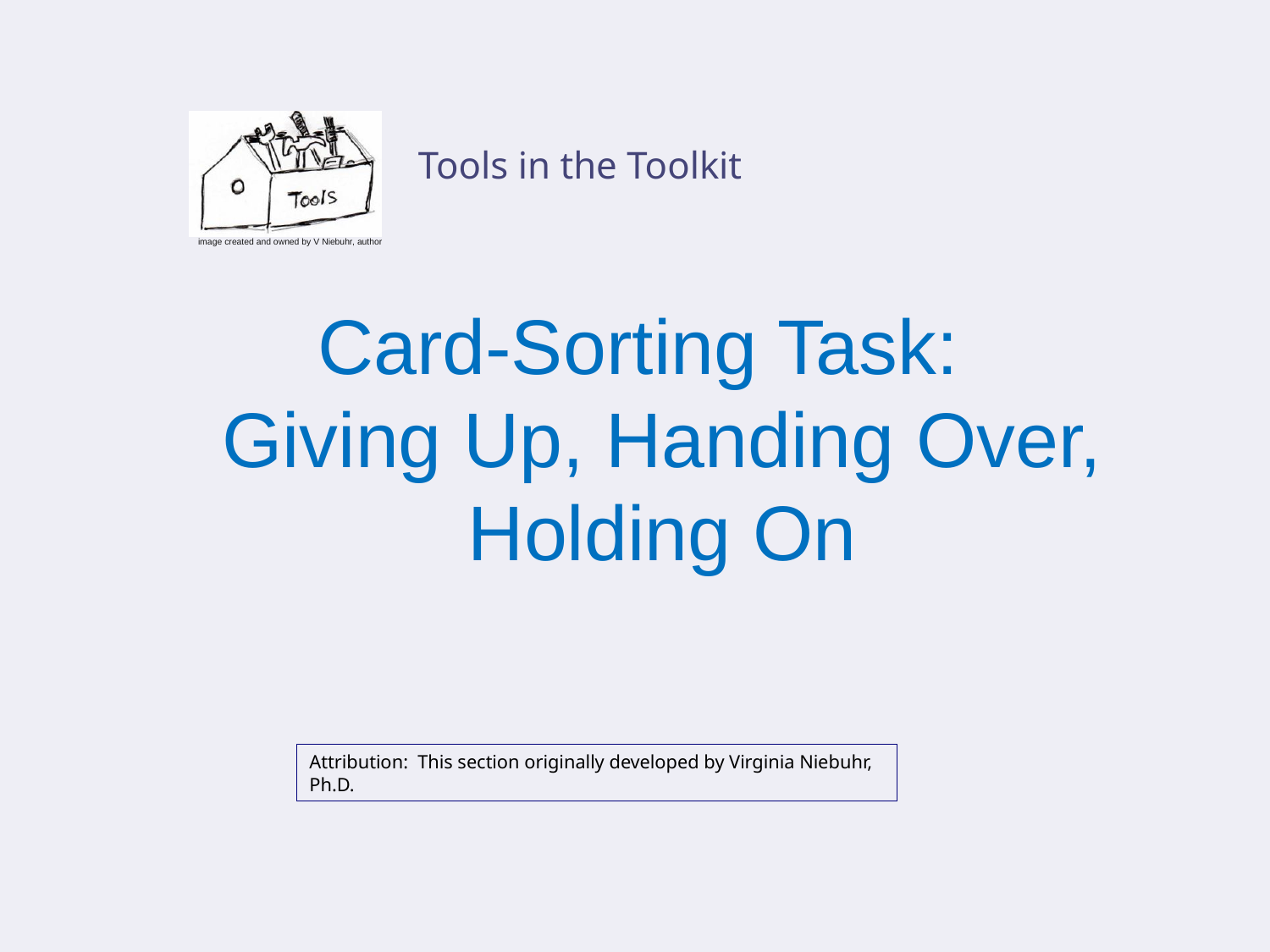

Tools in the Toolkit
image created and owned by V Niebuhr, author
# Card-Sorting Task: Giving Up, Handing Over, Holding On
Attribution: This section originally developed by Virginia Niebuhr, Ph.D.

## Slide 28
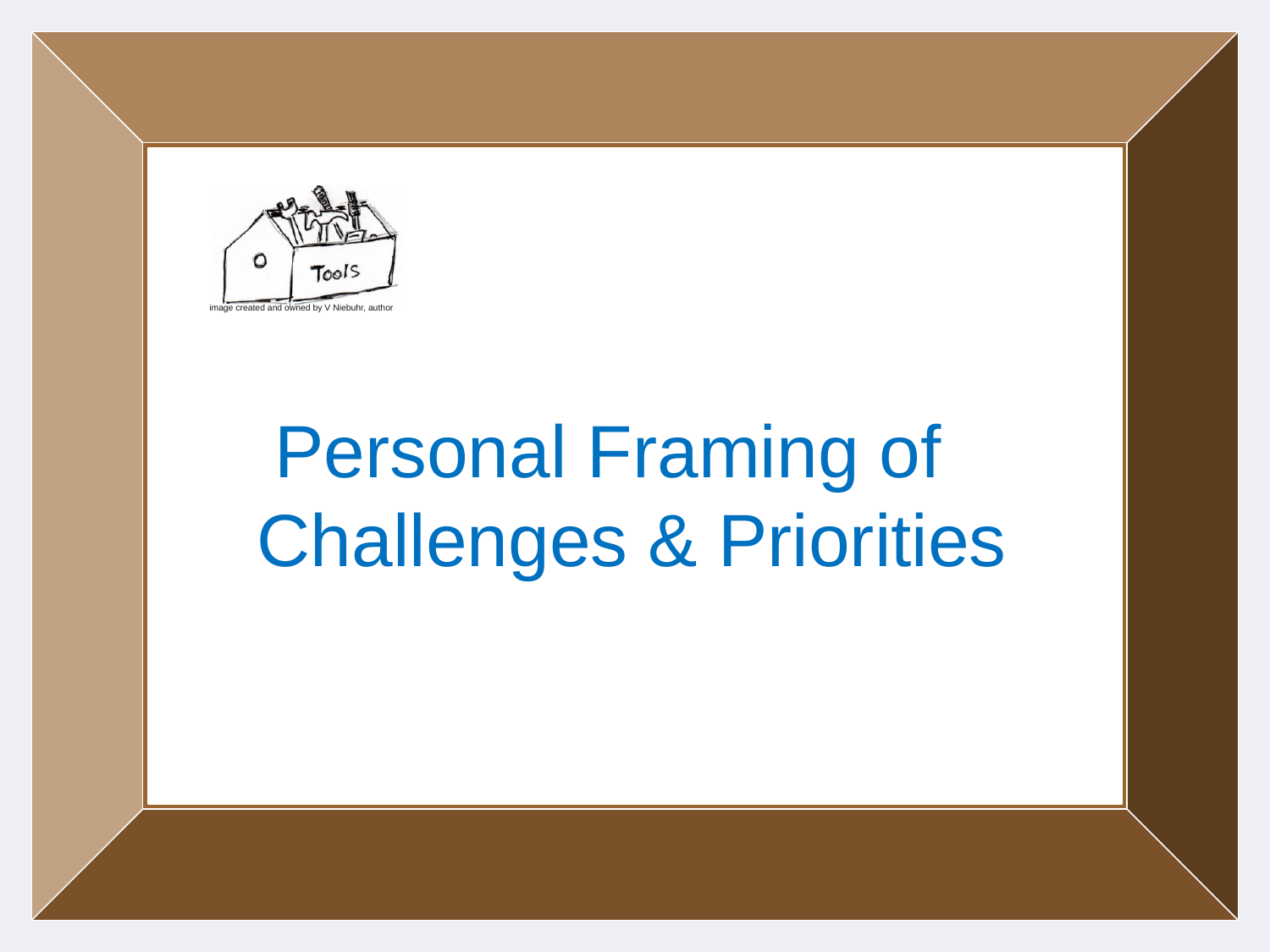

The Whys
and the Why Nots
image created and owned by V Niebuhr, author
# Personal Framing of Challenges & Priorities

## Slide 29
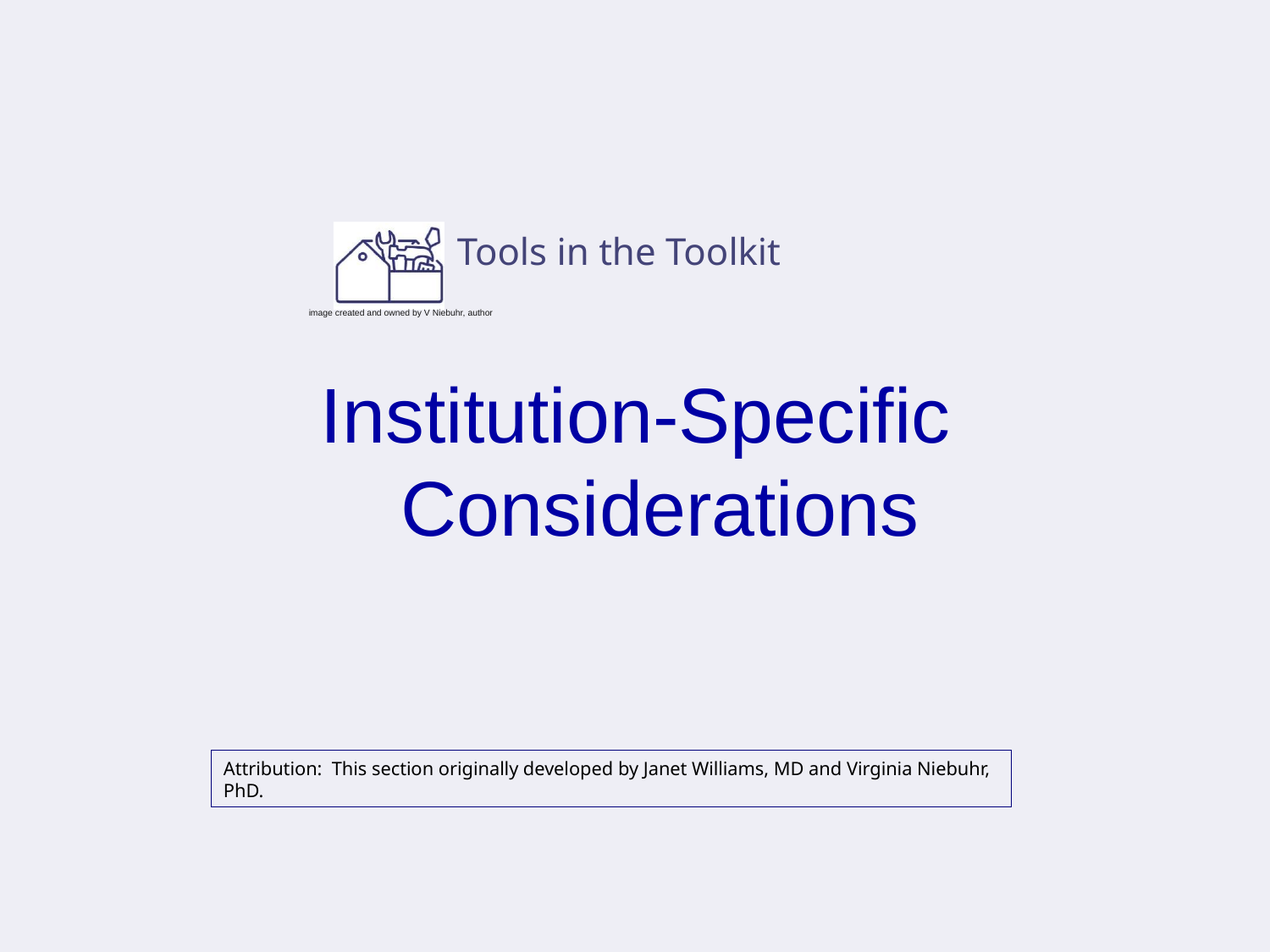

Tools in the Toolkit
image created and owned by V Niebuhr, author
# Institution-Specific Considerations
Attribution: This section originally developed by Janet Williams, MD and Virginia Niebuhr, PhD.

## Slide 30
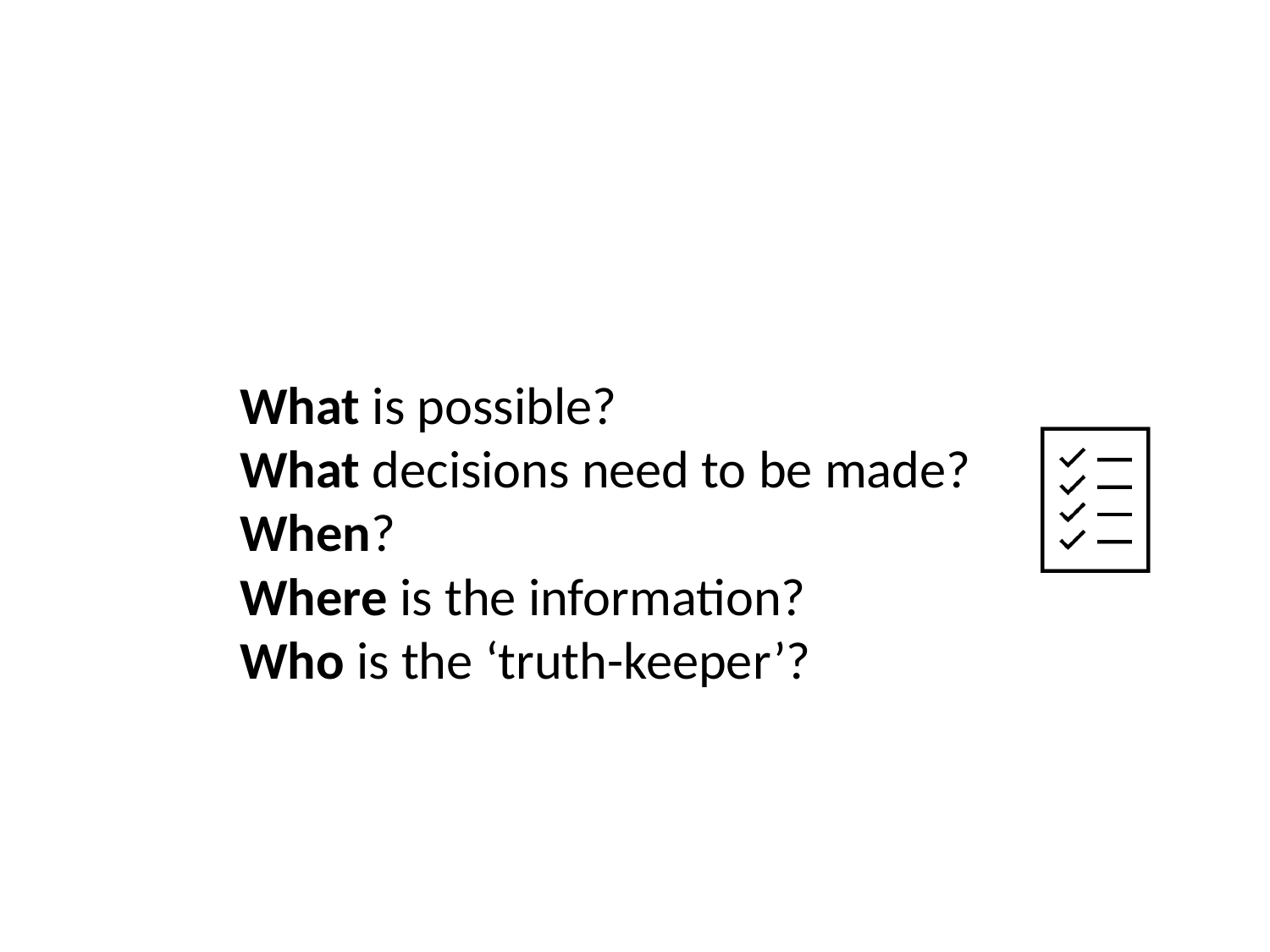

#
Institution-Specific Considerations
Do Not Assume!	 Ask! Question! Ask Again!
What is possible?
What decisions need to be made?
When?
Where is the information?
Who is the ‘truth-keeper’?

## Slide 31
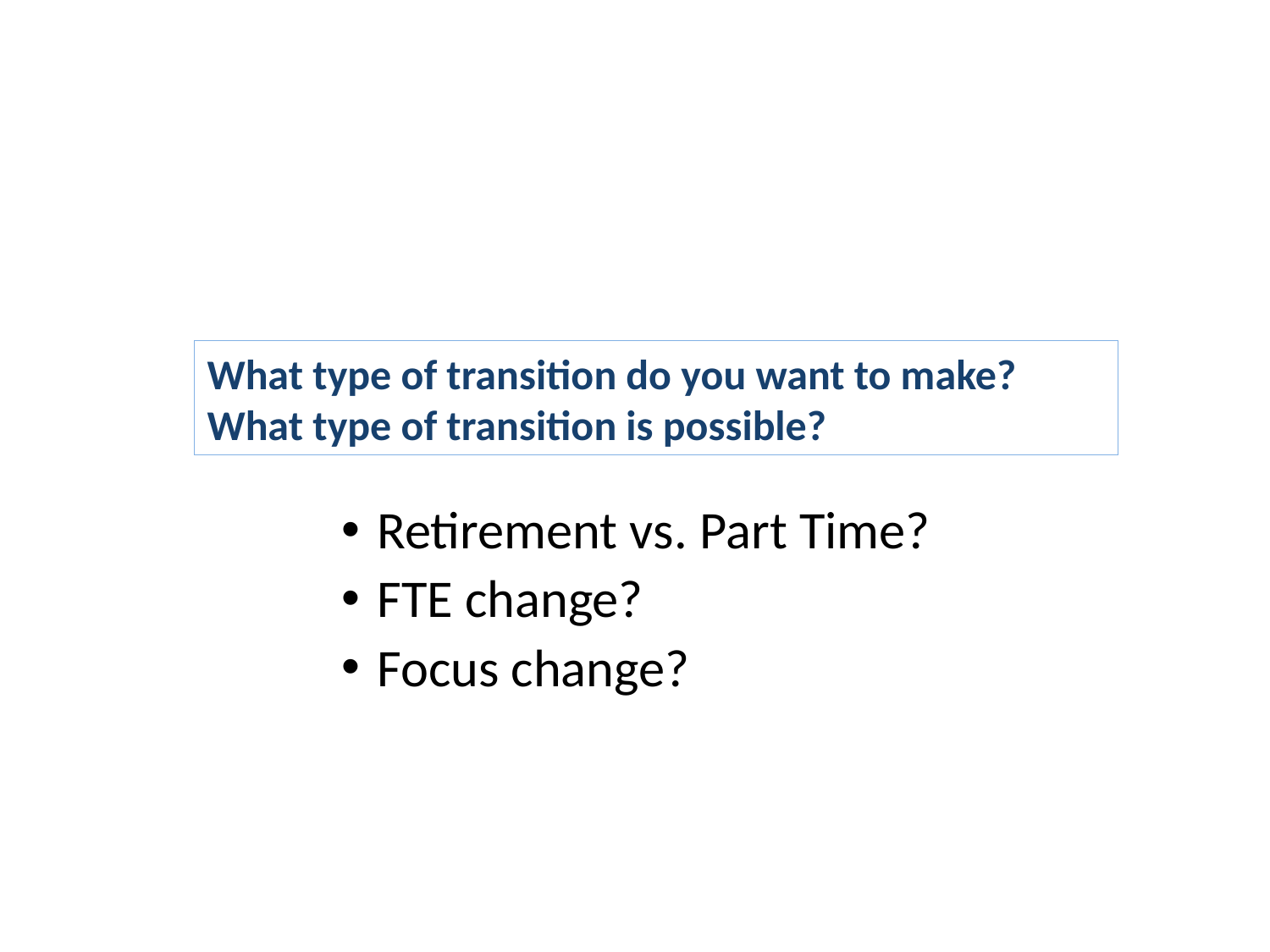

#
Institution-Specific Considerations
Do Not Assume!	 Ask! Question! Ask Again!
What type of transition do you want to make?
What type of transition is possible?
 Retirement vs. Part Time?
 FTE change?
 Focus change?

## Slide 32
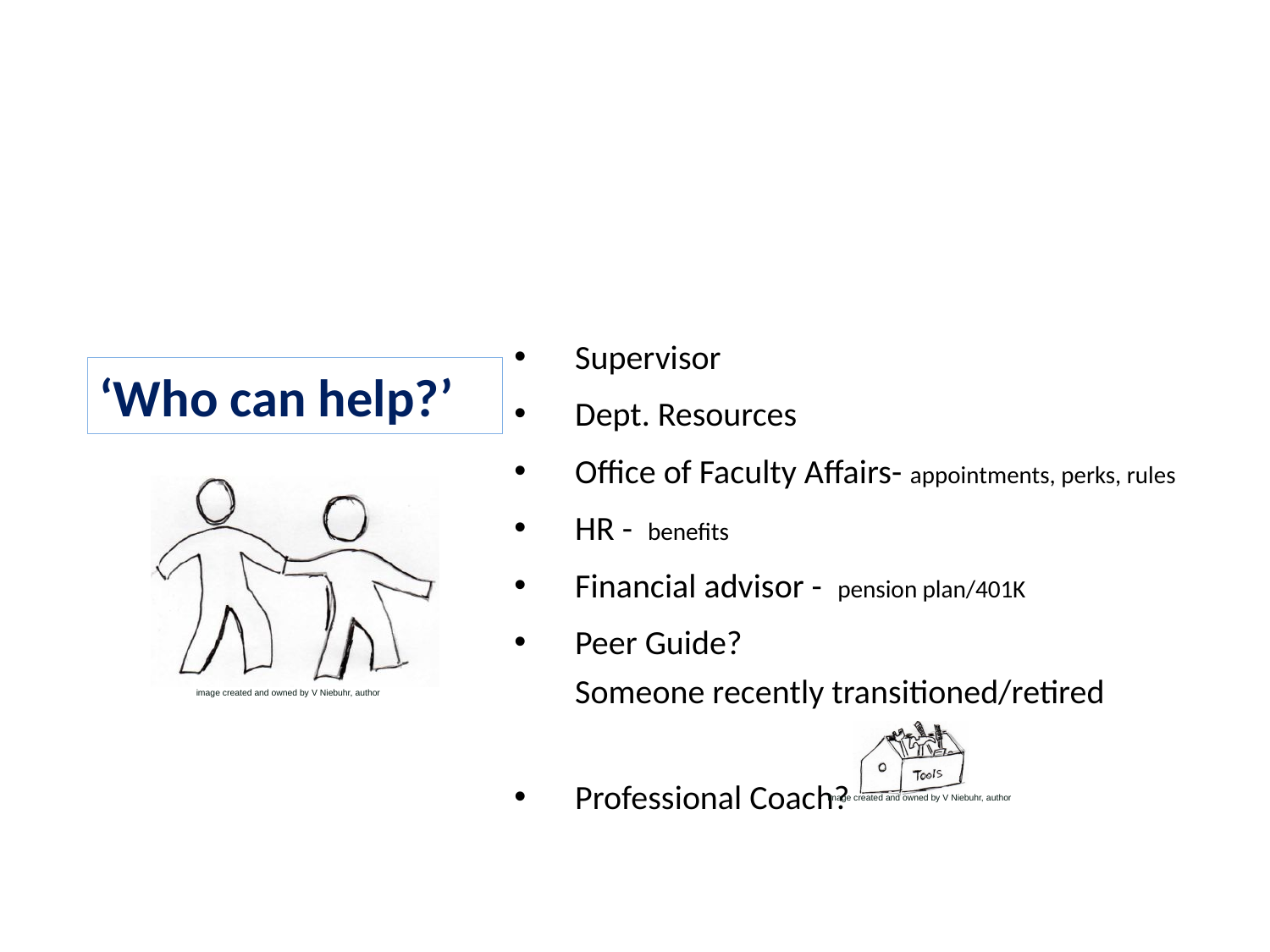

#
Institution-Specific Considerations
Do Not Assume!	 Ask! Question! Ask Again!
Supervisor
Dept. Resources
Office of Faculty Affairs- appointments, perks, rules
HR - benefits
Financial advisor - pension plan/401K
Peer Guide?
Someone recently transitioned/retired
Professional Coach?
‘Who can help?’
image created and owned by V Niebuhr, author
image created and owned by V Niebuhr, author

## Slide 33
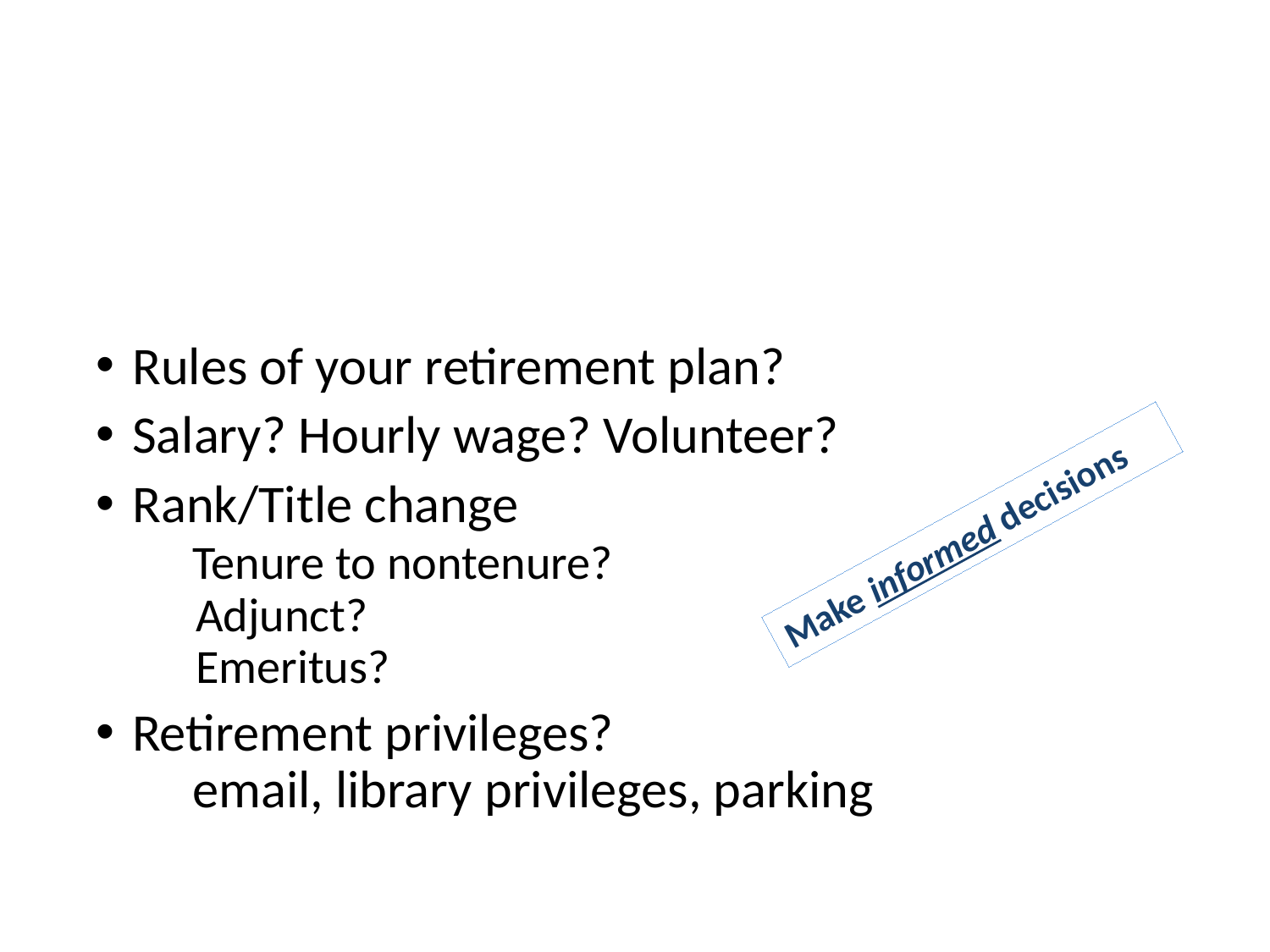

#
Institution-Specific Considerations
Do Not Assume!	 Ask! Question! Ask Again!
 Rules of your retirement plan?
 Salary? Hourly wage? Volunteer?
 Rank/Title change
 Tenure to nontenure?
 Adjunct?
 Emeritus?
 Retirement privileges?
 email, library privileges, parking
Make informed decisions

## Slide 34
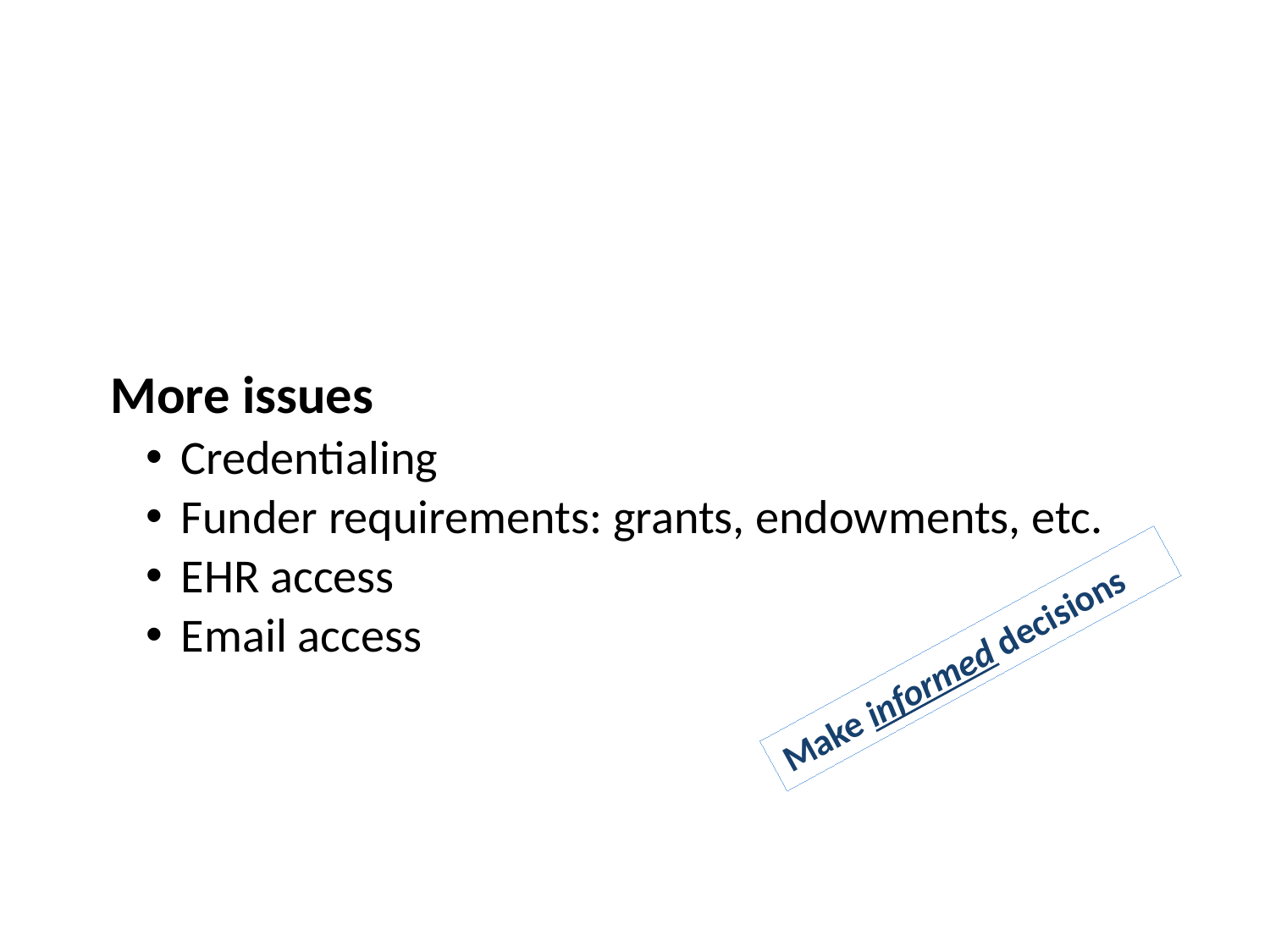

#
Institution-Specific Considerations
Do Not Assume!	 Ask! Question! Ask Again!
 More issues
 Credentialing
 Funder requirements: grants, endowments, etc.
 EHR access
 Email access
Make informed decisions

## Slide 35
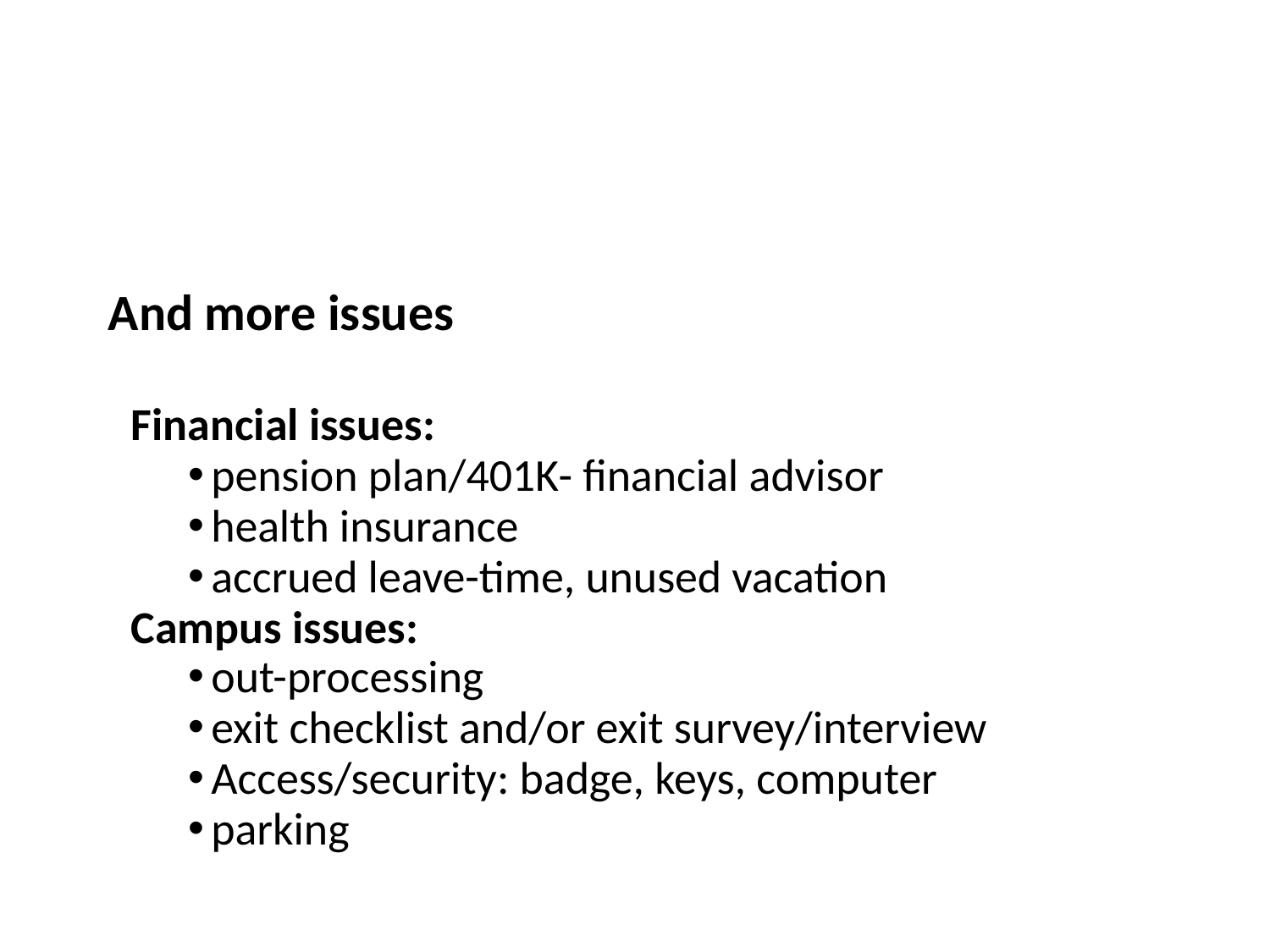

#
Institution-Specific Considerations
Do Not Assume!	 Ask! Question! Ask Again!
 And more issues
Financial issues:
pension plan/401K- financial advisor
health insurance
accrued leave-time, unused vacation
Campus issues:
out-processing
exit checklist and/or exit survey/interview
Access/security: badge, keys, computer
parking

## Slide 36
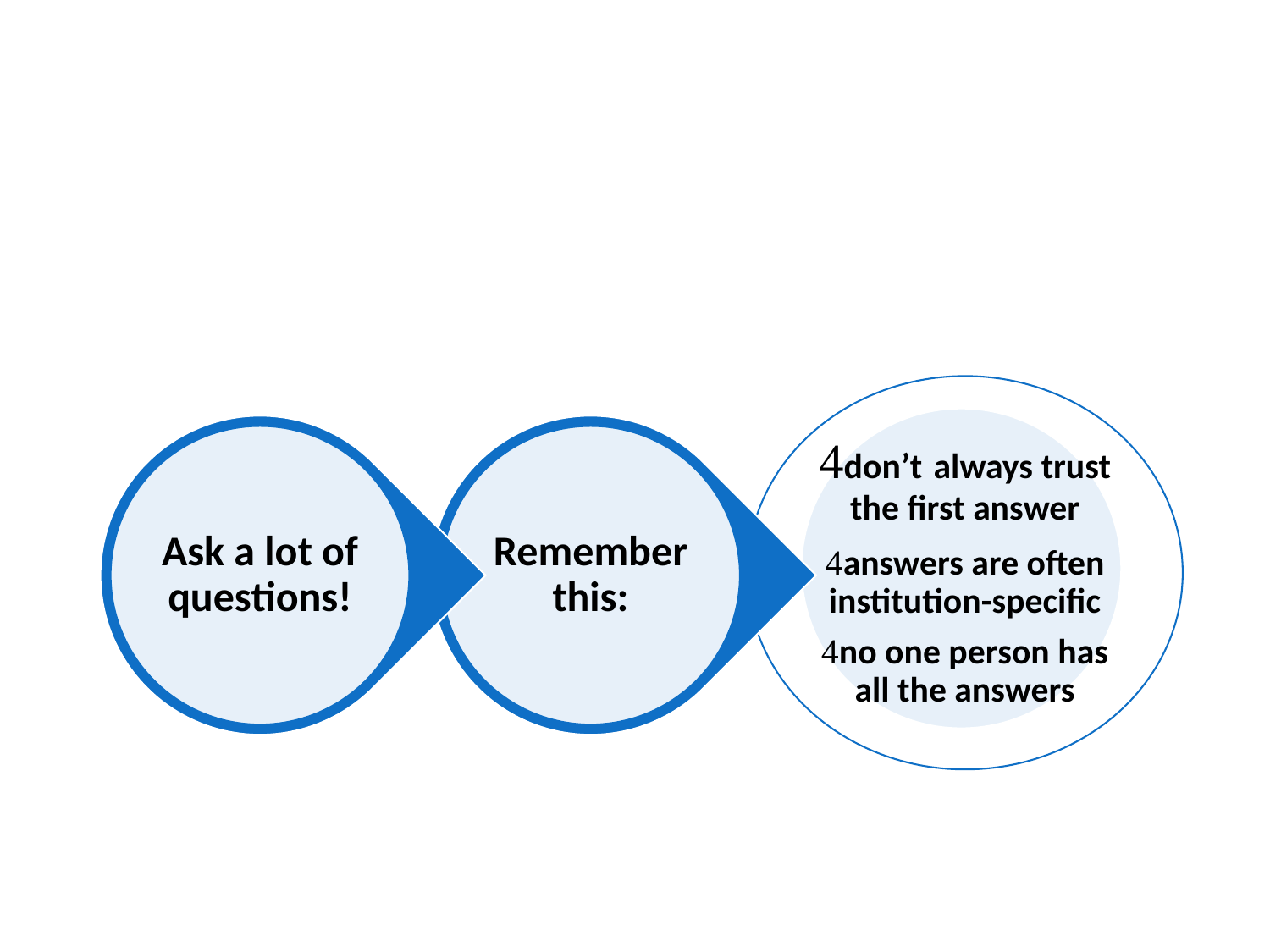

#
Institution-Specific Considerations
Do Not Assume!	 Ask! Question! Ask Again!

## Slide 37
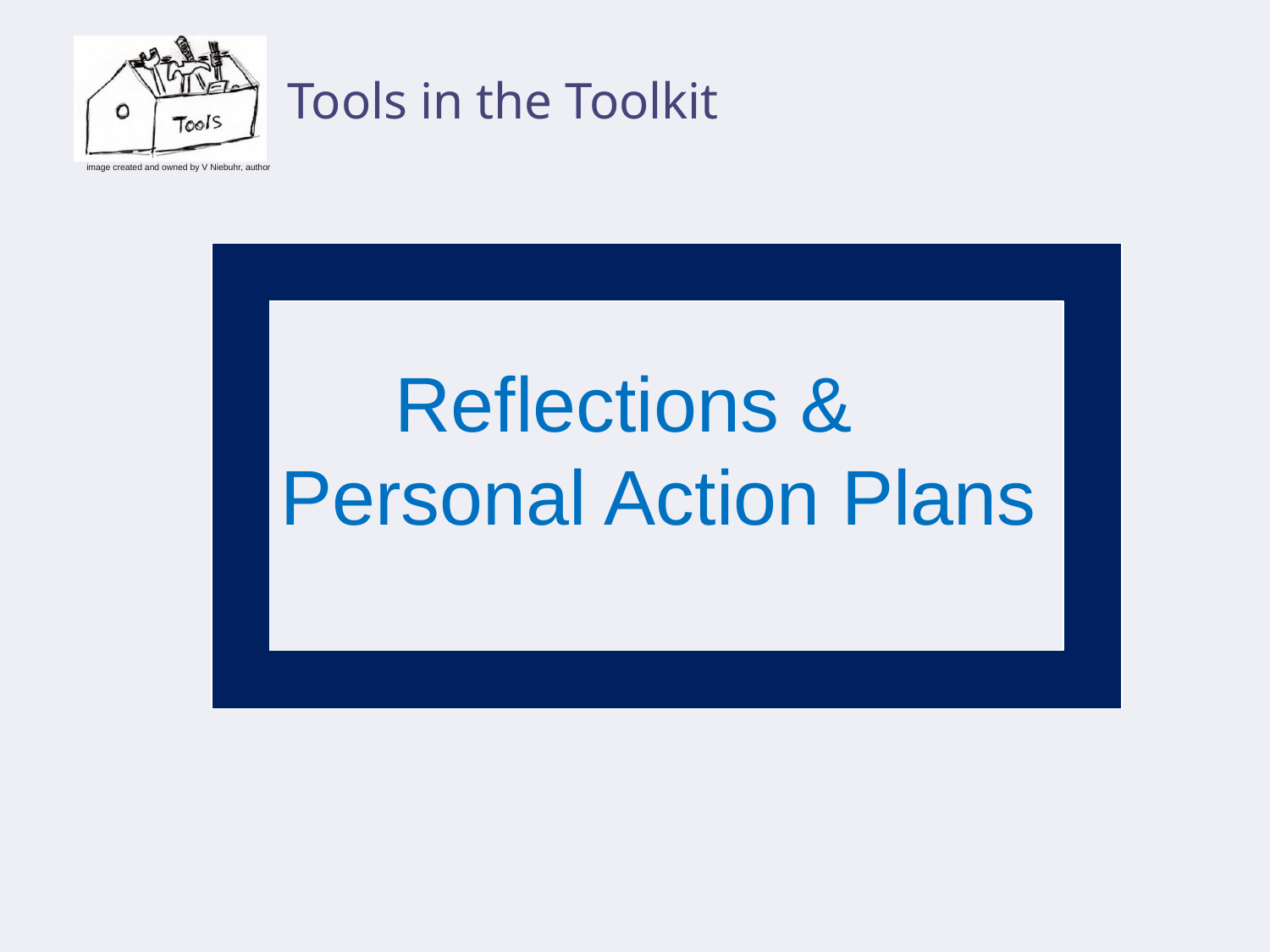

Tools in the Toolkit
image created and owned by V Niebuhr, author
# Reflections & Personal Action Plans

## Slide 38
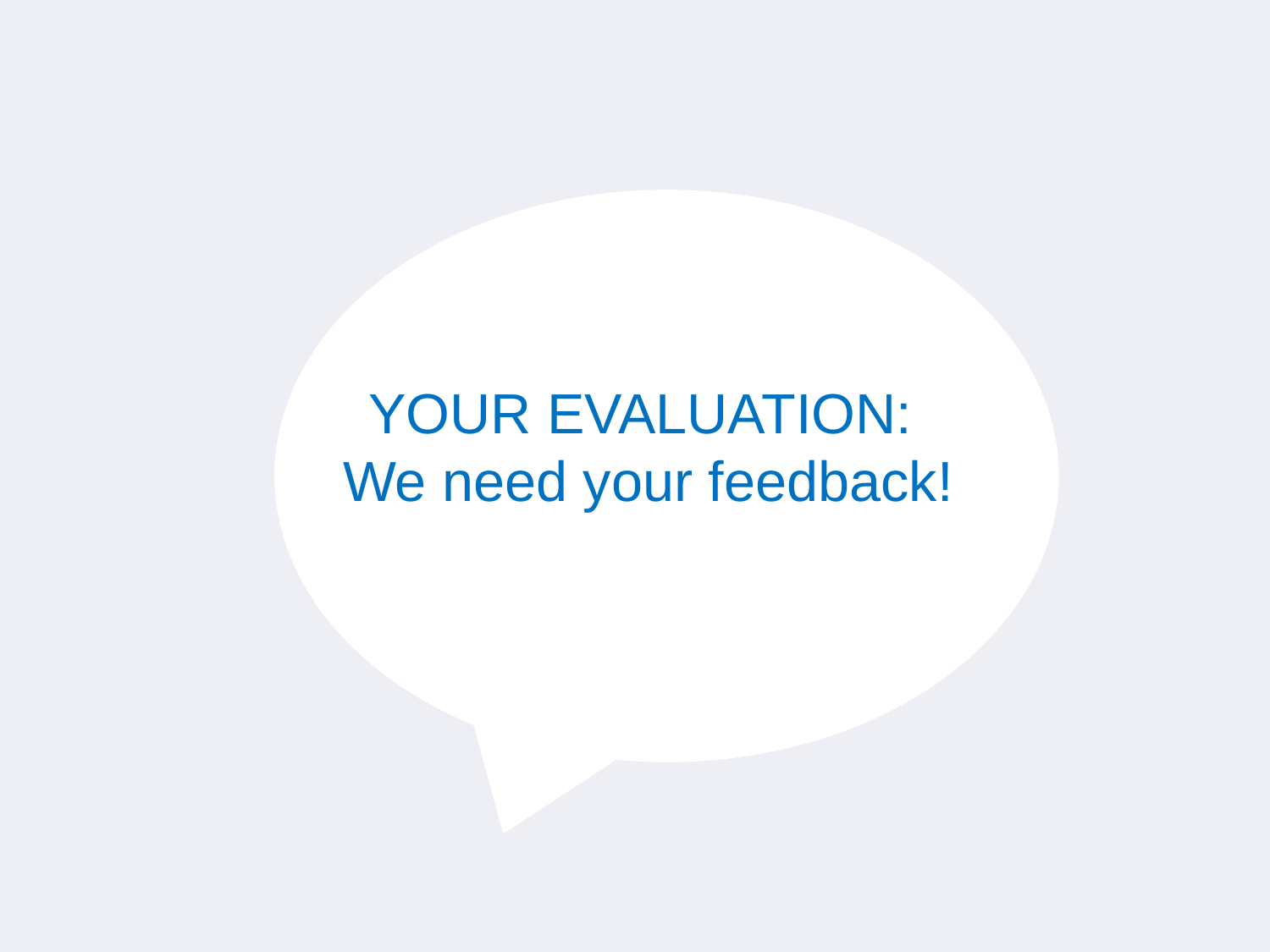

# YOUR EVALUATION: We need your feedback!
