# Supplementary material for: A Workshop and Toolkit to Support Late-Career Transitions for Faculty
Source: MedEdPORTAL. 2024 Nov 12;20:11463. doi: 10.15766/mep_2374-8265.11463 (PMC11554777; doi:10.15766/mep_2374-8265.11463)
Supplement: Supplementary file 1 — Packet-Toolkit.docxToolkit Slides.pptxFacilitators Guide.docxWorkshop Evaluation.docx [file mep_2374-8265.11463-s001.zip › D. Workshop Evaluation.docx]

Workshop Evaluation

*1. Your current degree

| ⃝ MD/DO | ⃝ PhD | ⃝ MD/PhD |
| --- | --- | --- |
|  |  |  |
| ⃝ Other (please specify) | | |
|  | | |

*2. Please describe your current responsibilities (check ALL that apply)

| 🞏 | Clinician (primary care) |
| --- | --- |
| 🞏 | Clinician (specialty) |
| 🞏 | Educator |
| 🞏 | Researcher |
| 🞏 | Administrator |
| 🞏 | Other (please specify) |
|  |  |

*3. Describe the nature of the late career transition you are contemplating (check ALL that apply)

| 🞏 | Change to a new position or career |
| --- | --- |
| 🞏 | Shift to part-time, without retiring |
| 🞏 | Retirement, with continued part-time employment |
| 🞏 | Retirement, with no plans for part-time employment |
| 🞏 | Retirement, with plans for part-time uncompensated, voluntary professional activities |
| 🞏 | Other (please specify) |
|  |  |

*4. Describe your current status with respect to making a late career transition (select ONE BEST answer):

| ⃝ | Thinking about transition in general terms |
| --- | --- |
| ⃝ | Making specific plans for a transition within the next 1-5 years |
| ⃝ | Currently moving through transition |
| ⃝ | Have fully transitioned- have retired or shifted to part-time without retiring |
| ⃝ | Have fully transitioned- change to a new position or career |
| ⃝ | Other (please specify) |
|  |  |

*5. Your age:

| ⃝ | <35 yrs |
| --- | --- |
| ⃝ | 36-45 |
| ⃝ | 46-55 |
| ⃝ | 56-60 |
| ⃝ | 61-65 |
| ⃝ | 66-70 |
| ⃝ | >70 yrs |

*6. Most helpful aspect of today’s workshop:

|  |
| --- |

*7. Least helpful aspect of today’s workshop:

|  |
| --- |

*8. Suggestions for improving the workshop:

|  |
| --- |

*9. Following today’s workshop, how confident are you that you can:

|  | \|  \| Not at all confident \| Not confident \| Neutral \| Confident \| Extremely confident \| \| --- \| --- \| --- \| --- \| --- \| --- \| \| Self-evaluate personal challenges for making a late career transition \| ⃝ \| ⃝ \| ⃝ \| ⃝ \| ⃝ \| \| Select elements of a transition framework meaningful for your own circumstances \| ⃝ \| ⃝ \| ⃝ \| ⃝ \| ⃝ \| \| Identify personal priorities and action strategies for career transitions \| ⃝ \| ⃝ \| ⃝ \| ⃝ \| ⃝ \| \| Identify resources that might be helpful to facilitate transitions \| ⃝ \| ⃝ \| ⃝ \| ⃝ \| ⃝ \| \| Comments \|  \| \| \| \| \| \|  \| \| \| \| \| \| |
| --- | --- | --- | --- | --- | --- | --- | --- | --- | --- | --- | --- | --- | --- | --- | --- | --- | --- | --- | --- | --- | --- | --- | --- | --- | --- | --- | --- | --- | --- | --- | --- | --- | --- | --- | --- | --- | --- | --- | --- | --- | --- | --- | --- |
|  |  |
|  |  |
|  |  |
