# Supplementary material for: A Workshop and Toolkit to Support Late-Career Transitions for Faculty
Source: MedEdPORTAL. 2024 Nov 12;20:11463. doi: 10.15766/mep_2374-8265.11463 (PMC11554777; doi:10.15766/mep_2374-8265.11463)
Supplement: Supplementary file 1 — Packet-Toolkit.docxToolkit Slides.pptxFacilitators Guide.docxWorkshop Evaluation.docx [file mep_2374-8265.11463-s001.zip › _Educational Summary Report_11463.pdf]

MedEdPORTAL® | The AAMC Journal of Teaching and Learning Resources

Original Publication Open Access

A Workshop and Toolkit to Support Late-Career Transitions for Faculty

Virginia Niebuhr, PhD*, Pamela Wood, MD, Judith Livingston, PhD, Fred Henretig, MD, MSHP, Andrew Sirotnak, MD, Janet Williams, MD, David Jaffe, MD

*Corresponding author: vniebuhr@utmb.edu

Abstract

Introduction: Twenty percent of medical school faculty are 60 years or older. These senior-career academic faculty often find a paucity of support for decision-making about late-career transitions. **Methods:** To help fill this professional development gap, we developed and tested an interactive workshop to facilitate deliberation and discussion among mid- and late-career faculty in various stages of career transition planning. The workshop included individual and small-group activities and a takeaway packet/toolkit for career transition planning. We conducted mixed methods analyses of postworkshop survey data to evaluate the workshop content, methods, and toolkit. **Results:** The workshop was implemented six times, at four national conferences, one state-level conference, and one single institution, for a total of 207 participants. Evaluations were completed by 60% of participants. Thirty-one percent were thinking generally about transition, 40% were making specific plans for transition within 1-5 years, 15% were currently in transition, and 10% had fully transitioned. Perception of workshop objectives met received a median ranking of 4 (rated on a 5-point Likert scale). Participants reported positive outcomes, including taking time to think/reflect, finding starting points for transition planning, and sharing conversations. **Discussion:** This highly rated, interactive workshop provides support and practical tools for faculty considering late-career transitions. In addition to providing resources for individual faculty, this workshop has value for an institution to support their senior-level faculty. We encourage the incorporation of this workshop into institutional faculty development programming to ensure a career lifespan approach to faculty development.

Keywords
Late-Career Transitions, Retirement, Professional Development, Continuing Professional Development, Faculty Affairs, Mental Health/Well-Being

Educational Objectives

By the end of this activity, learners will be able to:

1. Self-evaluate personal challenges for making a late-career transition.
2. Select elements of transition models that are meaningful for their own circumstances.
3. Identify personal priorities and action strategies for late-career transitions.
4. Identify resources that might be helpful to facilitate these transitions.

Citation:
Niebuhr V, Wood P, Livingston J, et al. A workshop and toolkit to support late-career transitions for faculty. *MedEdPORTAL*. 2024;20:11463. https://doi.org/10.15766/mep_2374-8265.11463

Introduction

The average age of medical school faculty has increased over the past 30 years, such that 21% of faculty are now 60 years of age or older.[1,2] Older faculty often want to consider late-career transitions such as retirement, part-time, or volunteer work. There is evidence that senior-career faculty can continue to contribute to their profession and their institutions postretirement. Retired faculty who feel valued may continue to participate in teaching, mentoring, advocacy, and clinical and research activities; and they may be willing to do so as volunteers.[3,4]

Late-career transitions generally bring anxiety about changes in professional identity;[5,6] and academic faculty are often unsupported in considering or planning effective late-career transitions. Unlike the multitude of professional development opportunities for junior and mid-level faculty, there are relatively few opportunities for senior-career faculty to explore the logistics and implications of career shifts. Initiatives that support late-career transition planning could provide much-needed assistance

Copyright © 2024 Niebuhr et al. This is an open-access publication distributed under the terms of the Creative Commons Attribution license.

to individual faculty and could also help promote the clinical, research, and teaching missions of their institutions.

Others have produced materials to help faculty with change. Thorndyke and colleagues[7] developed a case-based workshop to facilitate transition to new leadership positions but did not address transitioning out of full-time academic careers. Palmer and colleagues[8] focused on the role of academic leaders, using a small-group case-based approach to help institutional leaders explore vitality needs of senior-career and emeritus faculty but did not provide specific tools for faculty themselves.

To help fill this professional development gap, we developed and tested a workshop to facilitate conversation and provide tools for making senior-career transitions, including retirement. Choices for workshop activities were grounded in transition literature[9-14] and our own transition experiences. The workshop is designed to provide a forum for exploration with other professionals who are approaching, moving through, or have made late-career transitions.

We grounded our methods in adult learning principles: adult learners contribute to their own learning and that of their peers, need internal motivation, are solution-oriented, and learn on a need-to-know basis. Therefore, our methods included individual, small-group, and large-group activities, worksheets, card-sorting tasks, and case-based discussions. Participants have opportunities to evaluate their perceptions about transition, identify personal priorities, and generate potential strategies for addressing specific challenges. They leave with a personal action plan and a packet/toolkit with resources, including an annotated bibliography. In this Educational Summary Report, we report results of a mixed quantitative and qualitative evaluation of six iterations of this workshop. We present participants' evaluations of the workshop, outcome measures, and a discussion of lessons learned and recommendations for adoption by others.

Methods

Facilitators and Audiences

The workshop was developed by five senior-career academic pediatric faculty at various stages of late-career transitions and from different academic medical centers. The workshop was designed for mid- or senior-career faculty interested in, or in the process of, late-career transitions. It is also appropriate for departmental leaders and administrators who mentor and support faculty in making career transitions.

The workshop was conducted six times from May 2019 to May 2023. For Workshops #1, #3, #5, and #6, proposals were peer-reviewed; and the workshops were presented at a national discipline-specific (pediatrics) academic conference with available CME credits. Workshop #4 was also peer-reviewed and presented at a state-level conference for multidiscipline health professions educators. Workshop #2 was by invitation, for faculty from any of five schools of a single academic health science center. Workshops #3 and #4 were conducted through videoconferencing during the COVID-19 pandemic; the others were in person.

Implementation

The workshop was originally designed to take 3 hours, but we have adapted it to 120 or 90 minutes, when necessary. The choice of activities can be tailored to the specific audience and available time frame. Small-group work is best accomplished in groups of six to eight participants, at tables or using virtual breakout rooms.

A printed packet/toolkit (Appendix A) was important for each participant to use while working through activities and as a resource for use after the workshop. The only materials needed for this workshop were the printed packet/toolkit and a stack of index cards for each participant. Projection was needed for the slide set (Appendix B). The facilitators guide (Appendix C) provides more detailed suggestions on implementation and tailoring.

Workshop Content

The workshop included 10 elements. Appendix C (facilitators guide) has more in-depth descriptions of each element, including recommended times for a 2-hour workshop and alternative timings for longer or shorter workshops.

- Introduction: facilitators' stories about their own transitions
- Individual and small-group activity: "Metaphors for Late-Career Transition"
- Brief didactic: "Published Literature on Late-Career Transitions"
- Brief didactic: "Frameworks and Resources"—defines transition and reviews three transition frameworks
- Individual and small-group activity: "The Whys and the Why Nots"
- Individual activity: "Card Sorting Task: Giving Up, Handing Over, Holding On"
- Individual and small-group activity: "Case Discussions" or "Personal Framing of Challenges & Priorities"
- Brief didactic: "Institution-Specific Considerations"—includes specific institutional policies/procedures to consider

Copyright © 2024 Niebuhr et al. This is an open-access publication distributed under the terms of the Creative Commons Attribution license.

- Individual activity: “Personal Action Plan”
- Closing and workshop evaluation

Evaluation
The University of Texas Health Science Center at San Antonio Institutional Review Board determined further review of this project not necessary. Immediate postworkshop evaluations were obtained through surveys delivered on paper for the first two workshops and by web-based survey for the subsequent workshops. Demographic data included academic degree, professional responsibilities, contemplations about their own late-career transitions, current stage of transition, and age. We assessed participants’ confidence for attainment of each objective using a 5-point Likert scale. Open-ended questions addressed what was most and least helpful and invited suggestions for improvement (Appendix D).

Quantitative data were analyzed using descriptive statistics. We report Likert scale analyses as medians (interquartile range) because data are not normally distributed. Qualitative data from open-ended survey questions (workshops #1-5) were analyzed using guidelines from Glesne.[15] Three workshop team members independently developed coding schemes, coded responses, identified themes and subthemes, and then shared coding schemes with each other. Schemes were discussed until consensus was reached on major themes.

Results

A total of 207 participants attended six workshops, and the number per session ranged from 16 to 51. Sixty percent of participants ($n = 124$) completed a workshop evaluation. Most respondents had MD/DO degrees (69%) or MD/PhD degrees (6%); others had PhD (15%) or other degrees (9%). Workshop #2, presented at a single institution, had more respondents with PhD (37%) or other degrees (14%). Overall, 22% were younger than 56 years of age, 43% were 56 to 65, and 33% were older than 65. Few respondents were fully retired with no professional responsibilities (5%). Most nonretirees (75%) had multiple professional responsibilities, including clinician, educator, researcher, and/or administrator.

Contemplating their own futures, participants were considering change to a new position/career (34%), shift to part-time without retiring (34%), retirement with part-time employment (31%), retirement with no continued employment plans (15%), retirement with volunteer professional activities (20%), or other unspecified plans (14%). Related to their transition stage, 31% were thinking about transition in general terms, 40% were making specific plans for transition within 1 to 5 years, 15% were currently moving through transition, 8% had fully transitioned to retirement or part-time, and 2% had transitioned to a new position/career. Several participants stated that they attended the workshop so that they could support faculty at their own institution.

Confidence ratings for achieving workshop objectives were high, with a median ranking of 4 for each of the objectives (rated on a 5-point Likert scale: Table 1).

In evaluations from the first five workshops, 98 participants provided narrative comments in response to open-ended questions. Twenty-one participants provided summary comments. Qualitative analysis of all narrative comments revealed three major themes: workshop elements, workshop process, and outcomes.

Regarding workshop elements (i.e., activity type), every element was described as *most helpful* by at least one participant. For some elements, it was the small-group discussion about the activity that was most important. Several participants stated that the mix of elements was the key workshop strength. There were

Table 1. Postsession Ratings of Learning Objectives[a]

Objectives	*n*	% Responding: Not at All Confident	Not Confident	Neutral	Confident	Extremely Confident	*Mdn*[b]	Percentiles 25th, 75th[b]
Self-evaluate personal challenges for making a late-career transition.	120	3	3	13	73	8	4	4, 4
Select elements of transitions models that are meaningful for your own circumstances.	65	2	3	15	68	12	4	4, 4
Identify personal priorities and action strategies for late-career transitions.	120	3	4	13	68	12	4	4, 4
Identify resources that might be helpful to facilitate these transitions.	66	2	2	26	55	17	4	3, 4
Identify frameworks and resources that might be helpful to facilitate transitions.	52	4	4	19	62	12	4	3, 4

[a]Rated on a 5-point Likert scale (1 = *not at all confident*, 5 = *extremely confident*).
[b]Median and 25th and 75th percentiles reported because data are not normally distributed.

Copyright © 2024 Niebuhr et al. This is an open-access publication distributed under the terms of the Creative Commons Attribution license.

no suggestions to eliminate any specific element. Participants consistently appreciated the takeaway packet/toolkit.

Regarding process, participants appreciated the opportunity to reflect on aspects of their own pending transitions and hear about others' experiences. Several participants asked for more accounts of facilitators' personal experiences. Many participants expressed concern that 2 hours was insufficient to adequately address the topics. They wanted more time for group discussion and/or private time for problem-solving and personal reflection. Several participants commented that the qualities of the facilitators were the most important aspects, describing them as “experts,” and as “comfortable, social and warm.” Several suggested more facilitator participation in the small groups.

Although there was no specific question about outcomes on the immediate postworkshop survey, many participants mentioned specific personal outcomes. These included taking time to reflect and introspect, finding a starting point, sharing with others, realizing one is not alone in confusion or angst, and experiencing new concepts. Outcomes and specific examples are summarized in Table 2.

Summary comments were overwhelmingly positive. Examples include: “Wish I had experienced the workshop 5-10 years ago,” “Looking forward to learning and leading in this space,” “Do it again. I would invite my colleagues,” and “Far exceeded my expectations.”

Discussion

Senior-career faculty contemplating or approaching late-career changes, including part-time or retirement, find a paucity of support for their decision-making. In response, we developed a workshop to facilitate deliberation and discussion among mid- and senior-career faculty in various stages of career transition planning. The workshop includes a variety of activities—metaphor creation, card sorting, personal framing of challenges and priorities, small-group discussions, and personal action planning. We have presented here our methods and evaluations after six workshop iterations. A strength of this workshop is that it has been successfully peer-reviewed four times for presentation to national audiences.

Over the six iterations, we made several changes. We eliminated the word retirement from the workshop title to help allay fears that one's attendance at this workshop might prematurely alert others about possible retirement intent. We added workshop content where appropriate and feasible given time constraints. We shortened the didactic presentations, added more discussion time, and added a brief presentation on institution-specific resources. When we learned that participants preferred to discuss their own circumstances rather than hypothetical cases, we shortened, then ultimately eliminated, case presentations. We added a younger full-time academician to our team in response to recommendations to include a “younger voice.” Some requested topics were not added, because, although these topics reflected common concerns of senior-career faculty, they were felt to be beyond the scope of this workshop, including information about negotiating skills, financial issues, and testing for cognitive decline.

Through the process of development, implementation, and evaluation of the workshop, we learned several important lessons. The title of the workshop is important, as it is the title that draws participants. Our title evolved over the six workshop iterations, including eliminating the word retirement. The most recent title is “What to Do Next: A Toolkit for Late-Career Transition Planning.”

Although we have successfully implemented this workshop in 90 minutes, we think that 2 hours is optimal, to allow more time for personal reflection and small-group discussion. Regarding facilitators, a team approach is recommended, ideally with diversity and facilitators at different stages of transition.

We have learned that it is important to know your audience. While this workshop was originally designed for academic

Table 2. Outcomes Noted by Participants

Subtheme	Illustrative Quotes
Taking time to think, reflect, and introspect.	“Very helpful and made me feel better about the fact that I am even thinking about retiring. Thank you so much!”“Space to think and share.”“Having time to think and work individually on goals and then discuss as a group.”
Finding a starting point to the process of planning for transition.	“Planning is key and needs to start early.”“I am inspired to move forward.”
Sharing conversations with others, realizing I am not alone in my confusion or angst about the process.	“Makes you realize you are not isolated in your concerns.”“Recognizing that my experiences with transitioning are consistent with those of others.”“Realizing that I am not alone in figuring out what comes next.”
Learning/experiencing new or important concepts.	“New ways of thinking about transition.”“Issues I hadn't thought about.”“Idea of ‘transitioning’ rather than abrupt change.”“Hearing about options and other people's experiences.”“This applies to all career stages . . . many considerations . . . many options.”

Copyright © 2024 Niebuhr et al. This is an open-access publication distributed under the terms of the Creative Commons Attribution license.

physicians, we have modified and implemented it also for other health care educators and nonclinician scientists. We have presented the workshop at national/international professional meetings, at regional faculty development activities, and as a single-institution faculty support activity. It is important to know your audience and adjust language and details to fit that audience. For example, academicians who have significant clinical responsibilities may be concerned about issues of clinical competence and loss of their identity as clinicians, whereas academicians whose primary focus is research may be more concerned about issues of succession planning, continued funding, and future success of their research laboratories.

It is also important to be sensitive to participants' perceived vulnerability if others learn of their retirement considerations. Our participants seemed freer to reflect and share when they did not know each other, such as at regional and national/international meetings. At a single institution, where participants were more likely to know each other, there seemed to be more caution about sharing thoughts and feelings about retiring. Sensitivity to this difference is important. In addition, workshop leaders need to recognize that participants may include departmental or institutional leaders attending to learn how to help their own faculty. This workshop does provide career-transition tools that can be helpful for supporting other faculty.

The packet/toolkit is an essential part of this workshop, facilitating active participation and learning during the workshop and guiding continued work afterwards. We recommend making packet/toolkit materials accessible to each participant during the workshop, with paper still being best for hands-on work. QR codes are best for postworkshop access to evaluation instruments. We welcome others to use our materials to conduct this workshop at their own institutions, granting permission for modification of our materials with acknowledgement.

Our workshop evaluation approach has a few limitations. Contacting participants prior to the workshop was not feasible, and we chose not to use limited workshop time to complete a preworkshop evaluation. Therefore, we could not perform an analysis of pre/post change in confidence levels. For four of the six workshops, participants were primarily medical school faculty from a single specialty (pediatrics), which may limit generalizability of our findings.

This highly interactive workshop provides support and experience with practical tools for faculty considering a late-career transition. Participants expressed gratitude for the opportunity to have guided experiential learning in this often forgotten element of career trajectory. They reported that learning and experiencing new concepts in a supportive environment had allowed them to find a starting point for planning their own transition.

In addition to providing resources for individual faculty, this workshop has value for institutions to support their senior-level faculty. Others have reported the need for institutional resources and have shared dissemination approaches (e.g., Palmer and colleagues case-based workshop,[8] Cain's report of a robust program for senior-career faculty,[16] and Baumgartner's report of a new initiative[3]). However, many institutions have focused their faculty development efforts almost exclusively on early-career, junior-level faculty, often ignoring the other end of the career spectrum. We have demonstrated effectiveness of a workshop approach for specifically supporting senior-career faculty preparing for late-career transitions. We encourage the incorporation of this workshop into institutional faculty development programming to ensure a career lifespan approach to faculty development.

Appendices

A. Packet-Toolkit.docx

B. Toolkit Slides.pptx

C. Facilitators Guide.docx

D. Workshop Evaluation.docx

All appendices are peer reviewed as integral parts of the Original Publication.

Virginia Niebuhr, PhD: Clinical Associate Professor, Department of Pediatrics, University of Texas Medical Branch

Pamela Wood, MD: Professor Emeritus, Department of Pediatrics, University of Texas Health Science Center at San Antonio

Judith Livingston, PhD: Retired Assistant Professor, Department of Pediatrics, University of Texas Health Science Center at San Antonio

Fred Henretig, MD, MSHP: Professor Emeritus, Department of Pediatrics, Perelman School of Medicine, University of Pennsylvania

Andrew Sirotnak, MD: Professor, Department of Pediatrics, University of Colorado School of Medicine

Janet Williams, MD: Professor, Department of Pediatrics, University of Texas Health Science Center at San Antonio

David Jaffe, MD: Retired Vice President of Education, American Academy of Pediatrics; Former Professor of Pediatrics and Emergency Medicine, UCSF School of Medicine

Copyright © 2024 Niebuhr et al. This is an open-access publication distributed under the terms of the Creative Commons Attribution license.

Acknowledgments
We acknowledge the contribution of Karen Szauter, MD, University of Texas Medical Branch, participant at workshop #4 and contributor to the literature review and reference list. We acknowledge the generous contribution of our workshop participants for their honest evaluations.

Disclosures
None to report.

Funding/Support
None to report.

Prior Presentations
Wood P, Niebuhr V, Jaffe D, Livingston J, Henretig F. Late career transitions. Presented at: Pediatric Academic Societies; November 28, 2019; Baltimore, MD.

Wood P, Niebuhr V, Jaffe D, Livingston J, Henretig F. It's never too early to plan forward: a toolkit for late career transitions. Presented virtually at: Pediatric Academic Societies Meeting; June 1, 2021.

Niebuhr V, Wood P, Livingston J, Williams J, Szauter K. It's never too early to plan: a toolkit for educators' late career transitions. Presented at: University of Texas System Shine Academy Annual Meeting; February 25, 2022; Austin, TX.

Wood P, Niebuhr V, Henretig F, Jaffe D. It's never too early to plan: a toolkit for educators' late career transitions. Presented at: Pediatric Academic Societies Meeting; April 29, 2022; Denver, CO.

Wood P, Niebuhr V, Henretig F, Jaffe D, Sirotnak A. It's never too early to plan: a toolkit for educators' late career transitions. Pediatric Academic Societies Meeting; April 29, 2023; Washington, DC.

Ethical Approval
The University of Texas Health Science Center at San Antonio Institutional Review Board deemed further review of this project not necessary.

References

1. *Trends: Average Age of Chairs and Full-time Faculty by Gender and Rank, 1966 Through 2023*. Association of American Medical Colleges. Updated December 31, 2023. Accessed October 21, 2024. https://www.aamc.org/media/54641/download
2. *Supplemental Table F: U.S. Medical School Faculty by Gender, Rank, and Age Group, 2022*. Association of American Medical Colleges. Updated December 31, 2022. Accessed October 21, 2024. https://www.aamc.org/media/72546/download
3. Baumgartner W, Ziminski C, Van Beek J, Rand C. Everyone wins when retiring faculty of health professions schools are supported. *Acad Med*. 2021;96(10):1375-1376. https://doi.org/10.1097/ACM.0000000000004147
4. Plotnick LH, Sternszus R, Macdonald ME, Steinert Y. Engaging retired physicians as educators: motivations and experiences of participants in a novel educational program. *Acad Med*. 2022; 97(12):1841-1846. https://doi.org/10.1097/ACM.0000000000004981
5. Altman Y, Baruch Y, Zoghbi Manrique-de-Lara P, Viera Armas MM. Baby boomers at the cusp of their academic career: *Storming ahead, Hanging on, or Calling it a day*. *Stud High Educ*. 2020;45(7):1335-1350. https://doi.org/10.1080/03075079.2019.1610864
6. Onyura B, Bohnen J, Wasylenki D, et al. Reimagining the self at late-career transitions: how identity threat influences academic physicians' retirement considerations. *Acad Med*. 2015;90(6): 794-801. https://doi.org/10.1097/ACM.0000000000000718
7. Thorndyke L, Grayson M, Gusic M. Navigating the transition to a new leadership position: moving on and moving up. *MedEdPORTAL*. 2014;10:9707. https://doi.org/10.15766/mep_2374-8265.9707
8. Palmer M, Welch J, McAteer J, Walvoord E. Ensuring the vitality of senior and emeritus faculty: a leadership development workshop. *MedEdPORTAL*. 2015;11:10267. https://doi.org/10.15766/mep_2374-8265.10267
9. Schlossberg NK. *Revitalizing Retirement: Reshaping Your Identity, Relationships, and Purpose*. American Psychological Association; 2009.
10. Anderson ML, Goodman J, Schlossberg NK. *Counseling Adults in Transition: Linking Schlossberg's Theory With Practice in a Diverse World*. 4th ed. Springer; 2012.
11. Bridges W. *Transitions: Making Sense of Life's Changes*. 2nd ed. Da Capo Press; 2004.
12. Skarupski KA, Welch C, Dandar V, Mylona E, Chatterjee A, Singh M. Late-career expectations: a survey of full-time faculty members who are 55 or older at 14 U.S. medical schools. *Acad Med*. 2020;95(2):226-233. https://doi.org/10.1097/ACM.0000000000002847
13. Skarupski KA, Dandar V, Mylona E, Chatterjee A, Welch C, Singh M. Late-career faculty: a survey of faculty affairs and faculty development leaders of U.S. medical schools. *Acad Med*. 2020; 95(2):234-240. https://doi.org/10.1097/ACM.0000000000002849
14. Sugarman L. *Life-Span Development: Frameworks, Accounts and Strategies*. 2nd ed. Taylor & Francis; 2001.
15. Glesne C. *Becoming Qualitative Researchers: An Introduction*. 4th ed. Pearson; 2011.
16. Cain JM, Felice ME, Ockene JK, et al. Meeting the late-career needs of faculty transitioning through retirement: one institution's approach. *Acad Med*. 2018;93(3):435-439. https://doi.org/10.1097/ACM.0000000000001905

Received: April 23, 2024
Accepted: July 29, 2024
Published: November 12, 2024

Copyright © 2024 Niebuhr et al. This is an open-access publication distributed under the terms of the Creative Commons Attribution license.
